# Oxidation of Alcohols to Carboxylates with N_2_O Catalyzed by Ruthenium(II)-CNC Complexes

**DOI:** 10.1021/acscatal.5c02021

**Published:** 2025-06-20

**Authors:** José Bermejo, Laura L. Santos, Eleuterio Álvarez, Joaquín López-Serrano, Andrés Suárez

**Affiliations:** a Instituto de Investigaciones Químicas (IIQ) and Centro de Innovación en Química Avanzada (ORFEO−CINQA), CSIC and Universidad de Sevilla, Avda Américo Vespucio, 49, Sevilla 41092, Spain; b Instituto de Investigaciones Químicas (IIQ), CSIC and Universidad de Sevilla, Avda Américo Vespucio, 49, Sevilla 41092, Spain

**Keywords:** nitrous oxide, transfer hydrogenation, alcohols, carboxylates, ruthenium complexes

## Abstract

Air-stable ruthenium­(II) complexes based on a picoline-derived CNC pincer ligand, [RuH­(CNC)­(CO)*L*]*X* (*L* = PPh_3_, *X* = Br; *L* = CO, *X* = BF_4_), were found to catalyze under basic conditions the oxidation with N_2_O of a series of alcohols to carboxylates. Both [RuH­(CNC)­(CO)*L*]*X* complexes react readily with strong bases (*t*BuOK or KHMDS), giving rise to a Ru­(II) complex containing a deprotonated CNC* ligand (when *L* = PPh_3_) or a Ru(0)-CNC derivative (for *L* = CO). Furthermore, the mechanism of the catalytic reaction has been elucidated through density functional theory (DFT) calculations. The catalytic cycle has been shown to proceed through an outer-sphere mechanism comprising four key transformations, which involve Ru­(II) intermediates based on the deprotonated CNC* ligand: (i) alkoxide dehydrogenation to yield a Ru­(II) hydride complex and an aldehyde molecule, (ii) N_2_O insertion into the ruthenium–hydride bond to yield a hydroxy ruthenium species and N_2_, (iii) nucleophilic attack of the hydroxo ligand in the Ru–OH complex to the intermediate aldehyde, and (iv) dehydrogenation of the formed alcoholate to regenerate the catalytically active Ru­(II) hydride and produce the carboxylate product.

## Graphical abstract

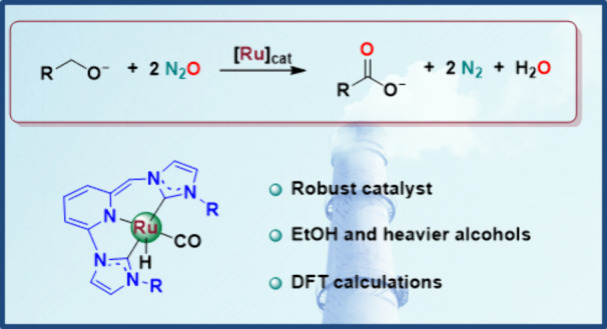

## Introduction

Nitrous oxide is regarded as an environmental damaging greenhouse gas, characterized by a high global warming potential and a long lifetime of ca. 120 years in the atmosphere.^1^ In addition, N_2_O is currently regarded as one of the major contributors to the degradation of the ozone layer.^2^ The rising anthropogenic atmospheric concentrations of N_2_O are primarily derived from the widespread use of fertilizers, fuels combustion, and emissions associated with the industrial synthesis of chemicals such as adipic acid and nitric acid.^3^ As a result, the upward N_2_O concentration trend in the atmosphere over the past decades has drawn significant attention, and diverse approaches aimed to mitigating its increase have been examined.^4^ These strategies are primarily focused on either reducing N_2_O emissions or to facilitate its conversion into chemical derivatives that exhibit negligible impact in the environment. In the context of the circular economy, it is particularly appealing to develop efficient chemical transformations for N_2_O degradation that allow its revalorization as a chemical feedstock.^5^

Metal-catalyzed transformations of N_2_O to innocuous nitrogen (N_2_) are critically constraint by several limitations. First, although direct decomposition of N_2_O to nitrogen (N_2_) and oxygen (O_2_) is thermodynamically favorable, the process is hindered by substantial kinetic barriers.^6^ Moreover, development of catalytic processes mediated by transition metal complexes is limited by the N_2_O ligand weak σ-donor and π-acceptor characteristics, and its strong oxidizing properties.^7^ In fact, only a limited number of stable metal complexes featuring coordinated nitrous oxide has been reported.^8^ In striking contrast, N_2_O oxygen atom transfer to diverse reducing agents, including H_2_,^9–11^ CO,^12^ phosphines,^13^ boranes,^14,15^ silanes,^9,16^ and hydrocarbons,^17^ has been accomplished by the use of homogeneous metal catalysts under relatively mild conditions.

Alcohols are widely utilized as reductants in both academic research and industrial applications, enabling the efficient reduction of a broad range of substrates, including small gaseous molecules such as CO_2_,^18^ which is isoelectronic with N_2_O.^19^ Use of alcohols instead of H_2_ in reduction processes minimizes the need of pressure equipment and, in the case of reactions with N_2_O, the formation of potentially explosive atmospheres.^20^ In 2016, Gianetti, Reiher, Grützmacher et al. made use of a Rh species stabilized with a bis­(olefin)­amido ligand to catalyze the dehydrogenative coupling of alcohols, utilizing N_2_O as the hydrogen acceptor.^21^ A series of alcohols were conveniently transformed to the corresponding carboxylates, while the same reactions carried out with H_2_O trapping led to the isolation of ester derivatives. More recently, Trincado, Grützmacher et al. employed low-valent dinuclear ruthenium complexes to form carboxylates through N_2_O oxidation of alcohols, including lightweight abundant ethanol.^22^ Oxidation of alcohols with N_2_O to aldehydes and ketones, and of aldehydes to carboxylic acids, have also received wide attention.^23^

Herein, we report the oxidation of alcohols to carboxylates with N_2_O using Ru complexes containing a picoline-derived *N*-heterocyclic carbene pincer CNC ligand as catalysts. The most active catalyst was found to promote the oxidation of a series of primary alcohols, including ethanol, exhibiting pronounced activity and selectivity. In addition, the investigation of the mechanism of the reaction via DFT calculations supported an outer-sphere catalytic cycle based on Ru­(II) species incorporating the deprotonated CNC* ligand.

## Results and Discussion

### Synthesis of Ru-CNC Complexes

The synthesis of the picoline-derived CNC ligand precursor was accomplished by reaction of the imidazolium derivative **1** ^15^ and 1-mesityl-1*H*-imidazole (Scheme 1). Subsequent treatment of **2** with Ag_2_O yielded the silver complex **3**, as evinced through ^1^H NMR spectroscopy by the loss of the signals of the imidazolium H-2 hydrogens (ca. 11 ppm) and the occurrence, in the ^13^C­{^1^H} NMR experiment, of slightly broad resonances caused by the C-2 carbons of the carbene donors (ca. 184 ppm).^24^ In turn, the ruthenium complex **4** was synthesized following a transmetalation procedure through reaction of the Ag-NHC complex **3** and RuHCl­(CO)­(PPh_3_)_3_ in THF, followed by treatment with KBr (Scheme 1). Complex **4** was isolated in 86% yield as a yellow solid and found to be stable under atmospheric conditions. In the ^1^H NMR spectrum of **4**, the Ru–H hydrogen produces a doublet resonance appearing at –8.49 ppm and characterized by a *J*_HP_ of 88.2 Hz, evincing the *trans* coordination of the PPh_3_ and hydrido ligands. Meanwhile, the methylene CH_2_ arm causes two coupled doublet signals at 4.26 and 5.89 ppm (^2^ *J*_HH_ = 15.7 Hz). Meanwhile, one doublet for each C-2 carbene atom at 188.6 (*J*_CP_ = 8 Hz) and 200.0 ppm (*J*_CP_ = 6 Hz), and another doublet attributable to the CO ligand appearing at 205.6 ppm (*J*_CP_ = 8 Hz), were observed in the ^13^C NMR experiment. The coordinated CO ligand in complex **4** is also evidenced by the observation of an intense band at 1951 cm^–1^ in the IR spectrum, attributable to the CO stretching mode. Finally, the ^31^P­{^1^H} NMR spectrum of complex **4** displays a singlet resonance at 28.2 ppm. Crystals for X-ray diffraction analysis adequate for the study of the cationic fragment of **4**, **4** ^**+**^, were obtained before treatment of the reaction mixture with KBr, and confirmed the proposed structure (Figure 1).

**Scheme 1:**
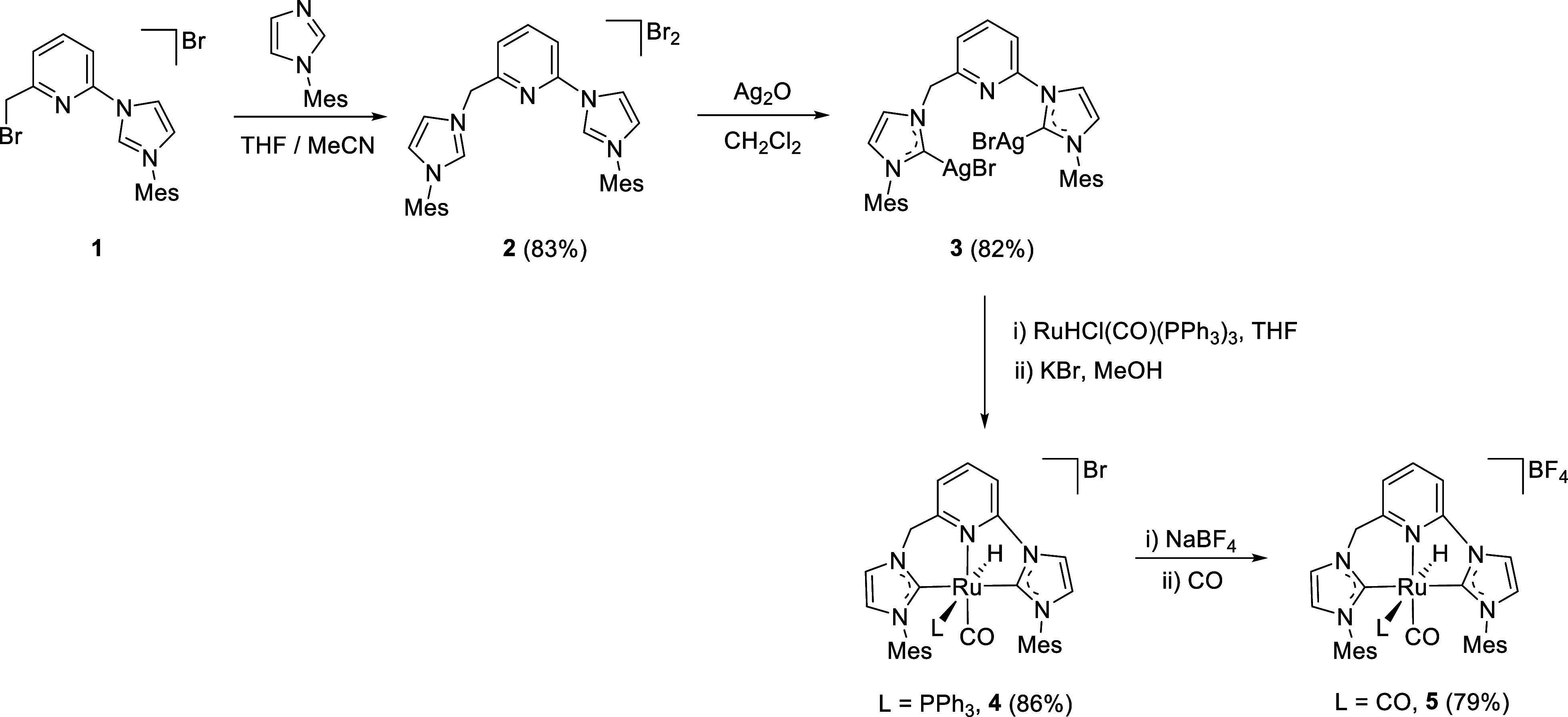
Synthesis of the Bis-imidazolium Salt (**2**), and Silver (**3**) and Ruthenium (**4** and **5**) Complexes

**Figure 1:**
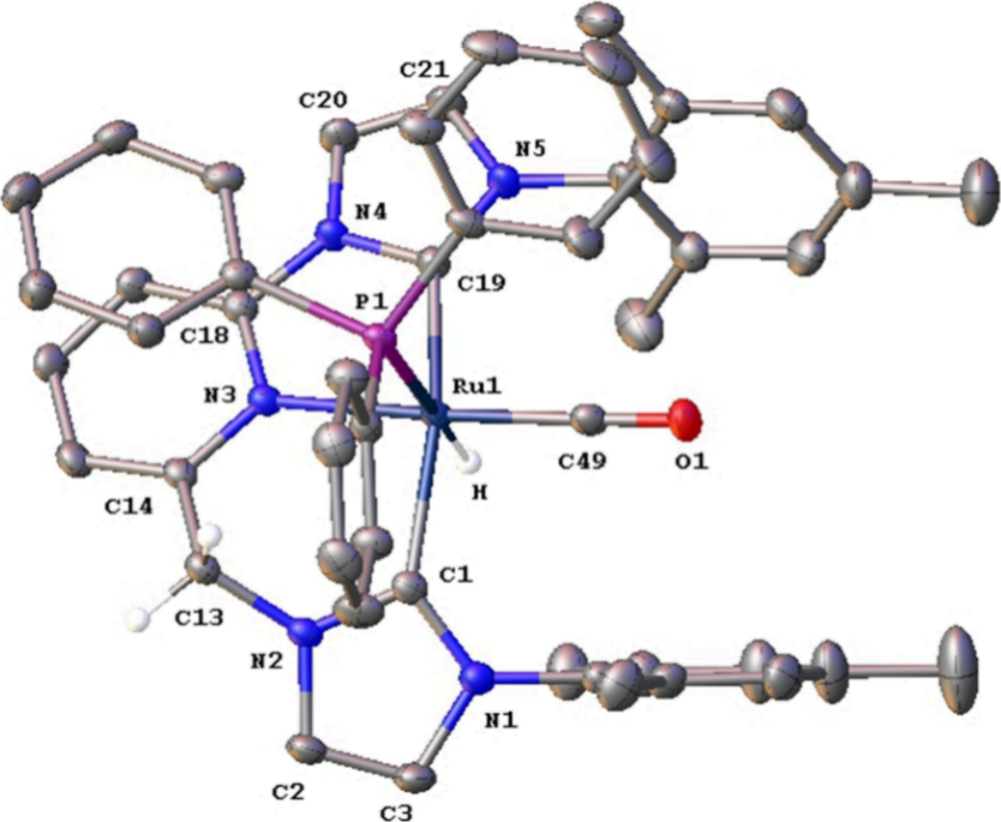
ORTEP perspective (50% ellipsoid probability) of the cationic fragment **4** ^**+**^. Counteranion is a 9:1 mixture of Cl^–^ and Br^–^, respectively. Hydrogen atoms, with exception of the hydrido ligand and the methylene hydrogens, have been omitted for clarity. Selected bond lengths [Å] and angles [deg]: Ru(1)–N(3) 2.1401(19), Ru(1)–C(1) 2.086(2), Ru(1)–C(19) 2.046(2), Ru(1)–C(49) 1.848(3), Ru(1)–P(1) 2.4502(6), C(1)–Ru(1)–C(19) 156.04(9), C(1)–Ru(1)–N(3) 88.26(8), C(19)–Ru(1)–N(3) 76.75(8), C(49)–Ru(1)–N(3) 172.24(9), C(1)–Ru(1)–P(1) 103.58(7), C(19)–Ru(1)–P(1) 94.89(7), C(1)–Ru(1)–C(49) 95.59(10), C(19)–Ru(1)–C(49) 97.36(10), C(49)–Ru(1)–P(1) 96.47­(8), N(3)–Ru(1)–P(1) 89.14(5).

The PPh_3_ ligand in complex **4** was found to be readily displaced by CO. Hence, treatment of **4** with NaBF_4_, followed by exposure to CO (3 bar) of a solution in THF of the resulting derivative, provided the cationic dicarbonyl complex **5**. This species was isolated in 79% yield as a yellow solid, and exhibited stability when exposed to air as a solid. The ^1^H NMR spectrum of compound **5** shows spectroscopic characteristics closely resembling those of **4**, with the prominent exception of the absence of resonances attributable to PPh_3_. For example, the presence of the Ru–H hydrogen is manifested by a singlet signal at –6.28 ppm. Moreover, the resonances of the C-2 carbene atoms appear as singlet signals at 193.8 and 181.7 ppm in the ^13^C­{^1^H} NMR experiment, whereas the CO ligands produce two singlets appearing at 200.1 and 194.3 ppm in the same spectrum. The presence of two carbonyl ligands was further supported by intense IR absorption bands at 2036 and 1985 cm^–1^.

### Catalytic Oxidation of Alcohols with N_2_O

Complexes **4** and **5** were tested as catalyst precursors in the oxidation of alcohols with N_2_O. Initial catalytic experiments were performed with solutions of 1-hexanol, *t*BuOK (1.2 equiv) and complex **4** (1.0 mol %) in different solvents under reflux using 1.0 bar of N_2_O (entries 1–3, Table 1). Among the examined solvents, reactions carried out in toluene were found to provide the highest yields of carboxylate. Moreover, in this solvent, complex **5** exhibited a slightly lower catalytic activity than **4** (entry 4). In these reactions, N_2_ generation was verified through gas chromatography (GC-TCD) analysis of the gas atmosphere. In addition, no effect of the presence of Hg in the catalytic process was noted, in agreement with the occurrence of a homogeneously catalyzed process.

**Table 1:**
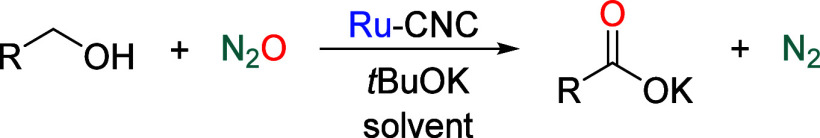

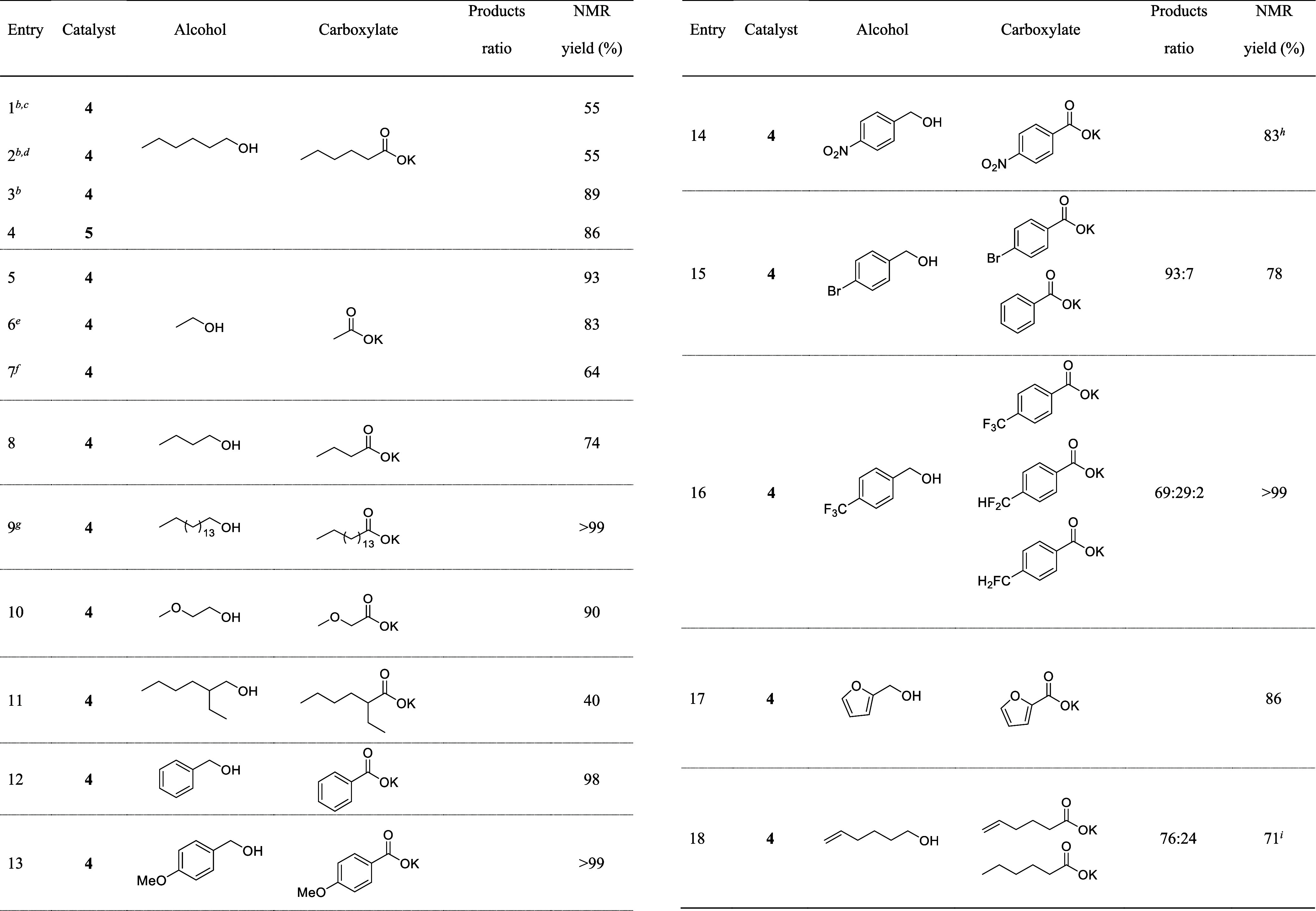
Oxidation of Alcohols with N_2_O Catalyzed by Ru-CNC Complexes^a^

a Reaction conditions: 1.0 mol % Ru, 1.2 equiv *t*BuOK, 1.0 bar N_2_O (ca. 5 equiv), 120 °C, toluene. [S] = 0.6 M. 24 h. Conversion was determined by ^1^H NMR spectroscopy using an internal standard.

b 20 h.

c 2-Methyltetrahydrofuran, 100 °C.

d Dioxane, 110 °C.

e 0.4 mol % Ru. Reaction time: 23 h.

f 0.2 mol % Ru. Reaction time: 48 h.

g 2.0 mol % Ru. Reaction time: 36 h.

h Formation of ca. 13% of nitro group reduction products was observed.

i Formation of 12% of alkene isomerization products was observed.

Having optimized the catalytic reaction conditions, a series of alcohols were further tested. First, ethanol was oxidized to potassium acetate with 93% yield (entry 5). This transformation is particularly appealing due to the use of ethanol, an abundant biosourced derivative,^25^ as reductant of N_2_O and the concomitant formation of acetic acid derivative, a highly demanded chemical commodity.^26^ As far as we are aware, only two other catalytic systems have been exploited for such a process. Kagiya et al. demonstrated the reduction of N_2_O with ethanol using platinum nanoparticles at high catalysts loadings;^27^ meanwhile, Trincado, Grützmacher et al. employed a binuclear Ru complex, which exhibited TON values of up to 480 based on the catalyst precursor molecule.^22^ By lowering the catalyst loadings up to 0.2 mol %, TON values of up to 320 (with respect to the total metal content) were achieved with complex **4** (TOF = 6.7 h^–1^) (entries 6 and 7).

Next, the influence of the alcohol chain length was further investigated. As in the case of ethanol, butanol and hexadecanol, a long-chain fatty alcohol, were oxidized with high conversions (entries 8 and 9). Moreover, use of 2-methoxyethanol allowed to obtain the corresponding carboxylate in high yield (entry 10). Meanwhile, the reaction of 2-ethyl-hexanol, a β-branched alcohol, proceeded with a moderate conversion (entry 11).

The oxidation of benzyl alcohols was next examined (entries 12–16). Derivatives bearing both electron-releasing and -withdrawing groups were oxidized with high yields (>78%). However, the nitro group was partially reduced in the reaction of 4-nitrobenzyl alcohol with N_2_O, and partial halogen/hydrogen exchange occurred in the case of 4-bromo- and 4-trifluoromethyl substituted derivatives. In addition, furfuryl alcohol, a derivative of the biomass-derived furfural, was converted to 2-furoic acid in 86% NMR yield (entry 17). Finally, the oxidation of 5-hexen-1-ol, a substrate containing a C=C double bond, was investigated (entry 18). Although this derivative was oxidized with high conversion, significant reduction and isomerization of the remote olefinic bond was observed.

### Catalyst Activation

Aiming to determine the metal species generated under the basic conditions of the catalytic reactions, the reactivity of the Ru­(II)-CNC complexes **4** and **5** with strong bases was investigated. Complex **4** cleanly reacted with *t*BuOK or KHMDS in THF-*d*_8_ providing the deprotonated derivative **6** (Scheme 2). The ^1^H NMR spectrum of this complex displays a doublet at –8.38 ppm (*J*_HP_ = 121.5 Hz), corresponding to the Ru–H ligand, while the CNC vinylic proton produces a singlet at 5.55 ppm. In addition, significant upfield shifts, with respect to **4**, of the signals of the central ring protons, which are shown between 5.38 and 6.18 ppm, are observed. The CO ligand gives rise to a relatively broad resonance centered at 207.9 ppm in the ^13^C­{^1^H} NMR spectrum, while resonances corresponding to the carbenic carbon atoms are exhibited as doublets at 200.3 ppm (*J*_CP_ = 7 Hz) and 182.2 ppm (*J*_CP_ = 9 Hz). In the ^31^P­{^1^H} NMR experiment, coordination of PPh_3_ is evidenced by a singlet resonance appearing to 26.0 ppm. Finally, the CO stretching mode absorption in the IR spectrum of **6** appears at lower wavelengths (1921 cm^–1^) than in its protonated counterpart.

**Scheme 2:**
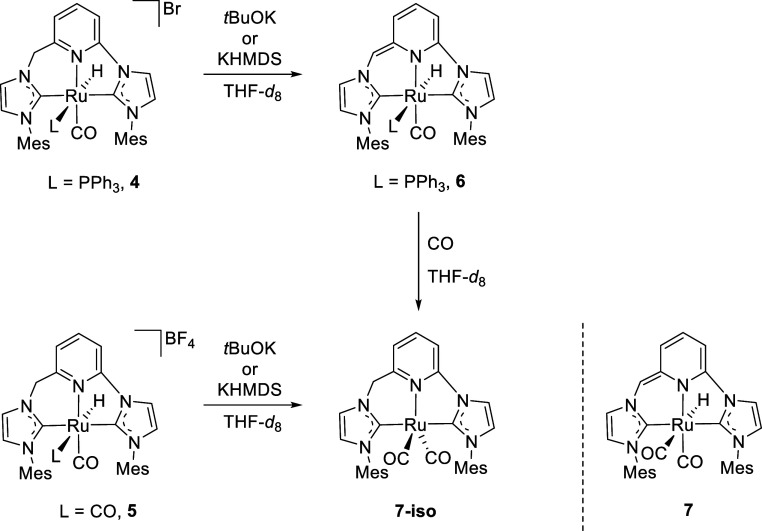
Reactions of Ru­(II)-CNC Complexes **4** and **5** with Strong Bases

To further confirm the proposed structure of complex **6**, X-ray quality crystals were grown from a THF solution through slow evaporation (Figure 2). The structure of **6** comprises a distorted octahedral geometry, wherein the deprotonated CNC* ligand coordinates in a meridional fashion (C–Ru–C = 162.35(13)°). The carbonyl ligand occupies a position *trans* to the donating nitrogen atom. The Ru–C^2^(NHC) bond lengths of 2.073 Å and 2.050 Å fall within the range of values previously reported (1.967–2.168 Å).^28^ Moreover, the solid state structure of **6** reveals the dearomatization of the former pyridine ring, as a reduced C_Py_–CH­(NHC) (C­(13)–C­(14) = 1.322(5) Å) bond distance is observed in comparison to the C_Py_-CH_2_(NHC) (C(13)–C(14) = 1.51 Å) length in **4**, along with altered C–C bond lengths in the central ring characterized by an alternation between elongated (1.44–1.46 Å) and shortened bonds (1.31–1.33 Å) (average C–C bond in the pyridine ring of **4** ^**+**^: 1.38 Å).^11,29^

**Figure 2:**
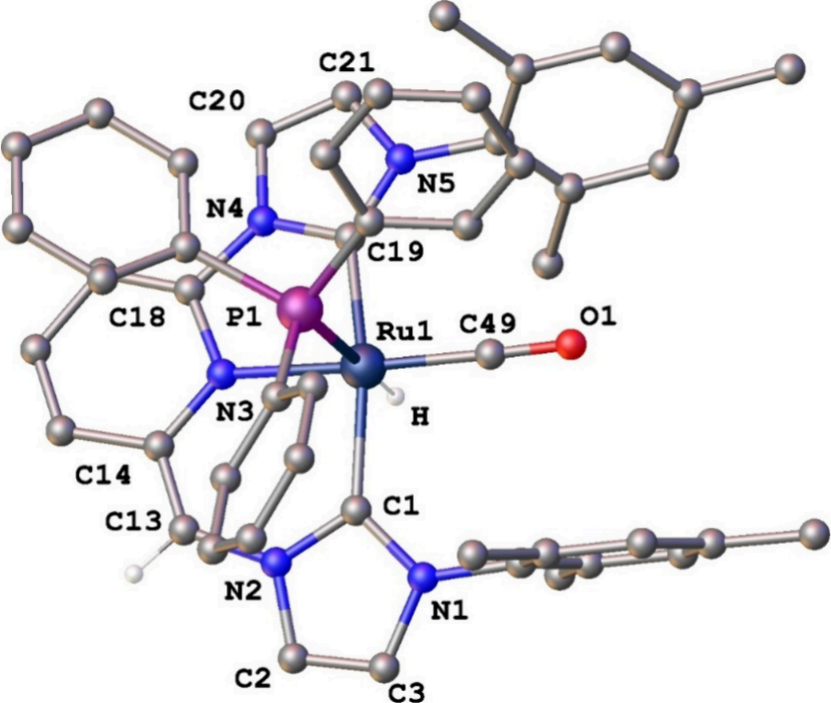
ORTEP perspective (50% ellipsoid probability) of complex **6**. Hydrogen atoms, with exception of the hydrido ligand and the methine hydrogen, have been omitted for clarity. Selected bond lengths [Å] and angles [deg]: Ru(1)–N(3) 2.133(3), Ru(1)–C(1) 2.073(3), Ru(1)–C(19) 2.050(3), Ru(1)–C(49) 1.841(3), Ru(1)–P(1) 2.4602(8), C(1)–Ru(1)–C(19) 162.35(13), C(1)–Ru(1)–N(3) 88.60(13), C(19)–Ru(1)–N(3) 78.31(13), C(49)–Ru(1)–N(3) 170.76(13), C(1)–Ru(1)–P(1) 97.44(9), C(19)–Ru(1)–P(1) 94.20(9), C(1)–Ru(1)–C(49) 95.71(14), C(19)–Ru(1)–C(49) 95.69(14), C(49)–Ru(1)–P(1) 98.26(10), N(3)–Ru(1)–P(1) 89.25(8).

As in the case of complex **4**, the reaction of derivative **5** with base was investigated (Scheme 2). Treatment of **5** with KHMDS in THF-*d*_8_ led to the initial observation, after 10 min, of resonances corresponding to three species that were attributed to the Ru-CNC* hydride **7** (52%), the Ru(0)-CNC species **7-iso** (33%) and an unidentified complex (15%). Subsequent follow-up of the reaction revealed complete transformation of **7** to the Ru(0) derivative **7-iso** within less than 1 h. Interestingly, the clean formation of complex **7-iso**, which was found to be extremely sensitive, was also observed in the reaction of the *in situ* deprotonated derivative **6** with CO (Scheme 2). NMR analysis of the dark blue solutions of **7-iso** exhibits the expected signals corresponding to the protonated CNC ligand. In the ^13^C­{^1^H} NMR spectrum, the resonance attributable to the CO ligands appears at 211.0 ppm, and those of the C^2^–NHC carbons are observed at 200.2 and 193.8 ppm.

The catalytic competence of Ru(0) pincer complexes has been established in both the hydrogenation of polar C=X bonds (X = O, N) and in alcohol dehydrogenation reactions.^30–33^ Moreover, examples of pincer Ru(0) complexes that are able to interconvert to catalytically active Ru­(II) species have been previously reported.^34,35^ As both complexes **4** and **5** were found to be active in the catalytic oxidation of alcohols with N_2_O, we turned to density functional theory (DFT; SMD­(THF)-B3LYP-D3/def2TZVP) calculations to determine the feasibility of Ru­(II)-CNC*/Ru(0)-CNC interconversion (Figure 3). Initial comparison of the thermodynamic stabilities of the **6**/**6-iso** and **7**/**7-iso** tautomer pairs indicates that, while **6** and **6-iso** are essentially isoenergetic (Δ*G* = 0.9 kcal/mol), the Ru­(II) species **7** is 2.9 kcal/mol less stable than its Ru(0) counterpart **7-iso**. The higher stability of **7-iso**, with respect to **7**, can be expected to derive from the presence of the π-accepting CO ligands that would stabilize the electron-rich Ru(0)-CNC moiety.^34^ Meanwhile, in the case of the **6**/**6-iso** pair, nonattractive interactions of the mesityl substituents with the more bulky PPh_3_ ligand would disfavor the trigonal-bipyramidal coordination geometry of **6-iso**. Migration of the hydrido ligand to the deprotonated CNC* ligand methine arm was calculated to have a barrier of 18.3 kcal/mol for the **6**/**6-iso** tautomers, while this barrier was found to be significantly higher (34.0 kcal/mol) for the conversion of **7** into **7-iso**. Although the high calculated energy of the transition state associated with the interconversion of **7** and **7-iso** precludes that this process could take place at room temperature, we experimentally observed the initial formation of **7** from the reaction of **6** and CO and its subsequent transformation to **7-iso** (*vide supra*). This process could be assisted by protic species or take place by hydrogen tunneling processes,^36^ as previously proposed for similar proton–hydride tautomerism in metal systems incorporating lutidine-derived ligands.^37,38^

**Figure 3:**
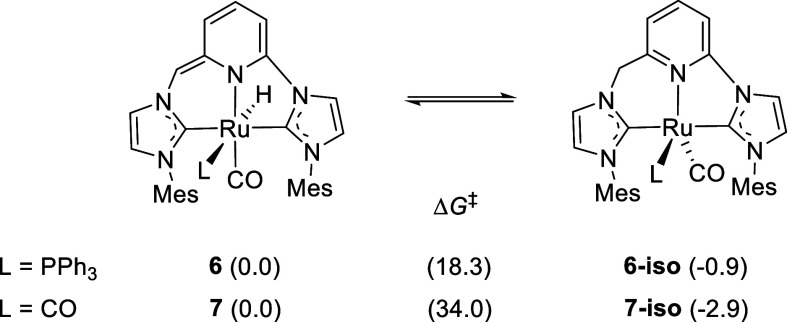
DFT calculated stabilities of the Ru­(II)-CNC* (**6** and **7**) and Ru(0)-CNC (**6-iso** and **7-iso**) complexes, and interconversion energy barriers (Δ*G* ^‡^). In parentheses, energies (Δ*G* in THF) in kcal/mol.

### Catalytic Reaction Mechanism

Assuming that alcohol/N_2_O mixtures are converted under basic conditions according to reaction steps (1a)–(1c) and the overall reaction (2) of Figure 4, additional experiments were conducted to elucidate the potential mechanism. Initially, Ru catalyzed alcohol dehydrogenation was demonstrated by the dimerization of 1-hexanol (Guerbet product),^39^ observed upon heating solutions of the alcohol under the catalytic conditions described above in the absence of N_2_O (Figure 5, (i). Similarly, the dehydrogenation of 1-hexanol in the presence of N_2_O and a water trapping agent (4 Å molecular sieves) took place with 98% conversion, resulting in a mixture of alcohol dehydrogenative coupling and dimerization products (Figure 5, (ii). Next, evidence of the likely occurrence of hydride Ru species was obtained after investigating the reduction of N_2_O with H_2_ using complex **4** as catalyst precursor (Figure 5, (iii).^9–11,22^ In this experiment, reduction of N_2_O to N_2_ was confirmed by analysis of the headspace gas atmosphere by gas chromatography, and the catalyst activity determined by ^1^H NMR spectroscopy analysis of water (TON = 750). Finally, complex **4** was found to catalyze the transformation of benzaldehyde to benzoate using H_2_O as oxidant, which is expected to be formed in the reduction of N_2_O (Figure 5, (iv).^40^

**Figure 4:**
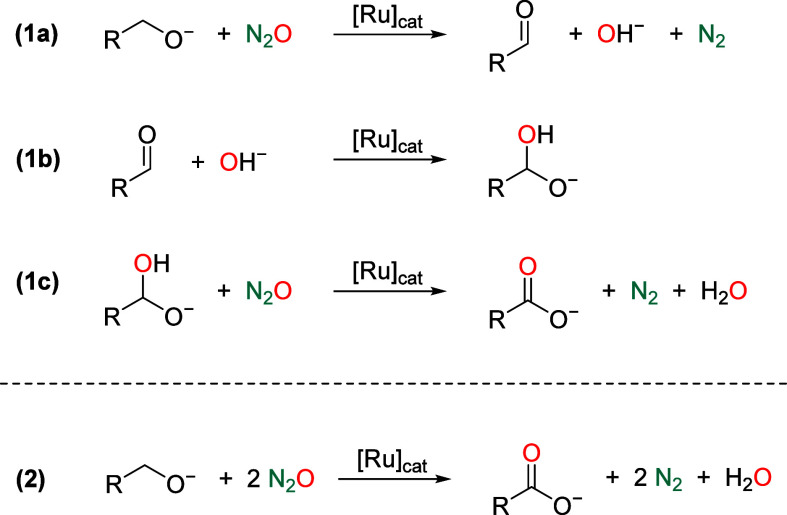
Reaction steps (1a)–(1c) and the overall reaction (2) of the oxidation of alcohols with N_2_O under basic conditions.

**Figure 5:**
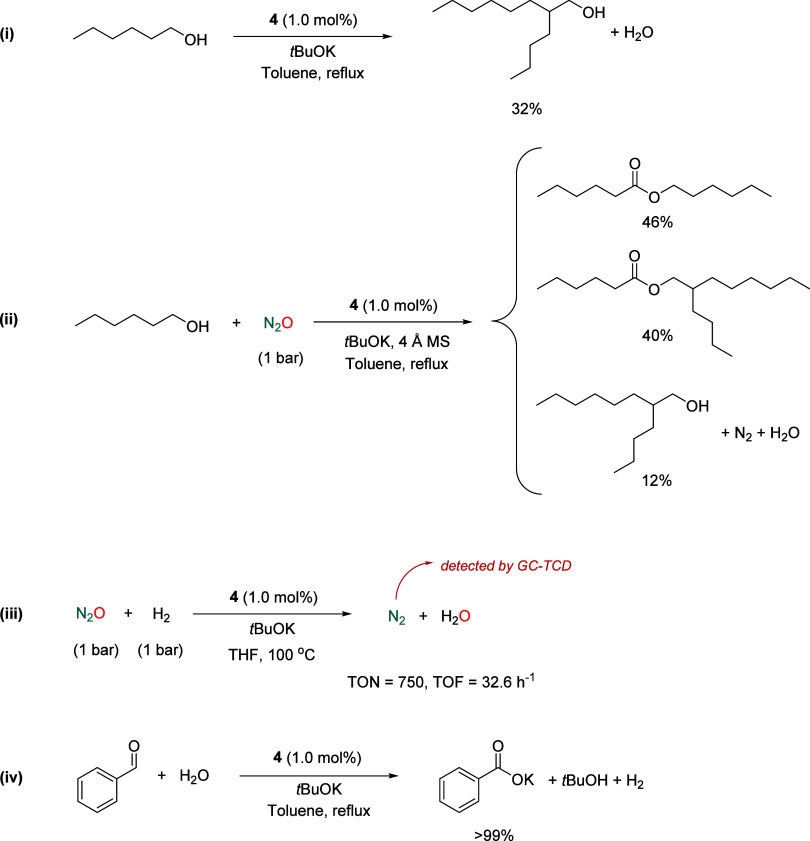
Catalytic control experiments: (i) acceptorless dehydrogenation of 1-hexanol, (ii) oxidation of 1-hexanol with N_2_O in the presence of 4 Å molecular sieves, (iii) hydrogenation of N_2_O, and (iv) oxidation of benzaldehyde with H_2_O.

According to these control experiments, the mechanism of the catalytic reaction may involve the following key transformations (Scheme 3): (i) alcohol dehydrogenation to yield a Ru hydride complex and aldehyde, (ii) reduction of N_2_O by the Ru–H species with formation of N_2_ and a Ru hydroxy intermediate, and (iii) nucleophilic attack of the Ru–OH to the aldehyde molecule to yield an intermediate hemiacetalate, which can be subsequently dehydrogenated to regenerate the Ru–H species. Consequently, we next turned to perform DFT calculations (SMD­(Toluene)-B3LYP-D3/def2TZVP) in order to determine the energy profile of the mechanism of the oxidation of alcohols with N_2_O (Figure S46). Initially, Ru(0)/Ru­(II) and ligand-assisted mechanisms were ruled out on the basis of the high calculated energy barriers (see Supporting Information). As a result, a catalytic cycle involving initial decoordination of the L ligand (L = PPh_3_, CO) in the deprotonated complexes **6** and **7** was considered (Figure 6). From **6**, the free energy involved in the dissociation of PPh_3_ was calculated to be 9.6 kcal/mol, and lead to the formation of the unsaturated species **A**. Similarly, an analogous process was estimated for complex **7**. In this case, CO decoordination to give **A** was found to be endergonic by 14.3 kcal/mol. Subsequent facile hydride transfer from ethoxide to **A** forms the anionic dihydride complex **B**. This process is thermodynamically favorable and takes place through the transition state **T**_**A→B**_ having an energy barrier of merely 2.2 kcal/mol (Figure 7).

**Scheme 3:**
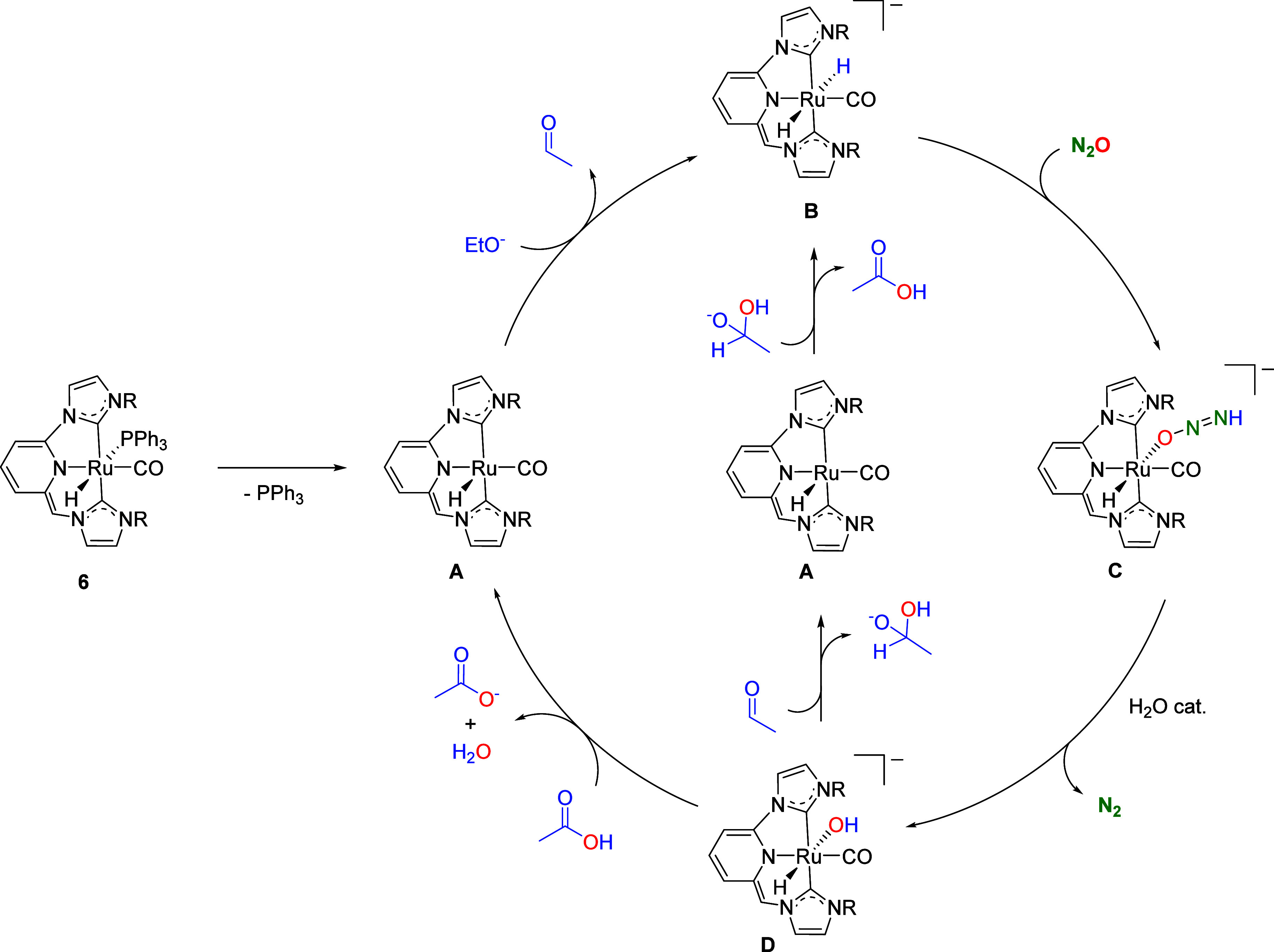
Catalytic Cycle Calculated by DFT for the Oxidation of Ethanol with N_2_O Catalyzed by Complex **6**

**Figure 6:**
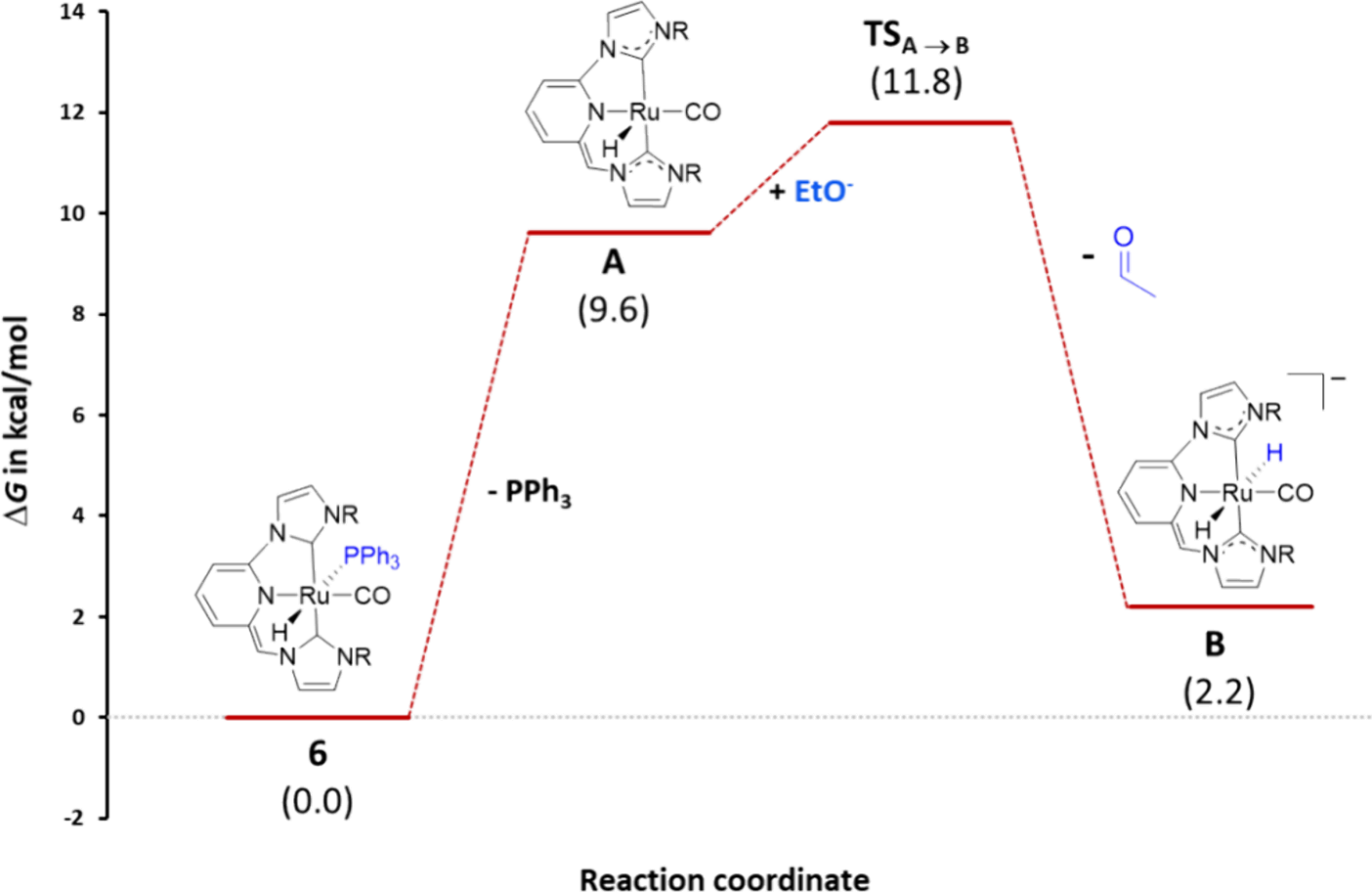
Free energy (Δ*G* in toluene, kcal/mol) profile calculated by DFT for the PPh_3_ dissociation from **6** and alcohol dehydrogenation by **A** (the origin of energies is **6** + EtO^–^ + 2 N_2_O).

**Figure 7:**
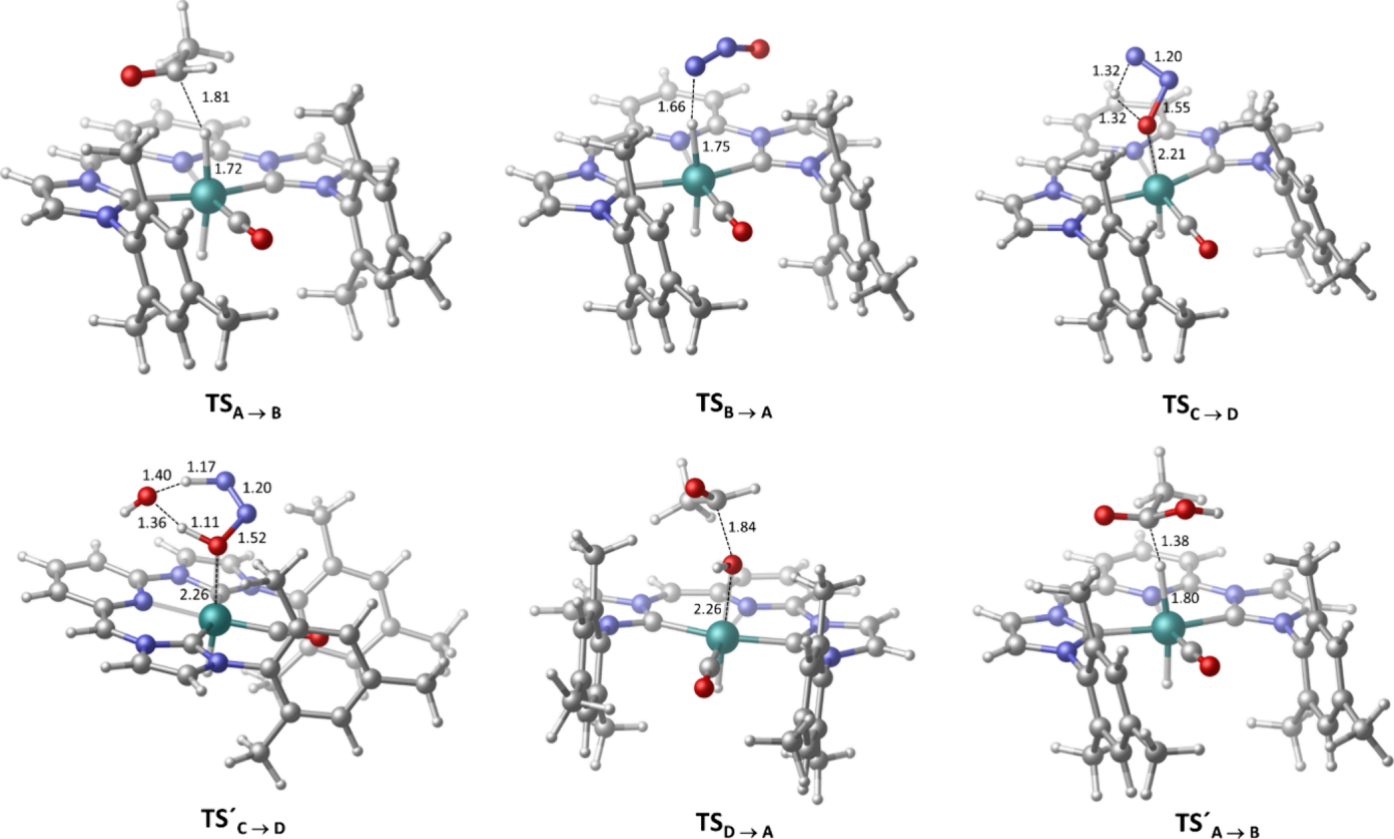
Geometries optimized by DFT of the **TS**_**A→B**_, **TS**_**B→A**_, **TS**_**C→D**_, **TS′**_**C→D**_, **TS**_**D→A**_ and **TS′**_**A→B**_ transition states.

Next, reduction of N_2_O by the dihydride intermediate **B** was examined (Figure 7 and 8).^9–11,15,9–11,41–43^ Hydride transfer from **B** to N_2_O was found to take place via **TS**_**B→A**_ with an energy barrier of 16.0 kcal/mol, leading to the slightly exergonic formation of HNNO^–^ and the concomitant regeneration of **A**. This is followed by the energetically favorable coordination of HNNO^–^ through the oxygen atom to the Ru center to render the anionic complex **C**. The net energy return for the conversion of **B** to **C** is 5.5 kcal/mol. Subsequent N_2_ extrusion from **C** was calculated to take place through a relatively high energy transition state (**TS**_**C→D**_, Δ*G* ^‡^ = 31.5 kcal/mol), leading to the formation of the hydroxo complex **D** after the concerted transfer of the HNNO^–^ hydrogen to the oxygen with simultaneous N_2_ release. However, it was determined that the energy barrier is significantly reduced when the process is facilitated by the involvement of a single water molecule, having the associated **TS′**_**C→D**_ an energy barrier of 15.6 kcal/mol.^11,43^ The formation of the ruthenium hydroxo derivative **D** is highly exergonic by 53.3 kcal/mol. A comparative analysis of the two steps involved in the reduction of N_2_O to N_2_ (**TS**_**A→B**_ and **TS′**_**C→D**_) catalyzed by the Ru–CNC* complex with analogous steps computed for other Ru-based catalysts reveals for the former system the presence of lower energy barriers. Specifically, for Milstein’s Ru–PNP catalyst,^9^ the energy barriers for the hydride transfer to N_2_O and the water-assisted N_2_ elimination step were computed by Poater et al. to be 26.7 and 28.0 kcal/mol, respectively. In contrast, Wu, Xie et al. calculated barriers of 21.8 and 16.0 kcal/mol, respectively, for the same steps.^43^

**Figure 8:**
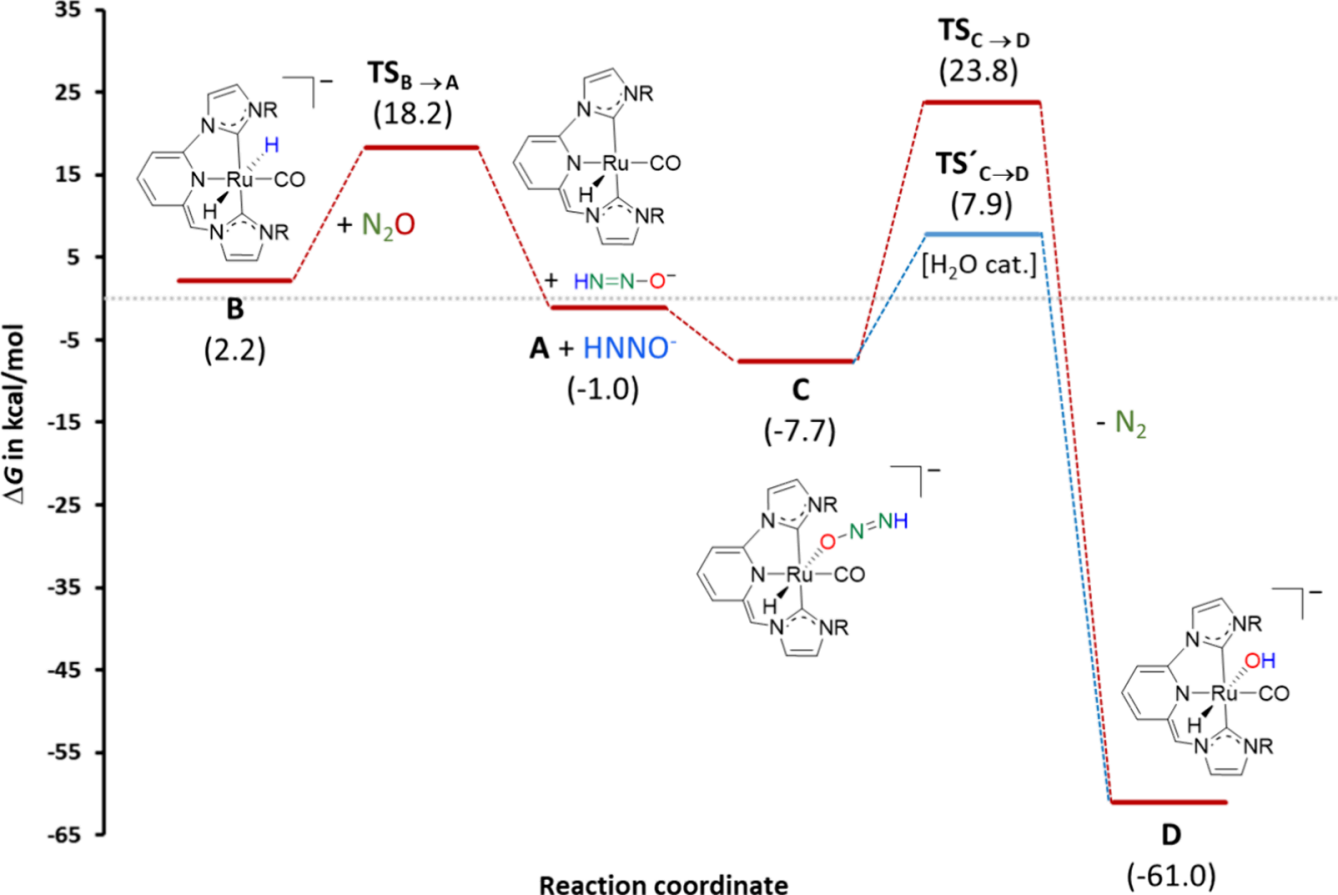
Free energy (Δ*G* in toluene, kcal/mol) profile calculated by DFT for N_2_O reduction by complex **B** (the origin of energies is **6** + EtO^–^ + 2 N_2_O).

Following formation of **D**, two divergent pathways can take place (Figure 7 and 9). First, the nucleophilic attack of the hydroxo ligand to acetaldehyde, which is endergonic (ΔΔ*G* = 5.9 kcal/mol) and exhibits a barrier of 6.1 kcal/mol (**TS**_**D→A**_), produces the formation of 1-hydroxyethanolate and the regeneration of the unsaturated complex **A**. Subsequent, dehydrogenation of 1-hydroxyethanolate by **A** to regenerate **B** and yield acetic acid is highly favored (ΔΔ*G* = – 18.0 kcal/mol) and has a low barrier of only 2.9 kcal/mol. A second process involving complex **D** is the protonation of the hydroxyl ligand by acetic acid that initially produces an “acetate-stabilized aquo complex” (**E**). This last process is barrierless and thermodynamically favorable. Finally, subsequent decomposition of **E** yields acetate and water, and regenerates the unsaturated catalytic species **A.**

**Figure 9:**
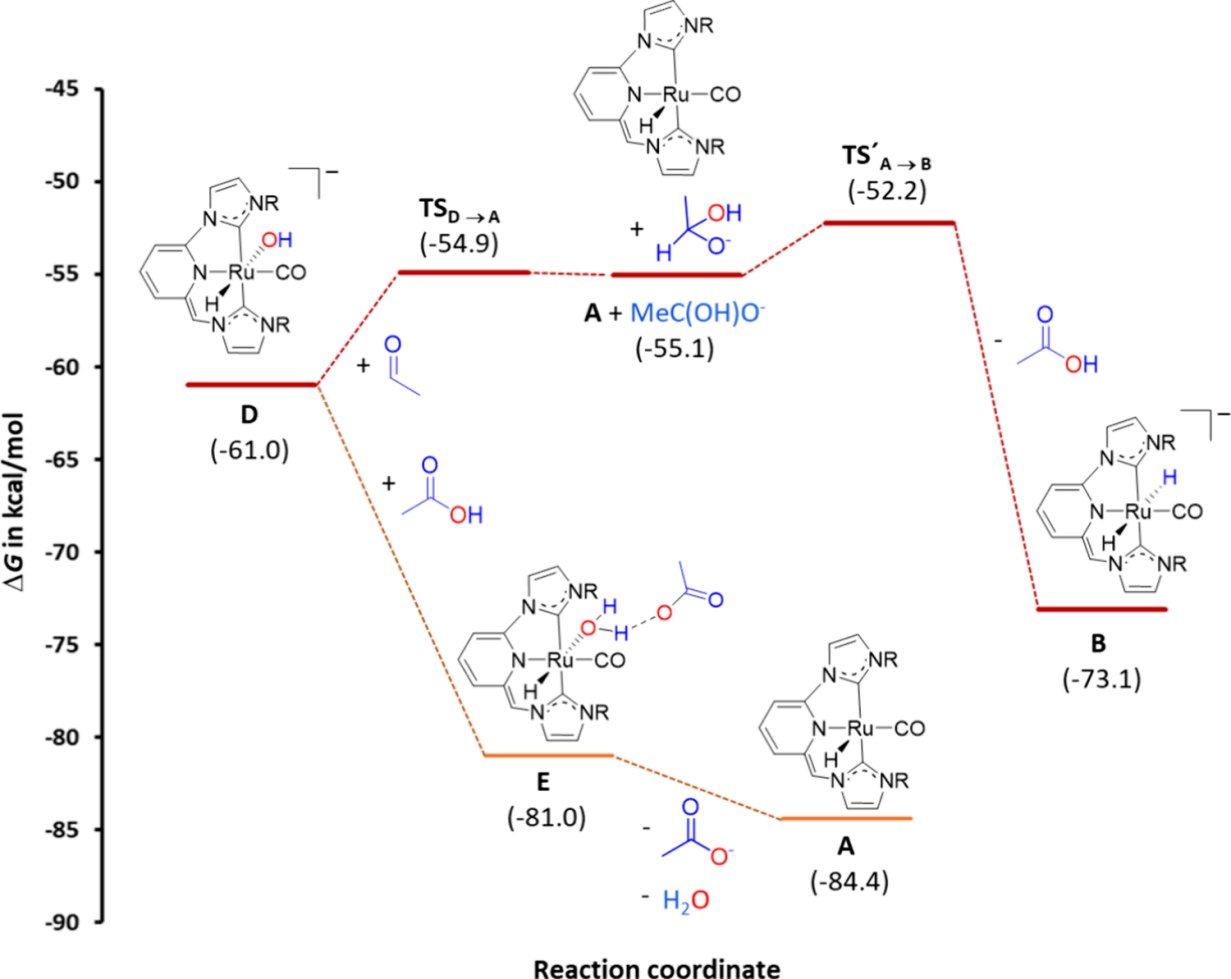
Free energy (Δ*G* in toluene, kcal/mol) profile calculated by DFT for the nucleophilic attack of Ru–OH (**D**) to acetaldehyde, 1-hydroxyethanolate dehydrogenation and acetic acid deprotonation by **D** (the origin of energies is **6** + EtO^–^ + 2 N_2_O).

## Conclusions

In sum, new air-stable ruthenium complexes **3** and **4** incorporating a picoline-derived, proton-responsive CNC ligand are efficient catalysts under basic conditions for the oxidation of alcohols to carboxylates with N_2_O, a relevant greenhouse and ozone-depleting gas. These derivatives readily react with *t*BuOK to yield the deprotonated Ru­(II)-CNC* complex **6** (from **3**) or a Ru(0)-CNC derivative **7** (from **4**). Insights obtained from control experiments and DFT calculations point out to an outer-sphere Ru­(II) based mechanism for the N_2_O oxidation of alcohols. This overall catalytic process comprises several steps: (i) the Ru-catalyzed alcohol dehydrogenation to form a Ru hydride complex and an aldehyde, (ii) the nucleophilic attack of the Ru–H species to N_2_O, leading to the production of N_2_ and a Ru hydroxy intermediate, and (iii) the oxidation of the intermediate aldehyde by the Ru hydroxo complex, resulting in the formation of an alcoholate, which can subsequently undergo dehydrogenation to regenerate the Ru–H species.

Overall, the results herein reported show a convenient catalytic system for N_2_O reduction/alcohol oxidation, and provide a complete landscape of the main reaction mechanism steps. Current investigations in our laboratory is directed to the development of novel N_2_O reduction processes using other widely available hydrogen sources.

## Experimental Section

### General Procedures

All reactions and manipulations were conducted under an inert atmosphere of N_2_ or Ar, utilizing either a Braun Unilab Plus glovebox or standard Schlenk techniques. Solvents were purified by distillation under inert atmosphere employing appropriate drying agents as follows: sodium-benzophenone-ketyl for 2-methyltetrahydrofuran (2Me-THF), tetrahydrofuran (THF, THF-*d*_8_) and diethyl ether (Et_2_O); sodium for toluene and pentane; CaH_2_ for acetonitrile (CH_3_CN) and dichloromethane (CH_2_Cl_2_); and NaOMe for methanol (MeOH). Imidazolium salt **1** was synthesized following a previously reported procedure.^15^ The complex RuHCl­(CO)­(PPh_3_)_3_ was prepared according to established literature methods.^44^ All other reagents were obtained from commercial suppliers and utilized as provided. NMR spectra were obtained on Bruker AVANCE NEO-500, AVANCE III-400R, AVANCE NEO-400 and AVANCE NEO-300 spectrometers. ^13^C­{^1^H} and ^1^H shifts, and ^31^P­{^1^H} shifts, were referenced to the residual signals of deuterated solvents and external 85% H_3_PO_4_, respectively, and are reported in ppm downfield from Me_4_Si. Assignations of NMR resonances were confirmed by two-dimensional NMR spectroscopy (^1^H–^1^H correlated spectroscopy, COSY; ^1^H–^1^H nuclear Overhauser effect spectroscopy, NOESY; ^1^H–^13^C heteronuclear single quantum coherence, HSQC; ^1^H–^13^C heteronuclear multiple bond correlation, HMBC) for all of the complexes. GC-TCD analyses were performed on an Agilent 7820A apparatus equipped with a Carboxen 1010 Plot capillary column (30 m × 0.53 mm). High-resolution mass spectrometry (HRMS) data were acquired using a JEOL JMS-SX 102A mass spectrometer at the Instrumental Services of Universidad de Sevilla (CITIUS). Elemental analyses were performed by the Analytical Service of the Instituto de Investigaciones Químicas utilizing a Leco TruSpec CNH elemental analyzer. Infrared (IR) spectra were recorded on a Bruker Tensor 27 spectrometer.

### Synthesis of CNC Ligand Precursors

#### Imidazolium Salt 2

A mixture of the imidazolium salt **1** (0.240 g, 0.55 mmol) and 1-mesityl-1*H*-imidazole (0.358 g, 1.92 mmol) was dissolved in a 3:1 mixture of THF/MeCN (40 mL). The reaction was stirred for 4 d, after which the resulting precipitate was filtered and washed with tetrahydrofuran (3 × 10 mL). Solid white (0.280 g, 83%). ^1^H NMR (500 MHz, CD_2_Cl_2_): δ 11.69 (dd, ^4^ *J*_HH_ = 1.7 Hz, ^4^ *J*_HH_ = 1.7 Hz, 1H, H arom Imid), 11.15 (dd, ^4^ *J*_HH_ = 1.7 Hz, ^4^ *J*_HH_ = 1.7 Hz, 1H, H arom Imid), 9.18 (dd, ^3^ *J*_HH_ = 1.7 Hz, ^4^ *J*_HH_ = 1.7 Hz, 1H, H arom Imid), 8.64 (m, 1H, H arom Py), 8.63 (m, 1H, H arom Imid), 8.22 (d, ^3^ *J*_HH_ = 7.8 Hz, 1H, H arom Py), 8.07 (dd, ^3^ *J*_HH_ = 7.8 Hz, ^3^ *J*_HH_ = 7.8 Hz, 1H, H arom Py), 7.40 (dd, ^3^ *J*_HH_ = 1.7 Hz, ^4^ *J*_HH_ = 1.7 Hz, 1H, H arom Imid), 7.18 (dd, ^3^ *J*_HH_ = 1.7 Hz, ^4^ *J*_HH_ = 1.7 Hz, 1H, H arom Imid), 7.11 (s, 2H, 2 H arom Mes), 7.04 (s, 2H, 2 H arom Mes), 6.17 (s, 2H, CH_2_N), 2.39 (s, 3H, CH_3_), 2.36 (s, 3H, CH_3_), 2.18 (s, 6H, 2 CH_3_), 2.04 (s, 6H, 2 CH_3_). ^13^C­{^1^H} NMR (126 MHz, CD_2_Cl_2_): δ 153.9 (C_q_ arom), 146.7 (C_q_ arom), 142.5 (CH arom), 141.9 (C_q_ arom), 141.6 (C_q_ arom), 139.1 (CH arom), 137.0 (CH arom), 134.7 (4 C_q_ arom), 131.4 (C_q_ arom), 131.3 (C_q_ arom), 130.2 (2 CH arom), 130.2 (2 CH arom), 126.1 (CH arom), 125.2 (CH arom), 125.2 (CH arom), 123.7 (CH arom), 121.8 (CH arom), 116.2 (CH arom), 53.0 (CH_2_N), 21.4 (CH_3_), 21.4 (CH_3_), 18.2 (2 CH_3_), 18.0 (2 CH_3_). HRMS (ESI): *m*/*z* calcd for C_30_H_32_N_5_ [(*M*–HBr–Br)^+^]: 462.2658; found: 462.2650.

#### Silver Complex **3**

In the absence of light, a solution of **2** (0.283 g, 0.45 mmol) in CH_2_Cl_2_ (15 mL) was treated with Ag_2_O (0.168 g, 0.73 mmol). The resulting mixture was stirred for 24 h and subsequently filtered. The solvent was removed under reduced pressure, and the resulting solid was washed with Et_2_O (3 × 15 mL). White solid (0.313 g, 82%). Anal. Calcd (%) for C_30_H_32_Ag_2_Br_2_N_5_: C 42.99, H 3.85, N 8.36; found: C 43.00, H 3.78, N 8.11. ^1^H NMR (400 MHz, CD_2_Cl_2_): δ 8.02 (d, ^3^ *J*_HH_ = 1.9 Hz, 1H, H arom NHC), 7.97 (dd, ^3^ *J*_HH_ = 7.9 Hz, ^3^ *J*_HH_ = 7.9 Hz, 1H, H arom Py), 7.87 (d, ^3^ *J*_HH_ = 7.9 Hz, 1H, H arom Py), 7.66 (d, ^3^ *J*_HH_ = 1.6 Hz, 1H, H arom NHC), 7.38 (d, ^3^ *J*_HH_ = 7.9 Hz, 1H, H arom Py), 7.17 (d, ^3^ *J*_HH_ = 1.9 Hz, 1H, H arom NHC), 7.04 (s, 2H, 2 H arom Mes), 7.02 (d, ^3^ *J*_HH_ = 1.6 Hz, 1H, H arom NHC), 6.99 (s, 2H, 2 H arom Mes), 5.61 (s, 1H, CH_2_N), 2.37 (s, 3H, CH_3_), 2.35 (s, 3H, CH_3_), 2.05 (s, 6H, 2 CH_3_), 1.96 (s, 6H, 2 CH_3_). ^13^C­{^1^H} NMR (101 MHz, CD_2_Cl_2_): δ 184.5 (C2-NHC), 184.2 (C2-NHC), 156.6 (C_q_ arom), 151.1 (C_q_ arom), 141.7 (CH arom Py), 140.6 (C_q_ arom), 140.4 (C_q_ arom), 136.6 (C_q_ arom), 136.3 (C_q_ arom), 135.6 (2 C_q_ arom), 135.5 (2 C_q_ arom), 130.2 (2 CH arom Mes), 130.0 (2 CH arom Mes), 124.6 (CH arom NHC), 123.8 (CH arom NHC), 123.7 (CH arom NHC), 122.8 (CH arom Py), 120.5 (CH arom NHC), 115.2 (CH arom Py), 56.8 (CH_2_N), 21.7 (CH_3_), 21.6 (CH_3_), 18.4 (2 CH_3_), 18.3 (2 CH_3_).

### Synthesis of Ru-CNC Complexes **4**–**7**

#### Complex **4**

A mixture of the silver complex **3** (0.254 g, 0.30 mmol) and RuHCl­(CO)­(PPh_3_)_3_ (0.295 g, 0.30 mmol) in THF (10 mL) was heated at 60 °C for 24 h in the absence of light. The mixture was filtered and brought to dryness. The solid was washed with Et_2_O (3 × 15 mL) and extracted with MeOH (2 × 10 mL). After evaporation of the solvent, the residue was washed with cold toluene (3 × 10 mL). The solid was then dissolved in MeOH (10 mL) and treated with KBr (0.714 g, 6.0 mmol) for 24 h. Following solvent removal under reduced pressure, the product was extracted with CH_2_Cl_2_ (2 × 5 mL), affording a yellow solid (0.245 g, 86% yield). Crystals suitable for X-ray diffraction analysis were obtained by slow diffusion of toluene into a CH_2_Cl_2_ solution of the complex prior to KBr treatment. Anal. Calcd (%) for C_49_H_47_BrN_5_OPRu: C 63.02, H 5.07, N 7.50; found: C 63.23, H 5.26, N 7.11. ^1^H NMR (400 MHz, CD_2_Cl_2_): δ 8.43 (d, ^3^ *J*_HH_ = 1.2 Hz, 1H, H arom NHC), 7.97 (d, ^3^ *J*_HH_ = 7.6 Hz, 1H, H arom Py), 7.89 (d, ^3^ *J*_HH_ = 1.2 Hz, 1H, H arom NHC), 7.85 (dd, ^3^ *J*_HH_ = 7.6 Hz, ^3^ *J*_HH_ = 7.6 Hz, 1H, H arom Py), 7.50 (d, ^3^ *J*_HH_ = 7.6 Hz, 1H, H arom Py), 7.28 (t, ^3^ *J*_HH_ = 7.3 Hz, 3H, 3 H arom PPh_3_), 7.10 (dd, ^3^ *J*_HH_ = 7.3 Hz, ^3^ *J*_HH_ = 7.3 Hz, 6H, 6 H arom PPh_3_), 6.95 (m, 2H, H arom NHC + H arom Mes), 6.90 (m, 8H, 6 H arom PPh_3_ + 2 H arom Mes), 6.84 (s, 1H, H arom NHC), 6.77 (s, 1H, H arom Mes), 5.89 (d, ^2^ *J*_HH_ = 15.7 Hz, 1H, C*H*HN), 4.26 (d, ^2^ *J*_HH_ = 15.7 Hz, 1H, CH*H*N), 2.35 (s, 3H, CH_3_), 2.34 (s, 3H, CH_3_), 2.08 (s, 3H, CH_3_), 2.02 (s, 3H, CH_3_), 1.84 (s, 3H, CH_3_), 1.22 (s, 3H, CH_3_), –8.49 (d, ^2^ *J*_HP_ = 88.2 Hz, 1H, RuH). ^13^C­{^1^H} NMR (101 MHz, CD_2_Cl_2_): δ 205.6 (d, *J*_CP_ = 8 Hz, CO), 200.0 (d, *J*_CP_ = 6 Hz, C2-NHC), 188.6 (d, *J*_CP_ = 8 Hz, C2-NHC), 154.6 (C_q_ arom), 153.2 (C_q_ arom), 140.7 (CH arom), 139.9 (C_q_ arom), 139.8 (C_q_ arom), 137.9 (C_q_ arom), 137.3 (2 C_q_ arom), 136.7 (C_q_ arom), 136.3 (d, *J*_CP_ = 30 Hz, 3 C_q_ arom PPh_3_), 136.1 (C_q_ arom), 135.6 (C_q_ arom), 133.0 (d, *J*_CP_ = 12 Hz, 6 CH arom PPh_3_), 130.4 (3 CH arom PPh_3_), 130.1 (CH arom), 129.8 (CH arom), 129.5 (CH arom),129.4 (CH arom), 129.1 (d, *J*_CP_ = 9 Hz, 6 CH arom PPh_3_), 128.8 (CH arom), 123.9 (CH arom), 123.3 (CH arom), 121.6 (CH arom), 117.7 (CH arom), 111.9 (CH arom), 54.5 (overlapped with solvent signal, CH_2_N), 21.6 (CH_3_), 21.5 (CH_3_), 19.4 (CH_3_), 19.2 (CH_3_), 18.8 (CH_3_), 17.6 (CH_3_). ^31^P­{^1^H} NMR (162 MHz, CD_2_Cl_2_): δ 28.2. IR (CH_2_Cl_2_): 1951 (ν_CO_) cm^–1^.

#### Complex **5**

A suspension of complex **4** (0.049 g, 0.05 mmol) in MeCN (5 mL) was treated with NaBF_4_ (0.007 g, 0.06 mmol) and stirred at room temperature for 24 h. The solvent was removed under reduced pressure, and the residue was extracted with CH_2_Cl_2_ (2 × 5 mL). The combined organic extracts were evaporated to dryness, and the residue was dissolved in THF (5 mL). The resulting solution was transferred to a Fisher–Porter reactor and pressurized with CO (3 bar) at room temperature for 24 h. After careful release of the pressure, the solution was filtered and concentrated to dryness. The obtained solid was washed with cold toluene (3 × 5 mL) to afford complex **5** as a yellow solid (0.028 g, 79%). Anal. Calcd (%) for C_32_H_32_BF_4_N_5_O_2_Ru: C 54.40, H 4.57, N 9.91; found C 54.27, H 4.22, N 9.74. ^1^H NMR (400 MHz, THF-*d*_8_): δ 8.61 (d, ^3^ *J*_HH_ = 2.2 Hz, 1H, H arom NHC), 8.30 (m, 2H, 2 H arom Py), 8.06 (d, ^3^ *J*_HH_ = 2.0 Hz, 1H, H arom NHC), 8.04 (m, 1H, H arom Py), 7.42 (d, ^3^ *J*_HH_ = 2.2 Hz, 1H, H arom NHC), 7.14 (d, ^3^ *J*_HH_ = 2.0 Hz, 1H, H arom NHC), 7.03 (s, 1H, H arom Mes), 6.94 (m, 2H, 2 H arom Mes), 6.89 (s, 1H, H arom Mes), 6.30 (d, ^2^ *J*_HH_ = 15.5 Hz, 1H, C*H*HN), 5.16 (d, ^2^ *J*_HH_ = 15.5 Hz, 1H, CH*H*N), 2.30 (s, 3H, CH_3_), 2.28 (s, 3H, CH_3_), 2.11 (s, 3H, CH_3_), 2.02 (s, 3H, CH_3_), 1.95 (s, 3H, CH_3_), 1.92 (s, 3H, CH_3_), –6.28 (s, 1H, RuH). ^13^C­{^1^H} NMR (101 MHz, THF-*d*_8_): δ 200.1 (CO), 194.3 (CO), 193.8 (C2-NHC), 181.7 (C2-NHC), 156.3 (C_q_ arom), 155.0 (C_q_ arom), 143.8 (CH arom), 141.3 (C_q_ arom), 140.9 (C_q_ arom), 137.9 (C_q_ arom), 137.5 (C_q_ arom), 137.5 (C_q_ arom), 137.3 (C_q_ arom), 136.9 (C_q_ arom), 136.6 (C_q_ arom), 130.8 (CH arom), 130.7 (CH arom), 130.5 (2 CH arom), 126.6 (CH arom), 125.8 (CH arom), 124.0 (CH arom), 123.8 (CH arom), 119.6 (CH arom), 113.6 (CH arom), 56.0 (CH_2_N), 21.9 (2 CH_3_), 19.2 (CH_3_), 18.5 (CH_3_), 18.4 (CH_3_), 18.4 (CH_3_). IR (CH_2_Cl_2_): 2036 (ν_CO_), 1985 (ν_CO_) cm^–1^.

#### Complex **6**

In a J.Young-valved NMR tube, a solution of complex **4** (0.015 g, 0.01 mmol) in THF-*d*_8_ (0.5 mL) was treated with KHMDS (0.004 g, 0.02 mmol), rendering the immediate formation of a dark-red solution. After 30 min, the reaction mixture was analyzed by NMR spectroscopy, revealing the quantitative formation of complex **6** (Figure 10). Attempts to isolate the product were unsuccessful due to its pronounced sensitivity. However, crystals appropriate for X-ray crystallographic analysis were grown by the slow evaporation of a saturated solution of the complex in THF.

**Figure 10:**
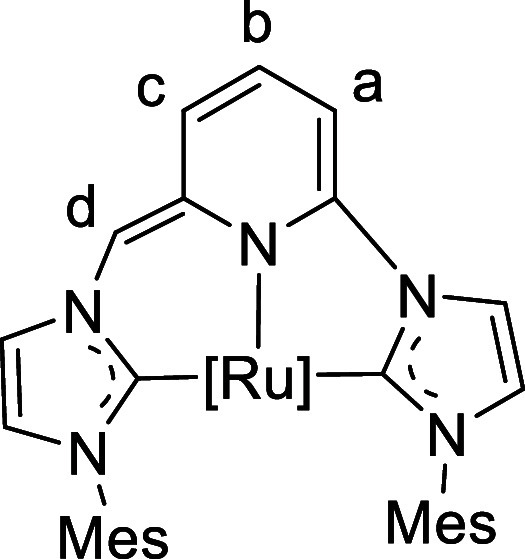
Labeling for NMR signal assignment of complex **6.**

^1^H NMR (500 MHz, THF-*d*_8_): δ 7.59 (d, ^3^ *J*_HH_ = 2.1 Hz, 1H, H arom NHC), 7.20 (t, ^3^ *J*_HH_ = 7.5 Hz, 3H, 3 H arom PPh_3_), 7.12 (d, ^3^ *J*_HH_ = 1.7 Hz, 1H, H arom NHC), 7.06 (dd, ^3^ *J*_HH_ = 7.5 Hz, ^3^ *J*_HH_ = 7.5 Hz, 6H, 6 H arom PPh_3_), 6.94 (dd, ^3^ *J*_HH_ = 7.5 Hz, ^3^ *J*_HP_ = 7.5 Hz, 6H, 6 H arom PPh_3_), 6.83 (s, 1H, H arom Mes), 6.80 (s, 1H, H arom Mes), 6.79 (d, ^3^ *J*_HH_ = 2.1 Hz, 1H, H arom NHC), 6.74 (s, 1H, H arom Mes), 6.63 (d, ^3^ *J*_HH_ = 1.7 Hz, 1H, H arom NHC), 6.61 (s, 1H, H arom Mes), 6.18 (dd, ^3^ *J*_HH_ = 8.9 Hz, ^3^ *J*_HH_ = 6.6 Hz, 1H, H^b^), 5.55 (s, 1H, H^d^), 5.47 (d, ^3^ *J*_HH_ = 8.9 Hz, 1H, H^c^), 5.38 (d, ^3^ *J*_HH_ = 6.6 Hz, 1H, H^a^), 2.29 (s, 3H, CH_3_), 2.22 (s, 3H, CH_3_), 2.18 (s, 3H, CH_3_), 1.96 (s, 3H, CH_3_), 1.27 (s, 3H, CH_3_), 1.20 (s, 3H, CH_3_), –8.38 (d, ^3^ *J*_HP_ = 121.5 Hz, 1H, RuH). ^13^C­{^1^H} NMR (126 MHz, THF-*d*_8_): δ 207.9 (CO), 200.3 (d, *J*_CP_ = 7 Hz, C2-NHC), 182.2 (d, *J*_CP_ = 9 Hz, C2-NHC), 151.9 (C_q_ arom), 143.6 (C_q_ arom), 139.8 (C_q_ arom), 139.5 (C_q_ arom), 139.3 (C_q_ arom), 139.1 (C_q_ arom), 138.8 (d, *J*_CP_ = 36 Hz, 3 C_q_ arom PPh_3_), 138.5 (C_q_ arom), 138.2 (C_q_ arom), 137.9 (C_q_ arom), 136.9 (C_q_ arom), 135.2 (d, *J*_CP_ = 13 Hz, 6 CH arom PPh_3_), 130.3 (CH arom), 130.0 (CH arom), 129.9 (CH arom), 129.8 (CH arom), 129.7 (3 CH arom PPh_3_), 129.1 (d, *J*_CP_ = 7 Hz, 6 CH arom PPh_3_), 128.6 (C^b^), 124.1 (CH arom), 122.0 (CH arom), 120.4 (CH arom), 116.5 (CH arom), 112.6 (C^c^), 91.5 (C^d^), 86.0 (C^a^), 22.0 (CH_3_), 22.0 (CH_3_), 20.0 (CH_3_), 19.8 (CH_3_), 18.7 (CH_3_), 18.3 (CH_3_). ^31^P­{^1^H} NMR (202 MHz, THF-*d*_8_): δ 26.0. IR (THF): 1921 (ν_CO_) cm^–1^.

#### Complex **7**-iso

In a J.Young-valved NMR tube, a solution of complex **4** (0.015 g, 0.01 mmol) in THF-*d*_8_ (0.5 mL) was treated with KHMDS (0.004 g, 0.02 mmol), resulting in the formation of a dark-red solution. The sample was pressurized with CO (3 bar), which led to a distinct color change from dark-red to blue. After 3 h, NMR spectroscopy analysis revealed the quantitative formation of complex **7-iso**. Attempts to isolate the product were unsuccessful due to its pronounced sensitivity. ^1^H NMR (500 MHz, THF-*d*_8_): δ 8.12 (s, 1H, H arom NHC), 7.67 (d, ^3^ *J*_HH_ = 7.4 Hz, 1H, H arom Py), 7.47 (s, 1H, H arom NHC), 7.43 (dd, ^3^ *J*_HH_ = 7.4 Hz, ^3^ *J*_HH_ = 7.1 Hz, 1H, H arom Py), 7.15 (d, ^3^ *J*_HH_ = 7.1 Hz, 1H, H arom Py), 7.03 (s, 1H, H arom NHC), 6.86 (m, 3H, H arom NHC + 2 H arom Mes), 6.79 (s, 2H, 2 H arom Mes), 5.15 (s, 2H, CH_2_N), 2.28 (s, 3H, CH_3_), 2.25 (s, 3H, CH_3_), 2.07 (s, 6H, 2 CH_3_), 1.79 (s, 6H, 2 CH_3_). ^13^C­{^1^H} NMR (101 MHz, THF-*d*_8_): δ 211.0 (2 CO), 200.2 (C2-NHC), 193.8 (C2-NHC), 185.7 (C_q_ arom), 149.8 (C_q_ arom), 143.6 (C_q_ arom), 138.9 (C_q_ arom), 138.8 (C_q_ arom), 138.7 (C_q_ arom), 138.4 (2 C_q_ arom), 137.4 (2 C_q_ arom), 129.9 (2 CH arom), 129.8 (2 CH arom), 125.7 (CH arom), 124.5 (CH arom), 122.8 (CH arom), 122.0 (CH arom), 114.2 (CH arom), 112.0 (CH arom), 109.4 (CH arom), 57.8 (CH_2_N), 21.8 (CH_3_), 21.8 (CH_3_), 19.1 (2 CH_3_), 18.7 (2 CH_3_).

### Representative Procedure for the Catalytic Oxidation of Heavy Alcohols with N_2_O

In a glovebox, a Fisher-Porter reactor (25 mL) was charged with a solution of **4** (5.6 mg, 6 μmol), *t*BuOK (0.08 g, 0.72 mmol), the corresponding alcohol (0.60 mmol) and mesitylene (70 μL, 0.50 mmol) in toluene (1.0 mL). The N_2_ atmosphere in the vessel was replaced by N_2_O (1.0 bar) by carrying out three freeze–pump–thaw cycles, and the reactor was heated to 120 °C. After 24 h, the reactor was slowly cooled down to room temperature, and the gas atmosphere was analyzed by GC–TCD to detect N_2_ formation. Volatiles of the reaction were evaporated under reduced pressure, and conversion was determined by ^1^H NMR spectroscopy. To further confirm the identity of the resulting carboxylates by the isolation of the corresponding carboxylic acids, the reaction mixture was treated with a 0.5 M aqueous solution of HCl until the pH of the solution was 7. The carboxylic acid was extracted with Et_2_O (3 × 2 mL), and the resulting solution was dried with Na_2_SO_4_, and brought to dryness.

### Representative Procedure for the Catalytic Oxidation of Light Alcohols (Ethanol and 2-Methylethanol) with N_2_O

In a glovebox, a Fisher-Porter reactor (25 mL) was charged with a solution of **4** (5.6 mg, 6.0 μmol), *t*BuOK (0.08 g, 0.72 mmol) and ethanol (35 μL, 0.60 mmol) in toluene (1.0 mL). The N_2_ atmosphere in the vessel was replaced by N_2_O (1.0 bar) by carrying out three freeze–pump–thaw cycles, and the reactor was heated to 120 °C for 24 h. Volatiles of the reaction were evaporated under reduced pressure, and the residue was dissolved in D_2_O (0.6 mL). The yield of sodium acetate was determined by ^1^H NMR spectroscopy using DMSO as internal standard.

## Supplementary Material







## Notes

Cartesian coordinates, absolute Gibbs energies (THF or toluene), and imaginary frequencies (TSs) of the DFT-optimized structures (collective *XYZ* file) (XYZ)
Global CIF with all two structures (CIF)
Selected NMR spectra of complexes and products, X-ray diffraction analysis details and computational details (PDF)

## References

[1] Hansen J., Sato M. (2004). Greenhouse Gas Growth Rates. Proc. Natl. Acad.
Sci. U. S. A..

[2] Prather M. J. (1998). Time Scales in Atmospheric Chemistry:
Coupled Perturbations
to N_2_O, NO_γ_, and O_3_. Science.

[3] Davidson E. A., Kanter D. (2014). Inventories and Scenarios of Nitrous Oxide Emissions. Environ. Res. Lett..

[4] Jabłońska M., Palkovits R. (2016). It Is No Laughing
Matter: Nitrous Oxide Formation in
Diesel Engines and Advances in its Abatement over Rhodium-Based Catalyst. Catal. Sci. Technol..

[5] Kumar A., Gao C. (2021). Homogeneous (De)­hydrogenative
Catalysis
for Circular Chemistry – Using Waste as a Resource. ChemCatChem..

[6] Gorelsky S. I., Ghosh S., Solomon E. I. (2006). Mechanism
of N_2_O Reduction by the μ_4_-S Tetranuclear
Cu_Z._ Cluster of Nitrous Oxide Reductase. J. Am. Chem. Soc..

[7] Tolman W. B. (2010). Binding and Activation of N_2_O at Transition-Metal
Centers: Recent Mechanistic Insights. Angew.
Chem. Int. Ed.

[8] Armor J. N., Taube H. (1969). Formation and Reactions of [(NH_3_)_5_RuN_2_O_2_
^+^]. J. Am. Chem. Soc..

[9] Zeng R., Feller M., Ben-David Y., Milstein D. (2017). Hydrogenation and Hydrosilylation
of Nitrous Oxide Homogeneously Catalyzed by a Metal Complex. J. Am. Chem. Soc..

[10] Jurt P., Abels A. S., Gamboa-Carballo J. J., Fernández I., Le Corre G., Aebli M., Baker M. G., Eiler F., Müller F., Wörle M., Verel R., Gauthier S., Trincado M., Gianetti T. L., Grützmacher H. (2021). Reduction of Nitrogen Oxides by Hydrogen with Rhodium­(I)-Platinum­(II)
Olefin Complexes as Catalysts. Angew. Chem.,
Int. Ed..

[11] Ortega-Lepe I., Sánchez P., Santos L. L., Lara P., Rendón N., López-Serrano J., Salazar-Pereda V., Álvarez E., Paneque M., Suárez A. (2022). Catalytic Nitrous Oxide Reduction
with H_2_ Mediated by Pincer Ir Complexes. Inorg. Chem..

[12] Zeng R., Feller M., Diskin-Posner Y., Shimon L. J. W., Ben-David Y., Milstein D. (2018). CO Oxidation by N_2_O Homogeneously Catalyzed
by Ruthenium Hydride Pincer Complexes Indicating a New Mechanism. J. Am. Chem. Soc..

[13] Gianetti T. L., Rodríguez-Lugo R. E., Harmer J. R., Trincado M., Vogt M., Santiso-Quinones G., Grützmacher H. (2016). Zero-Valent
Amino-Olefin Cobalt Complexes as Catalysts for Oxygen Atom Transfer
Reactions from Nitrous Oxide. Angew. Chem.,
Int. Ed..

[14] Chen X., Wang H., Du S., Driess M., Mo Z. (2022). Deoxygenation of Nitrous Oxide and
Nitro Compounds Using Bis­(N-Heterocyclic Silylene)­Amido Iron Complexes
as Catalysts. Angew. Chem., Int. Ed..

[15] Bermejo J., Ortega-Lepe I., Santos L. L., Rendón N., López-Serrano J., Álvarez E., Suárez A. (2024). Nitrous Oxide
Activation by Picoline-Derived Ni–CNP Hydrides. Chem. Commun..

[16] Molinillo P., Lacroix B., Vattier F., Rendón N., Suárez A., Lara P. (2022). Reduction of N_2_O with
Hydrosilanes Catalysed by RuSNS Nanoparticles. Chem. Commun..

[17] Le Vaillant F., Mateos Calbet A., Gonzalez-Pelayo S., Reijerse E. J., Ni S., Busch J., Cornella J. (2022). Catalytic
Synthesis of Phenols with Nitrous Oxide. Nature.

[18] Kumar A., Bhardwaj R., Mandal S. K., Choudhury J. (2022). Transfer Hydrogenation
of CO_2_ and CO_2_ Derivatives using Alcohols as
Hydride Sources: Boosting an H_2_-Free Alternative Strategy. ACS Catal..

[19] Wang D., Astruc D. (2015). The Golden Age of Transfer Hydrogenation. Chem. Rev..

[20] Wang L. Q., Li T., Ma H. H. (2021). Explosion Behaviors
of Hydrogen-Nitrous Oxide Mixtures
at Reduced Initial Pressures. Process Saf. Environ.
Prot..

[21] Gianetti T. L., Annen S. P., Santiso-Quinones G., Reiher M., Driess M., Grützmacher H. (2016). Nitrous Oxide as a Hydrogen Acceptor for the Dehydrogenative
Coupling of Alcohols. Angew. Chem., Int. Ed..

[22] Bösken J., Rodríguez-Lugo R. E., Nappen S., Trincado M., Grützmacher H. (2023). Reduction
of Nitrous Oxide by Light Alcohols Catalysed
by a Low-Valent Ruthenium Diazadiene Complex. Chem.—Eur. J..

[23] Hashimoto K., Kitaichi Y., Tanaka H., Ikeno T., Yamada T. (2001). Nitrous Oxide Oxidation of Secondary
and Benzylic Alcohols Using Ruthenium Complex Catalyst. Chem. Lett..

[24] Lin I. J. B., Vasam C. S. (2007). Preparation and
Application of N-Heterocyclic Carbene
Complexes of Ag­(I). Coord. Chem. Rev..

[25] Gupta A., Verma J. P. (2015). Sustainable Bio-Ethanol
Production from Agro-Residues:
A Review. Renew. Sustain. Energy Rev..

[26] Chen Y., Yang Y., Liu X., Shi X., Wang C., Zhong H., Jin F. (2023). Sustainable Production
of Formic Acid and Acetic Acid from Biomass. Mol. Catal..

[27] Ohtani B., Takamiya S., Hirai Y., Sudoh M., Nishimoto S., Kagiya T. (1992). Catalytic Oxidation
with Nitrous Oxide: Oxidation of
Alcohols, Ethers and Amines in an Aqueous Suspension of Platinum Particles
at Room Temperature. J. Chem. Soc., Perkin Trans..

[28] Sun Y., Koehler C., Tan R., Annibale V. T., Song D. (2011). Ester Hydrogenation Catalyzed by
Ru-CNN Pincer Complexes. Chem. Commun..

[29] Sánchez P., Hernández-Juárez M., Rendón N., López-Serrano J., Álvarez E., Paneque M., Suárez A. (2018). Hydroboration of Carbon Dioxide with
Catechol and Pinacolborane Using an Ir–CNP* Pincer Complex.
Water Influence on the Catalytic Activity. Dalton
Trans.

[30] Anaby A., Schelwies M., Schwaben J., Rominger F., Hashmi A. S. K., Schaub T. (2018). Study of Precatalyst
Degradation Leading to the Discovery
of a New Ru^0^ Precatalyst for Hydrogenation and Dehydrogenation. Organometallics.

[31] Deolka S., Fayzullin R. R., Khaskin E. (2022). Bulky PNP Ligands Blocking
Metal-Ligand
Cooperation Allow for Isolation of Ru(0), and Lead to Catalytically
Active Ru Complexes in Acceptorless Alcohol Dehydrogenation. Chem.—Eur. J..

[32] Eizawa A., Nishimura S., Arashiba K., Nakajima K., Nishibayashi Y. (2018). Synthesis
of Ruthenium Complexes Bearing PCP-Type Pincer Ligands and Their Application
to Direct Synthesis of Imines from Amines and Benzyl Alcohol. Organometallics.

[33] Sánchez P., Hernández-Juárez M., Rendón N., López-Serrano J., Santos L. L., Álvarez E., Paneque M., Suárez A. (2020). Hydrogenation/Dehydrogenation
of
N-Heterocycles Catalyzed by Ruthenium Complexes based on Multimodal
Proton-Responsive CNN­(H) Pincer Ligands. Dalton
Trans..

[34] Tindall D.
J., Menche M., Schelwies M., Paciello R. A., Schäfer A., Comba P., Rominger F., Hashmi A. S. K., Schaub T. (2020). Ru^0^ or Ru^II^: A Study on Stabilizing the “Activated”
Form of Ru-PNP Complexes with Additional Phosphine Ligands in Alcohol
Dehydrogenation and Ester Hydrogenation. Inorg.
Chem..

[35] He T., Buttner J. C., Reynolds E. F., Pham J., Malek J. C., Keith J. M., Chianese A. R. (2019). Dehydroalkylative
Activation of CNN- and PNN-Pincer Ruthenium Catalysts for Ester Hydrogenation. J. Am. Chem. Soc..

[36] Waluk J. (2024). Nuclear Quantum Effects in Proton
or Hydrogen Transfer. J. Phys. Chem. Lett..

[37] Ben-Ari E., Leitus G., Shimon L. J. W., Milstein D. (2006). Metal-Ligand
Cooperation in C-H and H_2_ Activation
by an Electron-Rich PNP Ir­(I) System: Facile Ligand Dearomatization-Aromatization
as Key Steps. J. Am. Chem. Soc..

[38] Zeng G., Guo Y., Li S. (2009). H_2_ Activation by a (PNP)­Ir­(C_6_H_5_) Complex via
the Dearomatization/Aromatization Process of the PNP Ligand: A Computational
Study. Inorg. Chem..

[39] Aitchison H., Wingad R. L., Wass D. F. (2016). Homogeneous Ethanol
to Butanol Catalysis—Guerbet Renewed. ACS Catal..

[40] Kar S., Milstein D. (2022). Oxidation of Organic Compounds Using Water as the Oxidant
with H_2_ Liberation Catalyzed by Molecular Metal Complexes. Acc. Chem. Res..

[41] Kaplan A. W., Bergman R. G. (1997). Nitrous Oxide Mediated
Oxygen Atom Insertion into a Ruthenium-Hydride Bond. Synthesis and
Reactivity of the Monomeric Hydroxoruthenium Complex (DMPE)­2Ru­(H)­(OH). Organometallics.

[42] Yu H., Jia G., Lin Z. (2008). Theoretical
Studies on O-Insertion Reactions of Nitrous Oxide with Ruthenium Hydride
Complexes. Organometallics.

[43] Luque-Urrutia J. A., Poater A. (2017). The Fundamental Noninnocent
Role of Water for the Hydrogenation of Nitrous Oxide by Pincer Ru-Based
Catalysts. Inorg. Chem..

[44] Ahmad N., Levison J. J., Robinson S. D., Uttley M. F., Wonchoba E. R., Parshall G. W. (1974). Complexes of Ruthenium, Osmium, Rhodium,
and Iridium
Containing Hydride Carbonyl, or Nitrosyl Ligands. Inorg. Synth..

