# Supplementary material for: Oxidation of Alcohols to Carboxylates with N2O Catalyzed by Ruthenium(II)-CNC Complexes
Source: ACS Catal. 2025 Jun 20;15(13):11530–43. doi: 10.1021/acscatal.5c02021 (PMC12235671; doi:10.1021/acscatal.5c02021)
Supplement: Supplementary file 3 [file cs5c02021_si_003.pdf]

Supporting Information

Oxidation of Alcohols to Carboxylates with N_2O Catalyzed by Ruthenium(II)-CNC Complexes

José Bermejo,[a] *Laura L. Santos,*[a,*] *Eleuterio Álvarez,*[b] *Joaquín López-Serrano,*[a] *and Andrés Suárez*[a,*]

[a] Instituto de Investigaciones Químicas (IIQ) and Centro de Innovación en Química Avanzada (ORFEO-CINQA). CSIC and Universidad de Sevilla. Avda Américo Vespucio, 49; 41092, Sevilla, Spain.

[b] Instituto de Investigaciones Químicas (IIQ). CSIC and Universidad de Sevilla. Avda Américo Vespucio, 49; 41092, Sevilla, Spain.

E-mail: laura@iiq.csic.es

E-mail: andres.suarez@iiq.csic.es

Table of Contents

1. Catalytic Control Experiments..3

2. NMR Spectra of Compounds **2-6** and **7-iso**..8

3. NMR Spectra of Catalysis Products……….……………………………………….…………..……17

4. X-Ray Structural Analysis of Complexes **4** and **6**……………………………………..……...29

5. DFT Calculations………………………………………………………………………….………34

1. Catalytic Control Experiments

1.1. Catalytic reaction in the presence of Hg.

In a glovebox, a Fisher-Porter vessel (25 mL) was charged with a solution of complex **4** (5.6 mg, 6.0 µmol), *t*BuOK (0.08 g, 0.72 mmol), 1-hexanol (75 µL, 0.60 mmol) and mesitylene (70 µL, 0.50 mmol) in toluene (1.0 mL) and Hg (0.086 g, 0.40 mmol). The nitrogen atmosphere in the reactor was replaced by 1.0 bar of N_2O by performing three freeze−pump−thaw cycles, and the vessel was heated to 120 °C. After 24 h, volatiles of the reaction were evaporated under reduced pressure, and conversion was determined by ^{1}H NMR spectroscopy (96% NMR yield of carboxylate).

1.2. Alcohol dehydrogenation in the absence of N_2O.

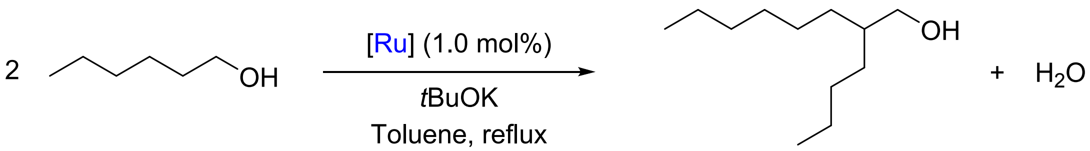

32%

In a glovebox, a Fisher-Porter vessel (25 mL) was charged with a solution of complex **4** (5.6 mg, 6.0 µmol), *t*BuOK (0.08 g, 0.72 mmol), 1-hexanol (75 µL, 0.60 mmol) and mesitylene (70 µL, 0.50 mmol) in toluene (1.0 mL). The vessel was heated to 120 °C and, after 24 h, volatiles of the reaction were evaporated under reduced pressure and conversion was determined by ^{1}H NMR spectroscopy (32% NMR yield).

1.3. Alcohol dehydrogenation in the presence of N_2O and 4 Å molecular sieves.

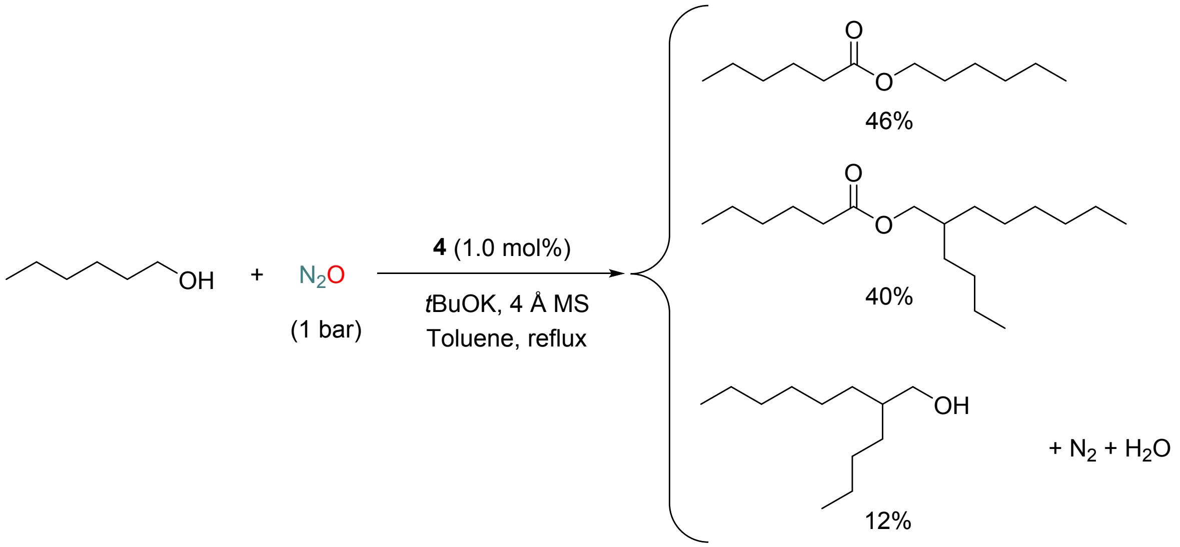

In a glovebox, a Fisher-Porter vessel (25 mL) was charged with a solution of complex **4** (5.6 mg, 6.0 μmol), *t*BuOK (0.08 g, 0.72 mmol), 1-hexanol (75 μL, 0.60 mmol) and mesitylene (70 μL, 0.50 mmol) in toluene (1.0 mL) and 4 Å molecular sieves (0.750 g). The nitrogen atmosphere in the reactor was replaced by 1.0 bar of N_2O by performing three freeze−pump−thaw cycles, and the vessel was heated to 120 °C for 24 h. The volatiles of the reaction were evaporated under reduced pressure, and conversion was determined by ^{1}H NMR spectroscopy (98% hexanol conversion).

1.4. Hydrogenation of N_2O.

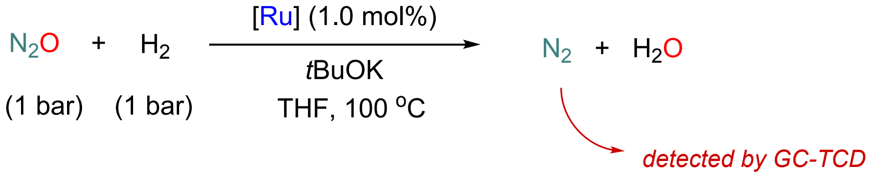

In a glovebox, a Fisher–Porter vessel (25 mL) was charged with a solution of complex **4** (1.5 mg, 1.6 μmol), *t*BuOK (1.3 mg, 0.16 mmol) and mesitylene (5.0 μL, 36 μmol) in THF (0.6 mL).

The nitrogen atmosphere in the reactor was replaced by 1.0 bar of H_2 by performing three freeze-pump-thaw cycles, and the vessel was further pressurized with N_2O until a total gauge pressure of 2.0 bar (N_2O/H_2 ratio = 1:1) and heated to 100 °C. After 24 h, the gas atmosphere was analyzed by GC-TCD to detect N_2 formation (Figure S2). The reactor was depressurized, and the solution was transferred under inert atmosphere to a NMR tube containing a coaxial insert filled with C_6D_6. Conversion was determined through ^{1}H NMR spectroscopy by integrating the H_2O signal using mesitylene as internal standard (Figure S1).[1] (TON = 750; TOF = 32.6 h^{-1})

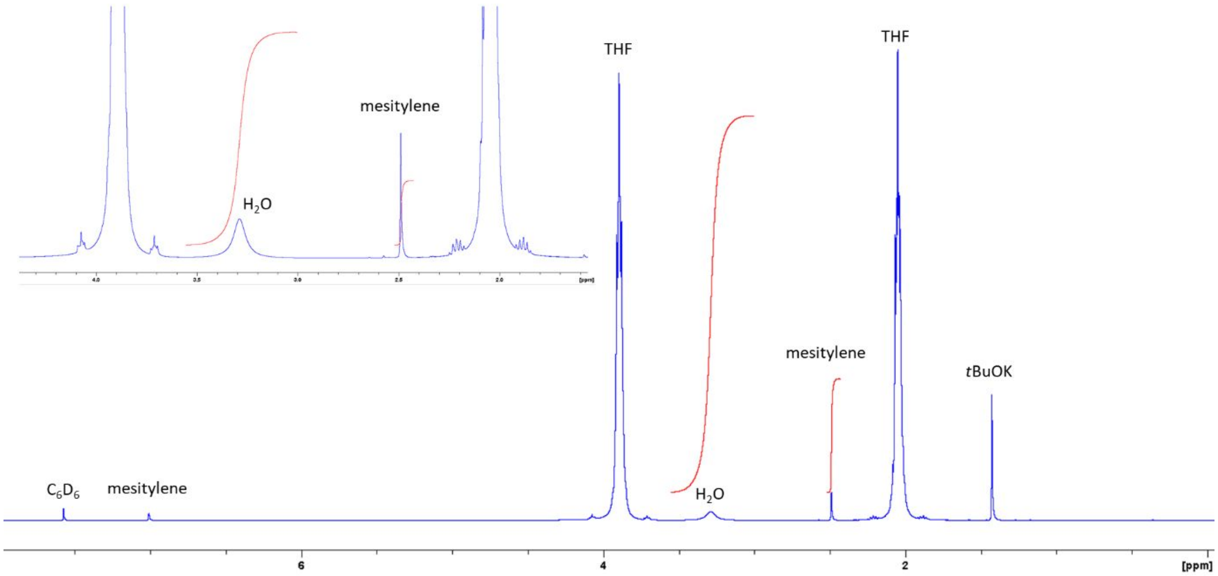

Figure S1. ^{1}H NMR spectrum of the hydrogenation reaction of N_2O (2 bar; N_2O/H_2 = 1:1) catalyzed by **4** + *t*BuOK (400 MHz, THF, C_6D_6 insert). [H_2O signal chemical shift varies with concentration]

[1] I. Ortega-Lepe, P. Sánchez, L. L. Santos, P. Lara, N. Rendón, J. López-Serrano, V. Salazar-Pereda, E. Álvarez, M. Paneque, A. Suárez. *Inorg. Chem.* **2022**, *61*, 18590–18600.

1.5. Oxidation of benzaldehyde with water.

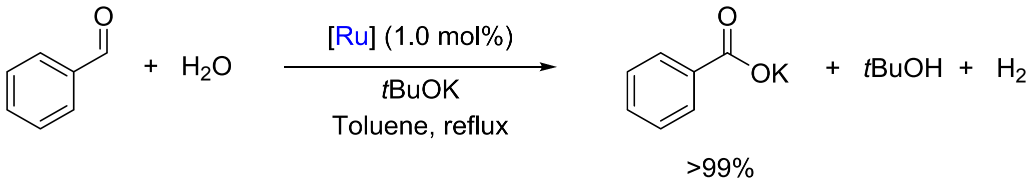

In a glovebox, a Fisher-Porter vessel (25 mL) was charged with a solution of complex **4** (5.6 mg, 6.0 μmol), *t*BuOK (0.08 g, 0.72 mmol), benzaldehyde (61 μL, 0.60 mmol), H_2O (160 μL, 8.8 mmol) and mesitylene (70 μL, 0.50 mmol) in toluene (1.0 mL). The vessel was heated to 120 °C for 24 h. Volatiles were evaporated under reduced pressure, and conversion was determined by ^1H NMR spectroscopy (>99 % NMR yield).

1.6. N_2 detection by GC-TCD.

GC-TCD analysis were carried out using an Agilent 7820A apparatus equipped with a Carboxen 1010 Plot (30 m × 0.53 mm) column. Argon carrier gas was supplied at a head pressure of 2.4 psi to provide an initial flow rate of 2.5 mL/min. The injector temperature was setup to 230 ^{o}C, and the oven temperature was initially held at 30 ^{o}C for 7 min, then gradually increased to 220 ^{o}C at 25 ^{o}C/min.

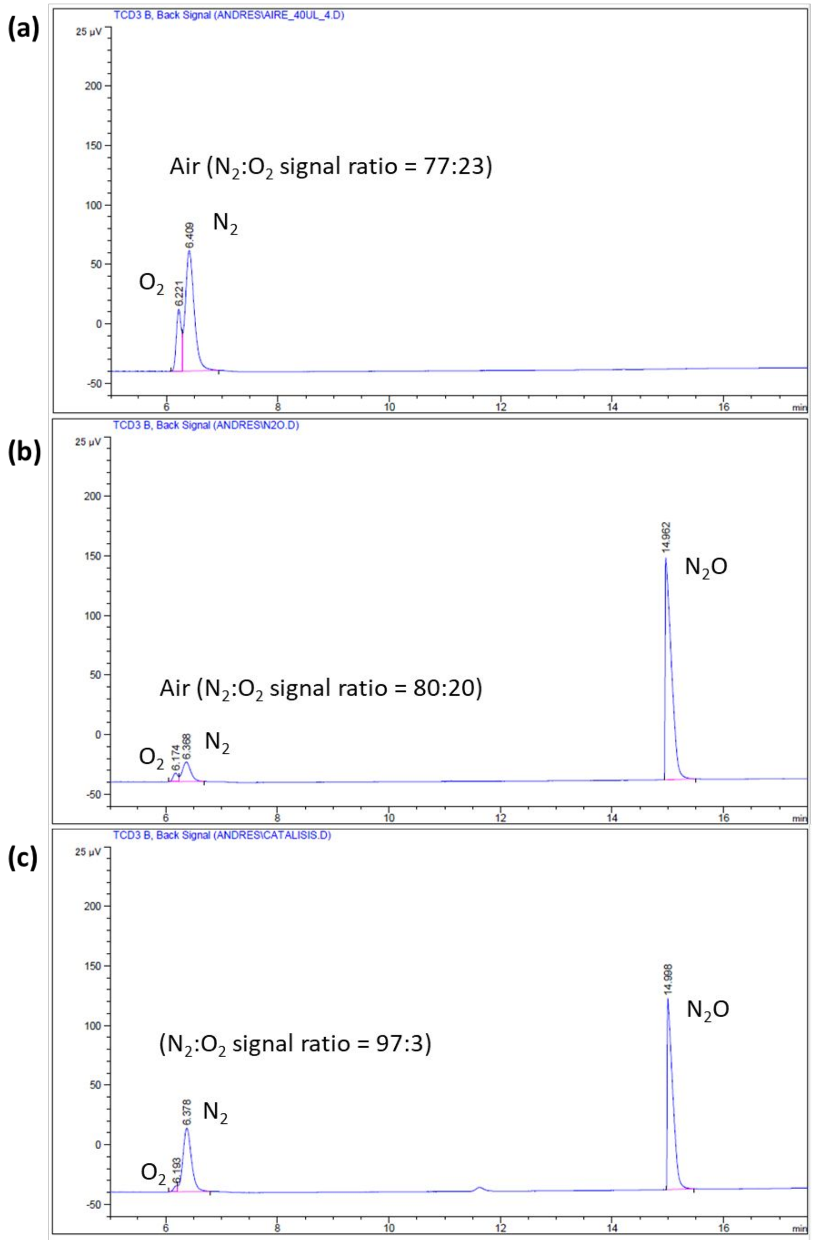

Figure S2. N_2 detection by GC-TCD: a) control experiment: air (N_2 + O_2); b) control experiment: N_2O; c) catalytic reaction (Table 1, entry 3).

2. NMR Spectra of Compounds 2-7

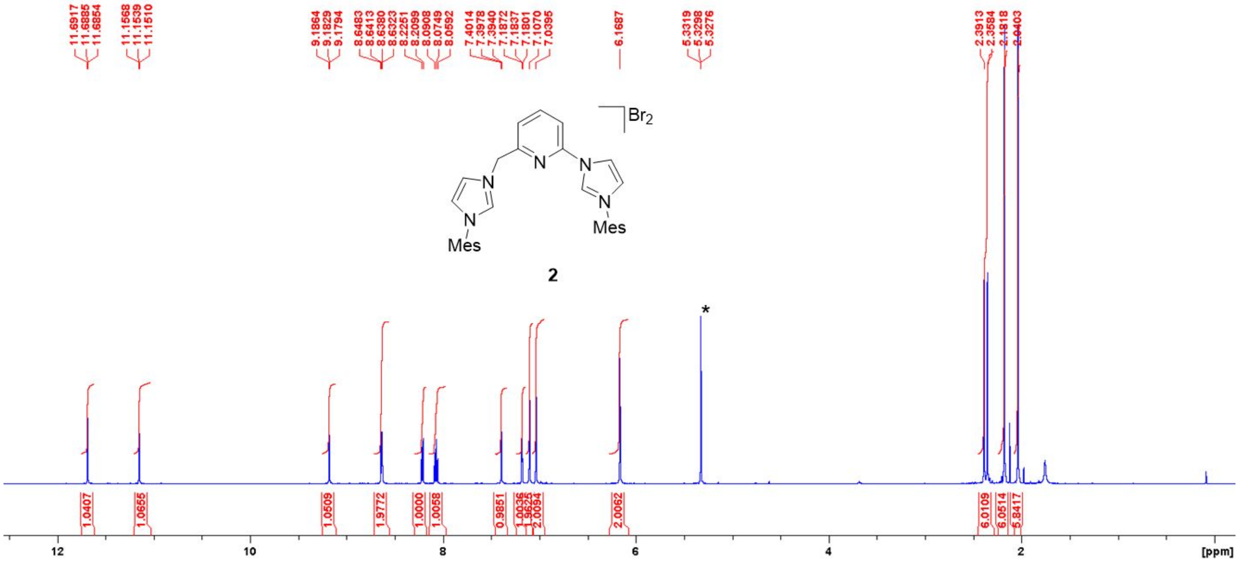

Figure S3. ^{1}H NMR spectrum of the imidazolium salt **2** (500 MHz, CD_2Cl_2). (*denotes residual deuterated solvent).

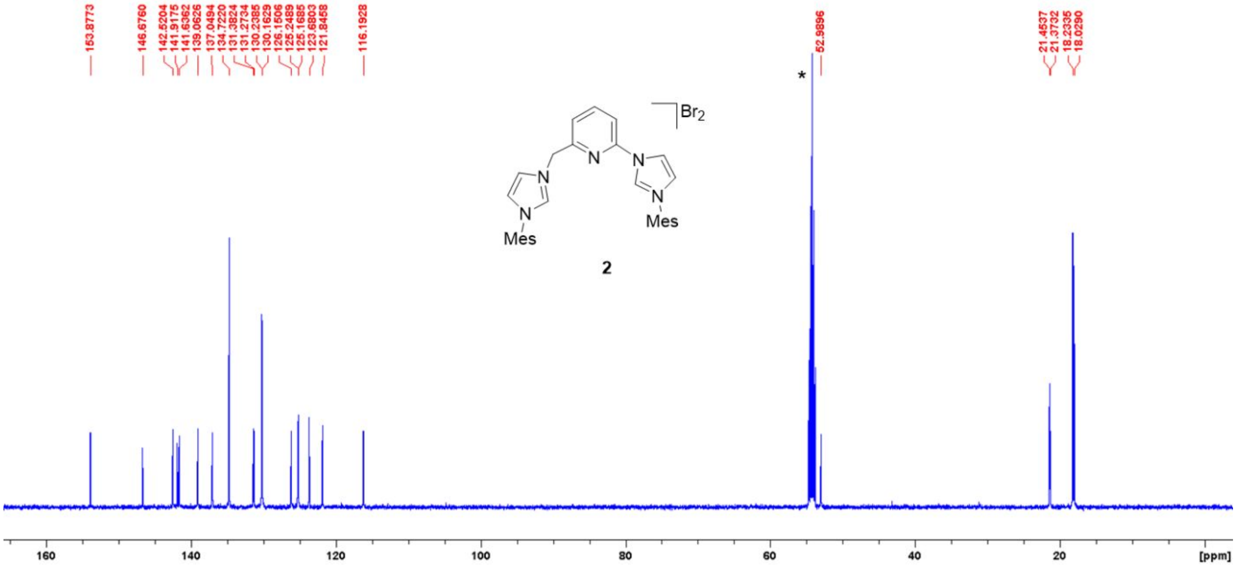

Figure S4. $^{13}C\{^{1}H\}$ NMR spectrum of the imidazolium salt **2** (126 MHz, CD_2Cl_2). (*denotes residual deuterated solvent).

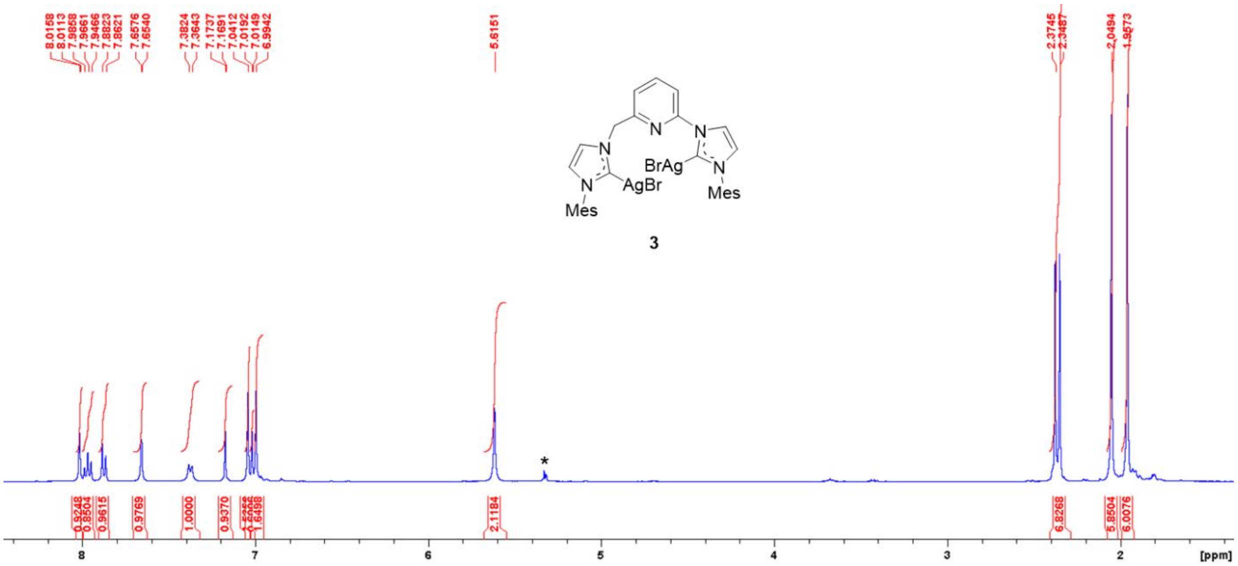

Figure S5. ^{1}H NMR spectrum of the silver complex **3** (400 MHz, CD_2Cl_2). (*denotes residual deuterated solvent).

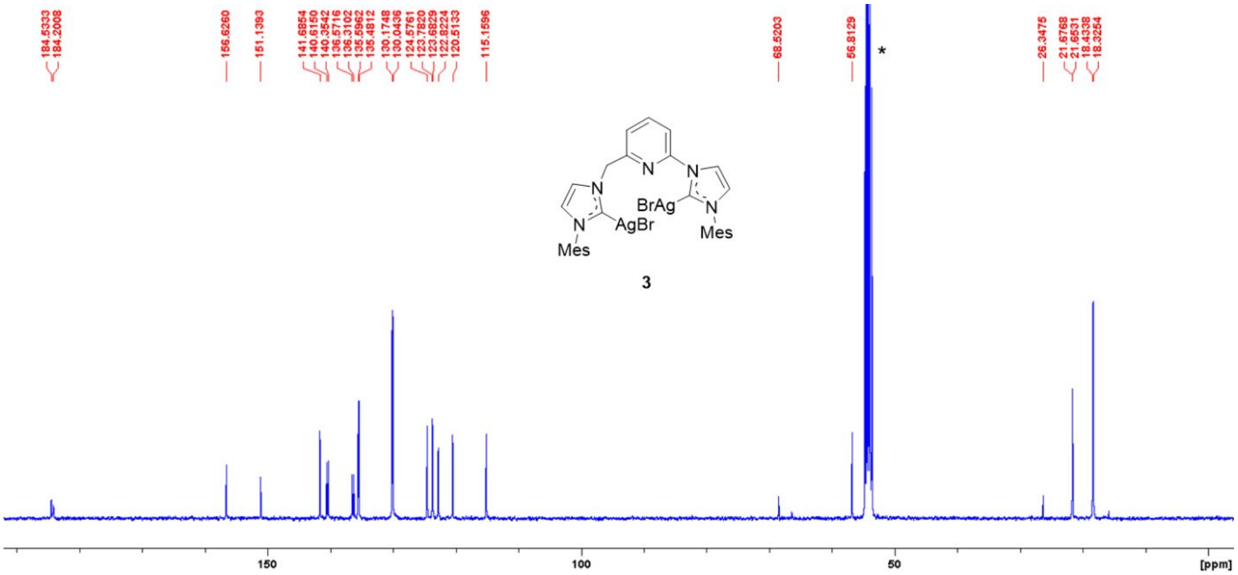

Figure S6. $^{13}C\{^{1}H\}$ NMR spectrum of the silver complex **3** (101 MHz, CD_2Cl_2). (*denotes residual deuterated solvent).

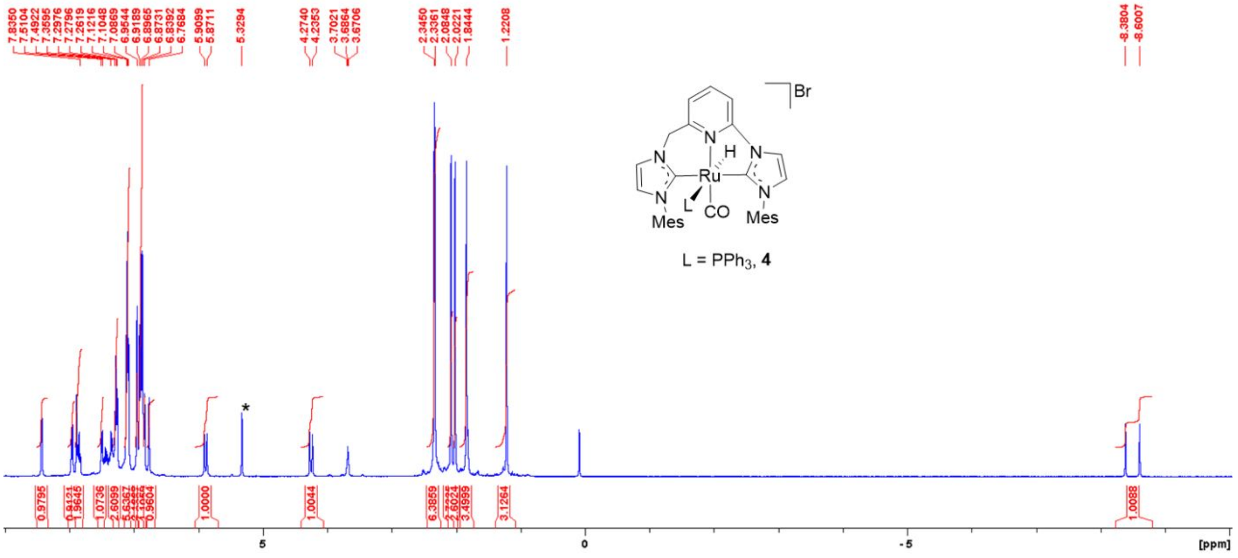

Figure S7. ^{1}H NMR spectrum of complex **4** (400 MHz, CD_2Cl_2). (*denotes residual deuterated solvent).

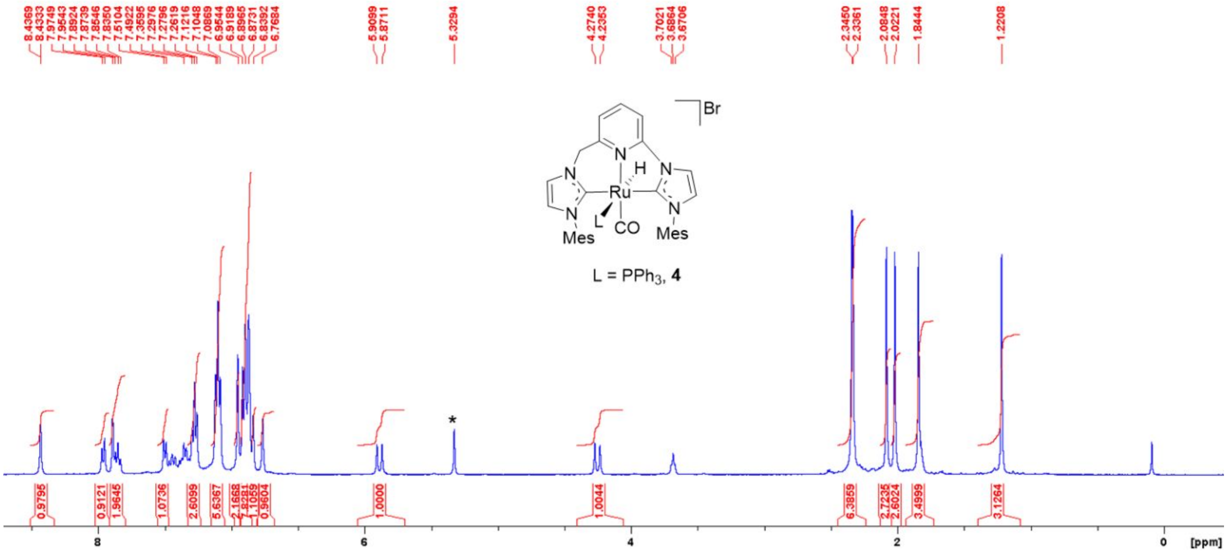

Figure S8. Region (0.0 to 9.0 ppm) of the ^{1}H NMR spectrum of complex **4** (400 MHz, CD_2Cl_2). (*denotes residual deuterated solvent).

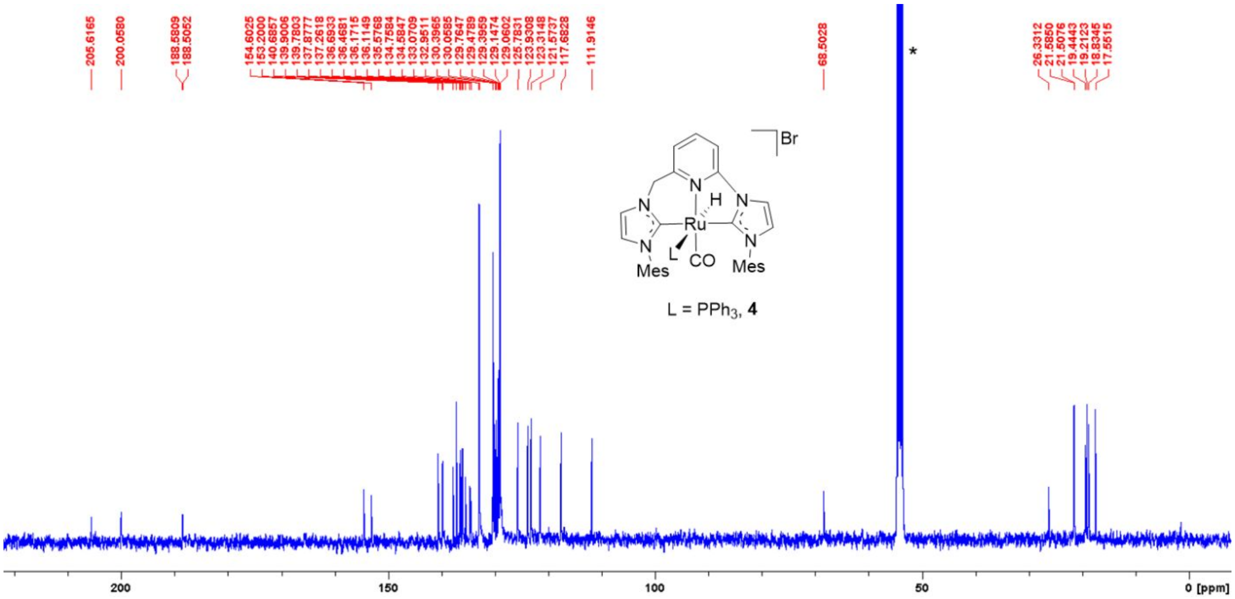

Figure S9. $^{13}C\{^{1}H\}$ NMR spectrum of complex **4** (101 MHz, CD_2Cl_2). (*denotes residual deuterated solvent).

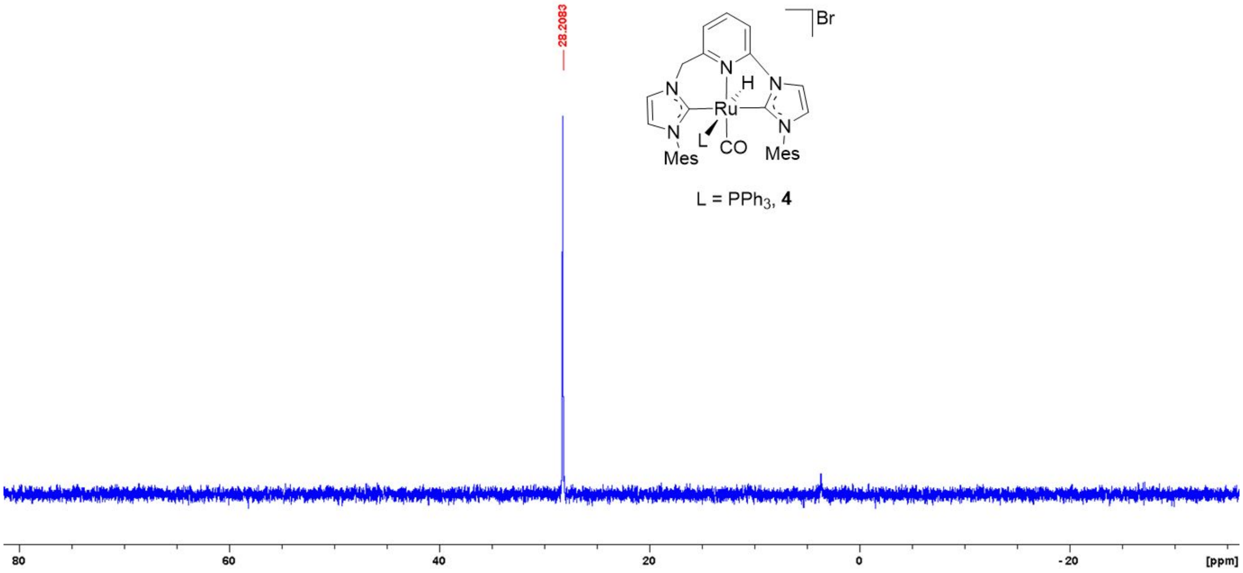

Figure S10. $^{13}P\{^{1}H\}$ NMR spectrum of complex **4** (162 MHz, CD_2Cl_2).

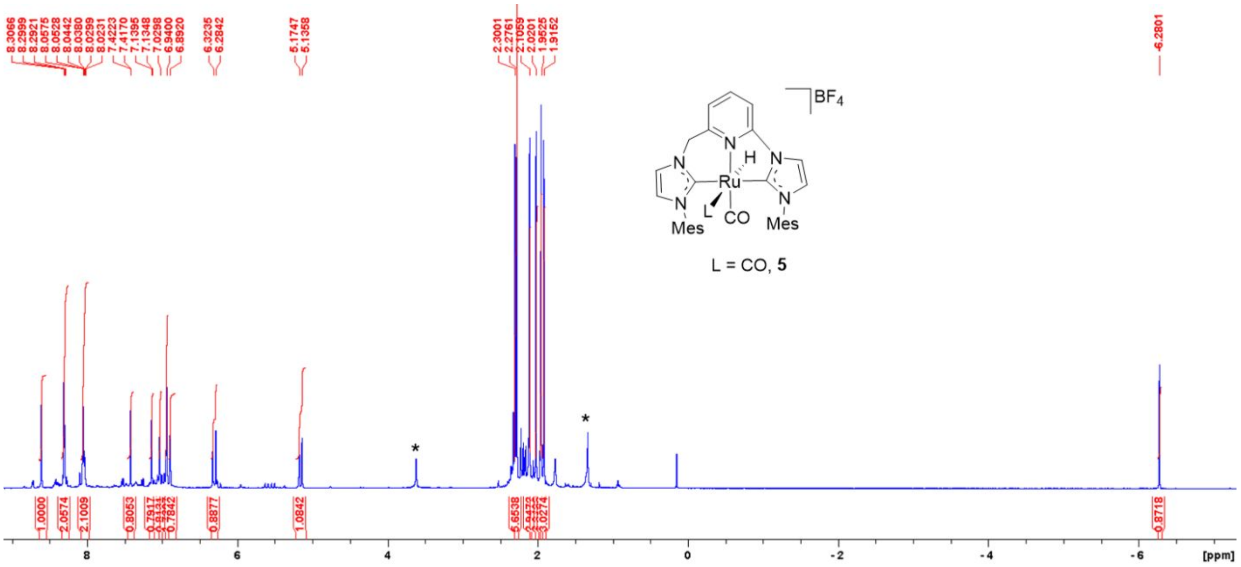

Figure S11. ^{1}H NMR spectrum of complex **5** (400 MHz, THF-d_8). (*denotes residual deuterated solvent).

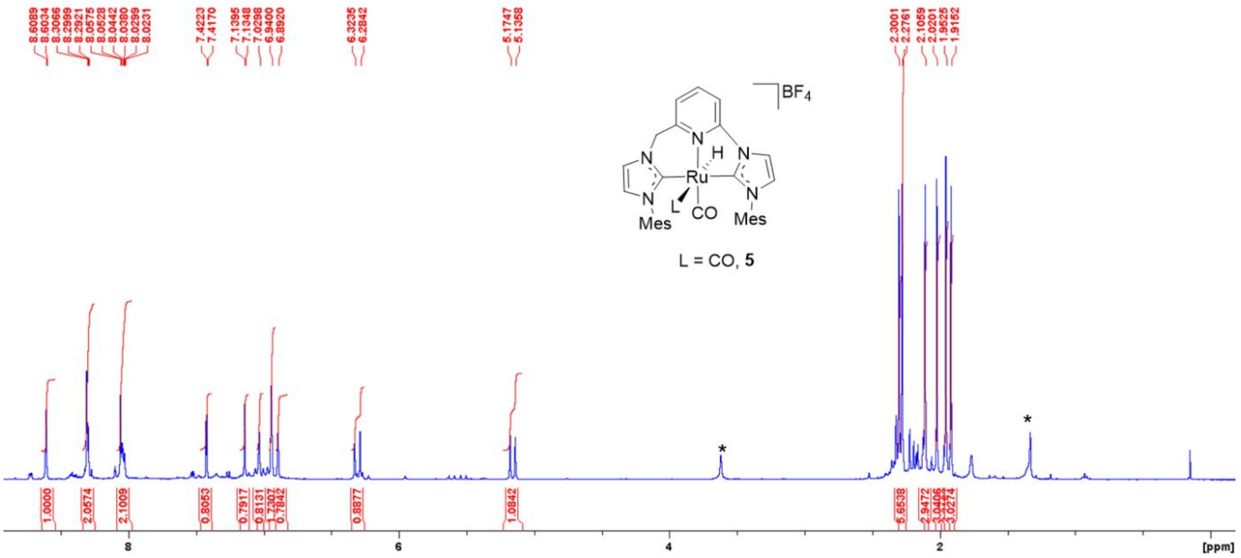

Figure S12. Region (0.0 to 9.0 ppm) of the ^{1}H NMR spectrum of complex **5** (400 MHz, THF-d_8). (*denotes residual deuterated solvent).

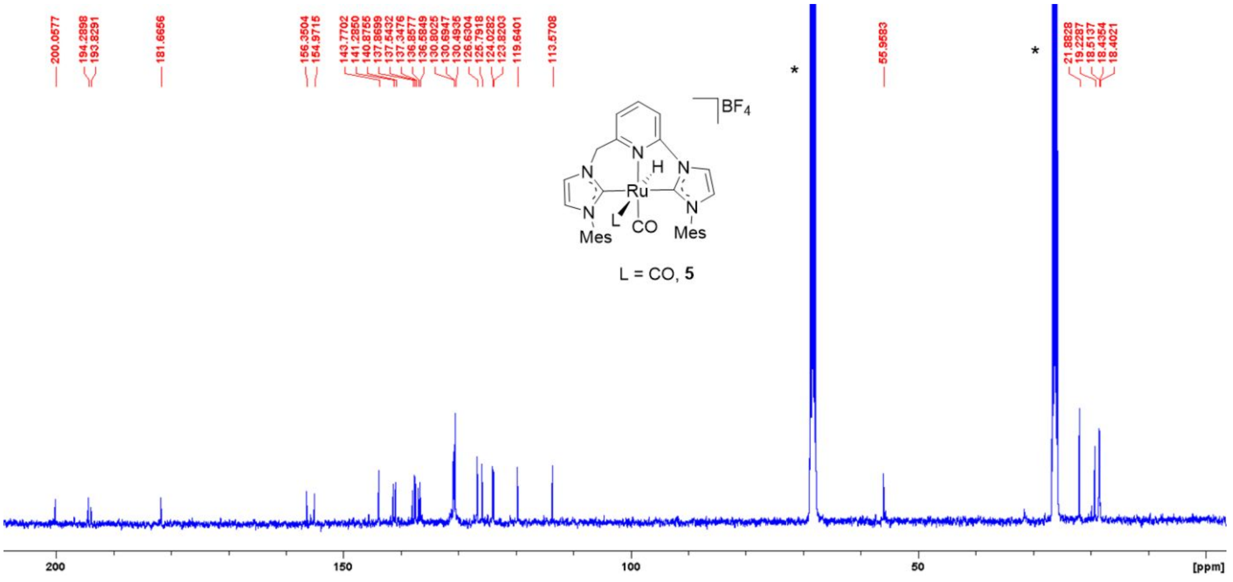

Figure S13. $^{13}C\{^1H\}$ NMR spectrum of complex **5** (101 MHz, THF-d_8). (*denotes residual deuterated solvent).

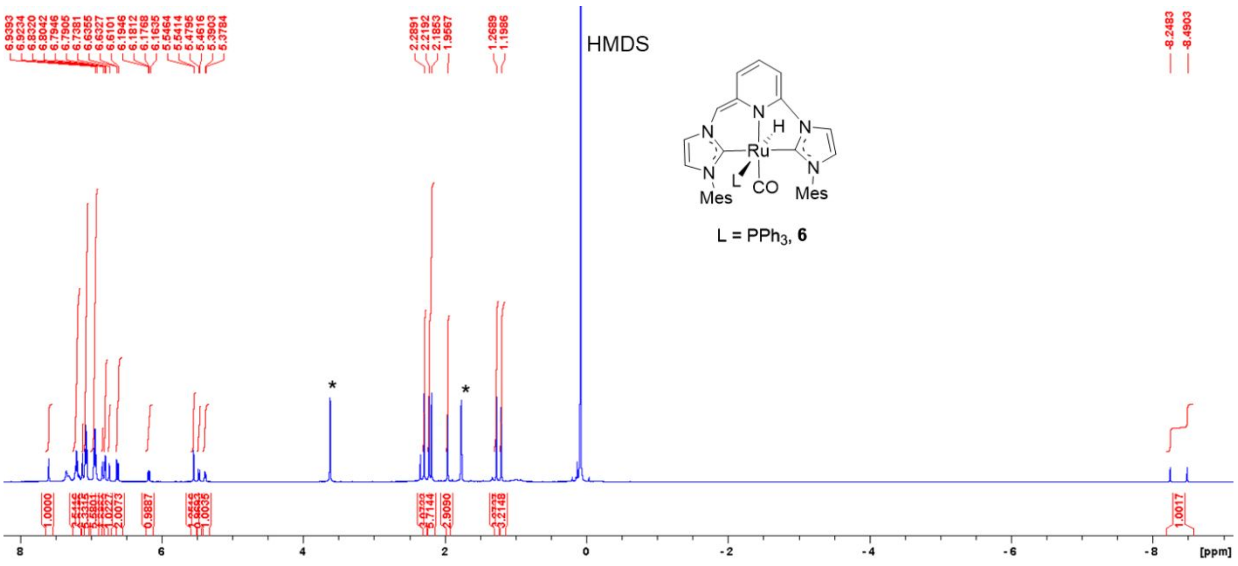

Figure S14. ^1H NMR spectrum of complex **6** (500 MHz, THF-d_8) generated by reaction of **4** and KHMDS. (*denotes residual deuterated solvent).

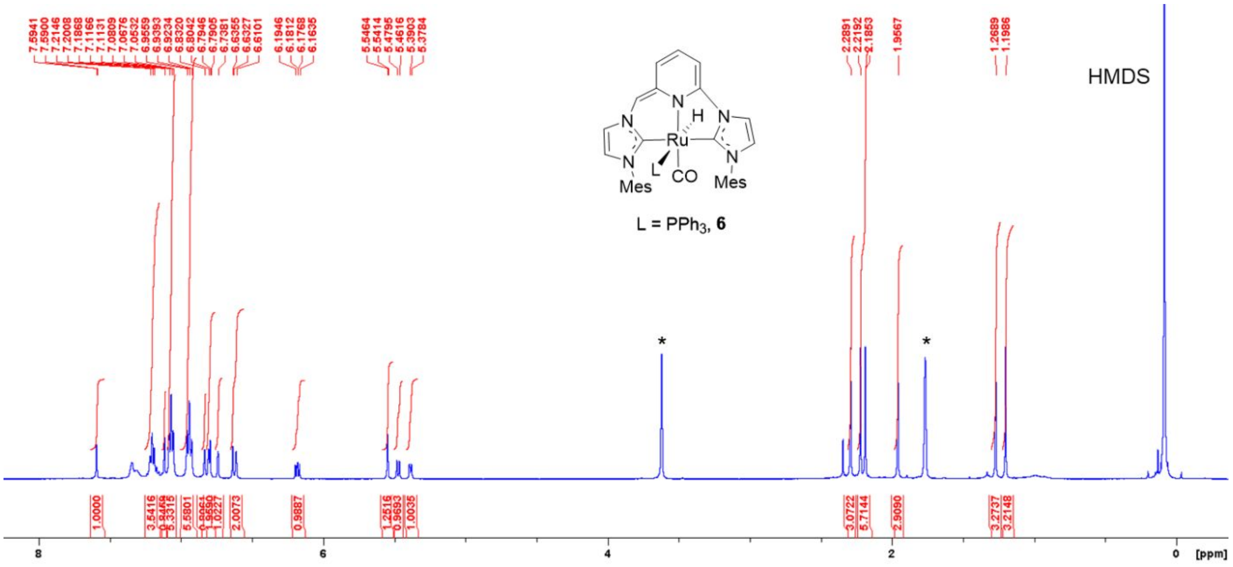

Figure S15. Region (0.0 to 8.0 ppm) of the ^{1}H NMR spectrum of complex **6** (500 MHz, THF-d_8) generated by reaction of **4** and KHMDS. (*denotes residual deuterated solvent).

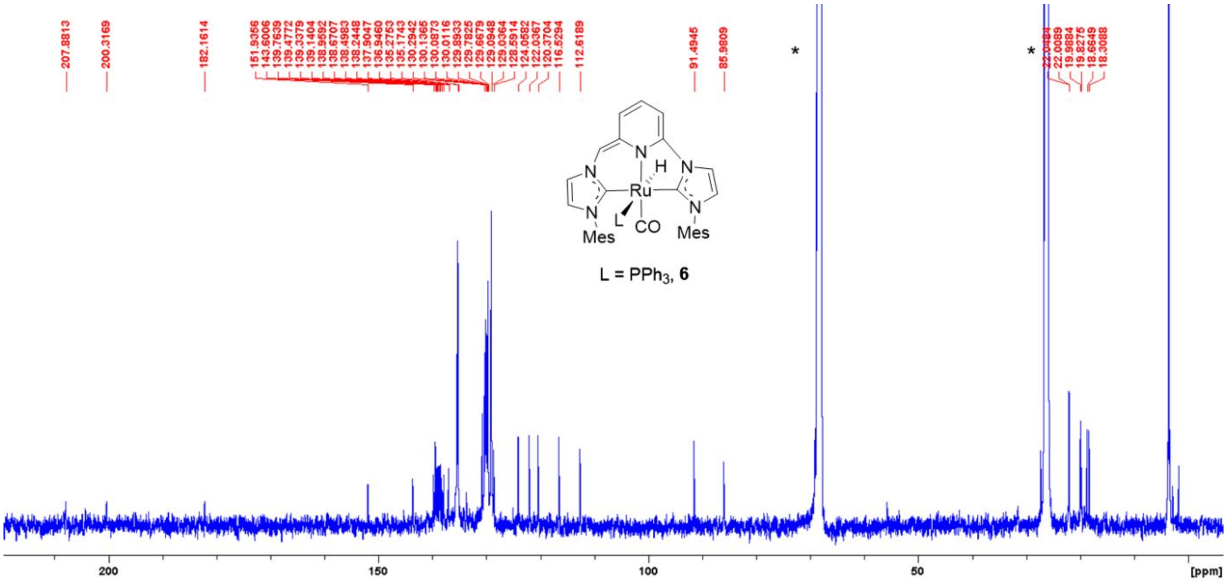

Figure S16. $^{13}C\{^{1}H\}$ NMR spectrum of complex **6** (126 MHz, THF-d_8) generated by reaction of **4** and KHMDS. (*denotes residual deuterated solvent).

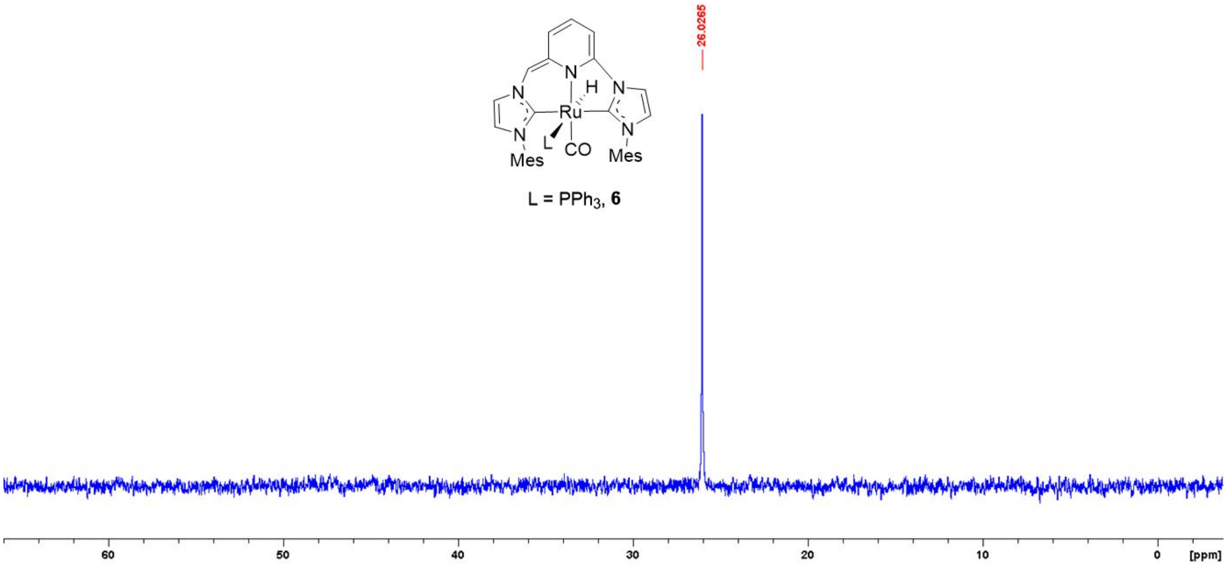

Figure S17. $^{13}P\{^{1}H\}$ NMR spectrum of complex **6** (202 MHz, THF-d_8) generated by reaction of **4** and KHMDS.

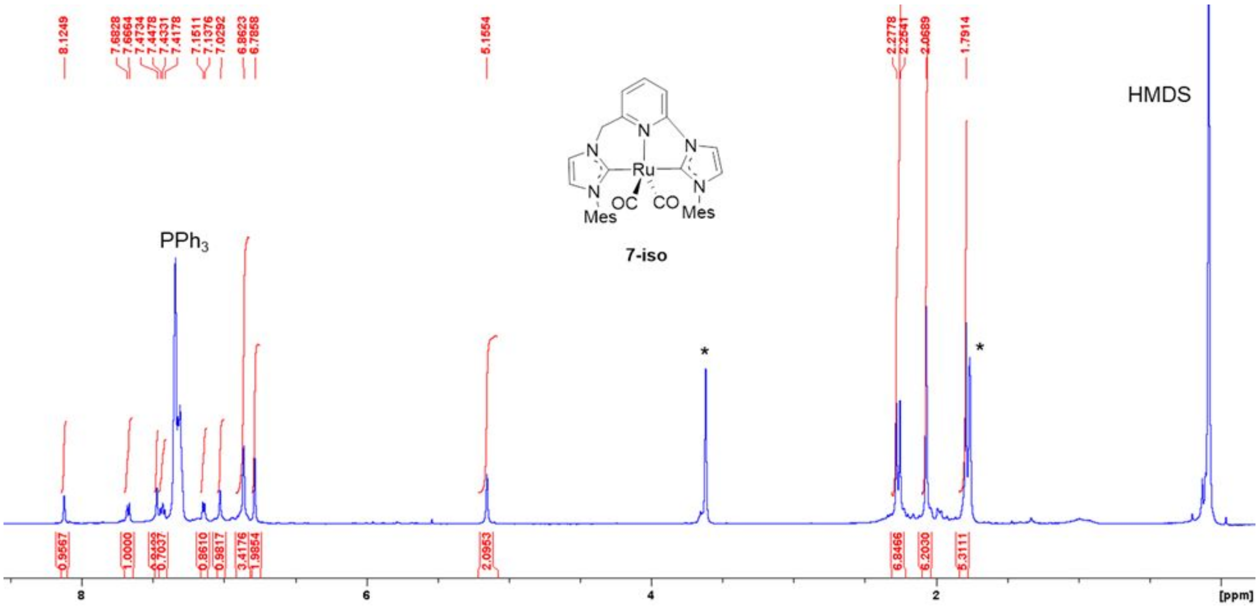

Figure S18. ^{1}H NMR spectrum of complex **7-iso** (500 MHz, THF-d_8) generated by reaction of **4**, KHMDS and CO. (*denotes residual deuterated solvent).

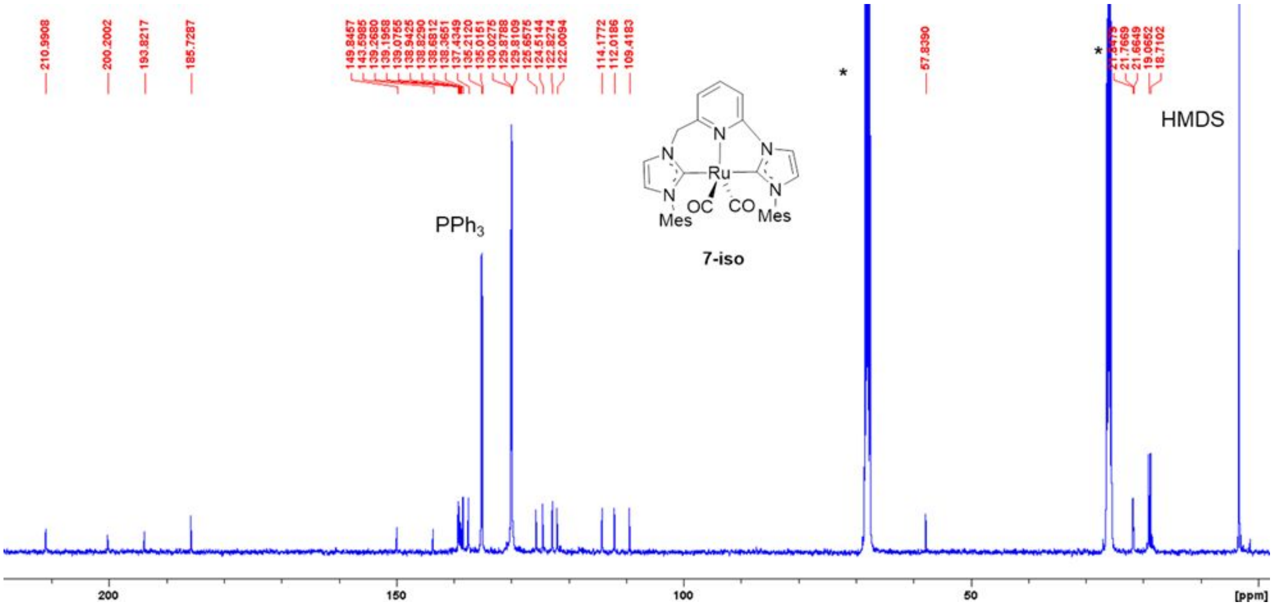

Figure S19. ^{13}C NMR spectrum of complex **7-iso** (101 MHz, THF-d_8) generated by reaction of **4**, KHMDS and CO. (*denotes residual deuterated solvent).

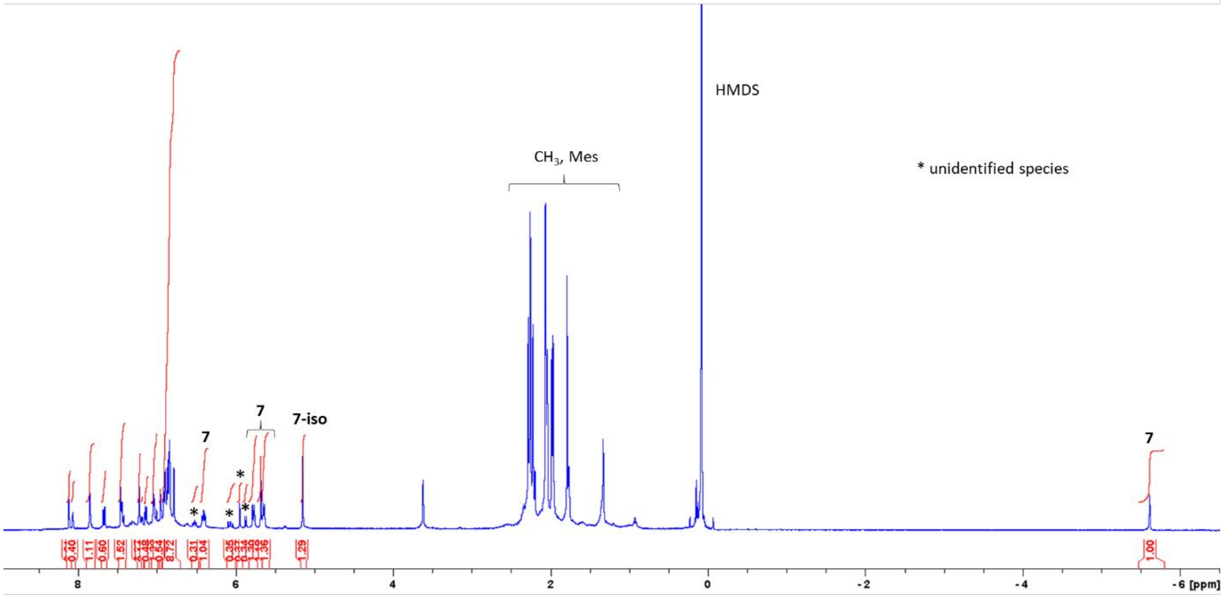

Figure S20. ^{1}H NMR spectrum of the reaction of **5** with KHMDS in THF-d_8 after 10 min (400 MHz).

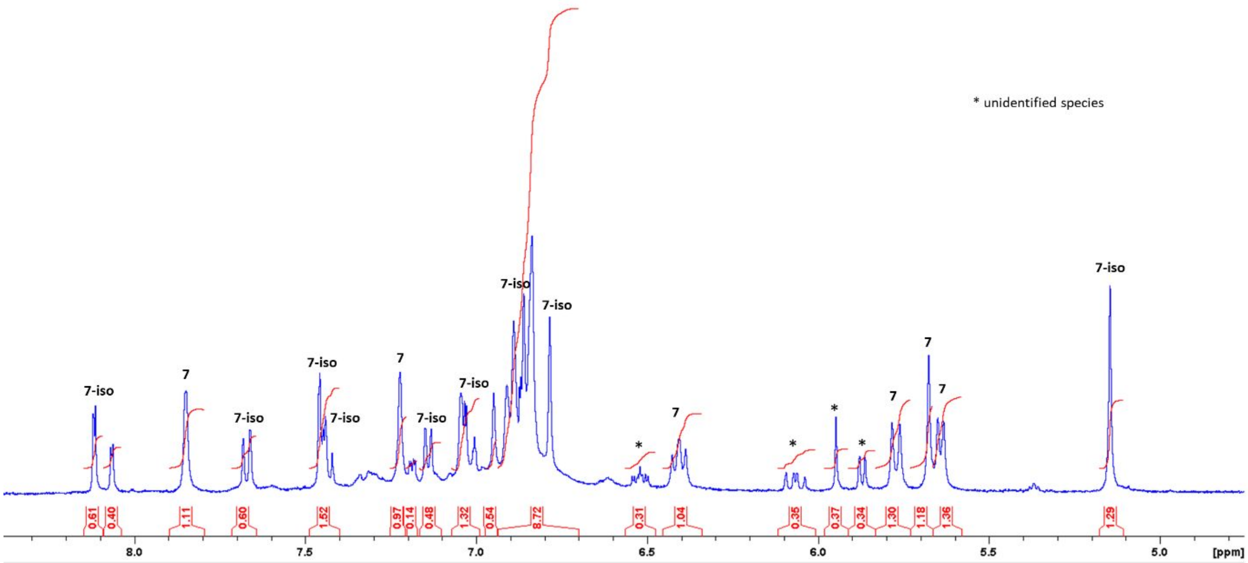

Figure S21. Enlarge region (8.4-4.8 ppm) of the ^{1}H NMR spectrum of the reaction of **5** with KHMDS in THF-d_8 after 10 min (400 MHz).

3. NMR Spectra of Catalysis Products

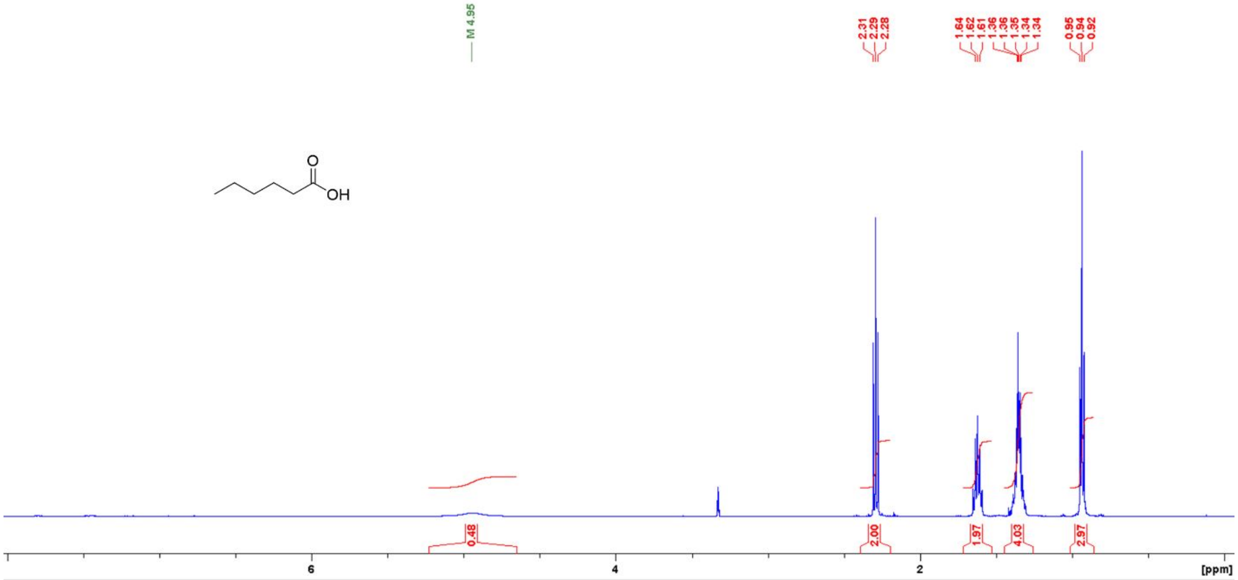

Figure S22. ^{1}H NMR spectrum of hexanoic acid (500 MHz, CD_3OD) (Table 1, entry 3).

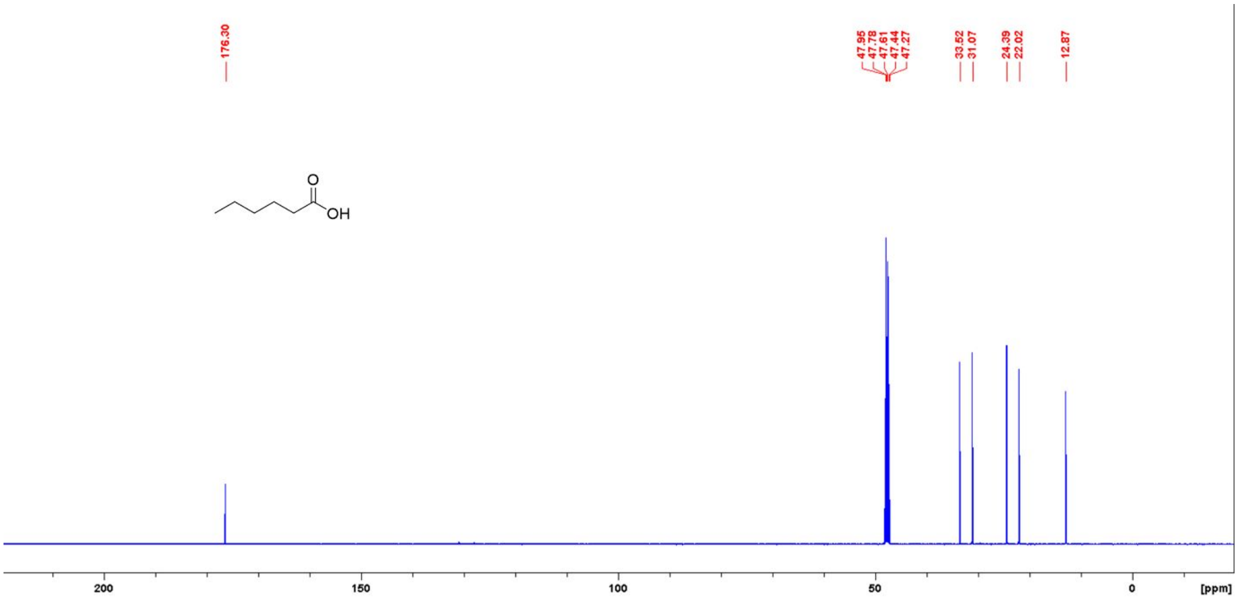

Figure S23. $^{13}C\{^1H\}$ NMR spectrum of hexanoic acid (126 MHz, CD_3OD) (Table 1, entry 3).

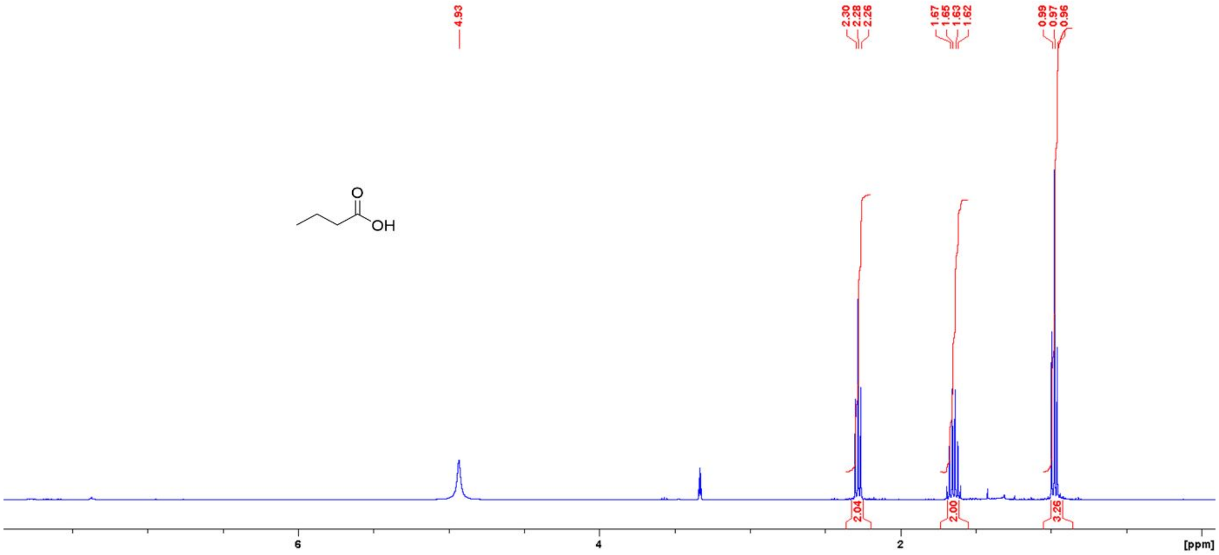

Figure S24. ^1H NMR spectrum of butyric acid (400 MHz, CD_3OD) (Table 1, entry 8).

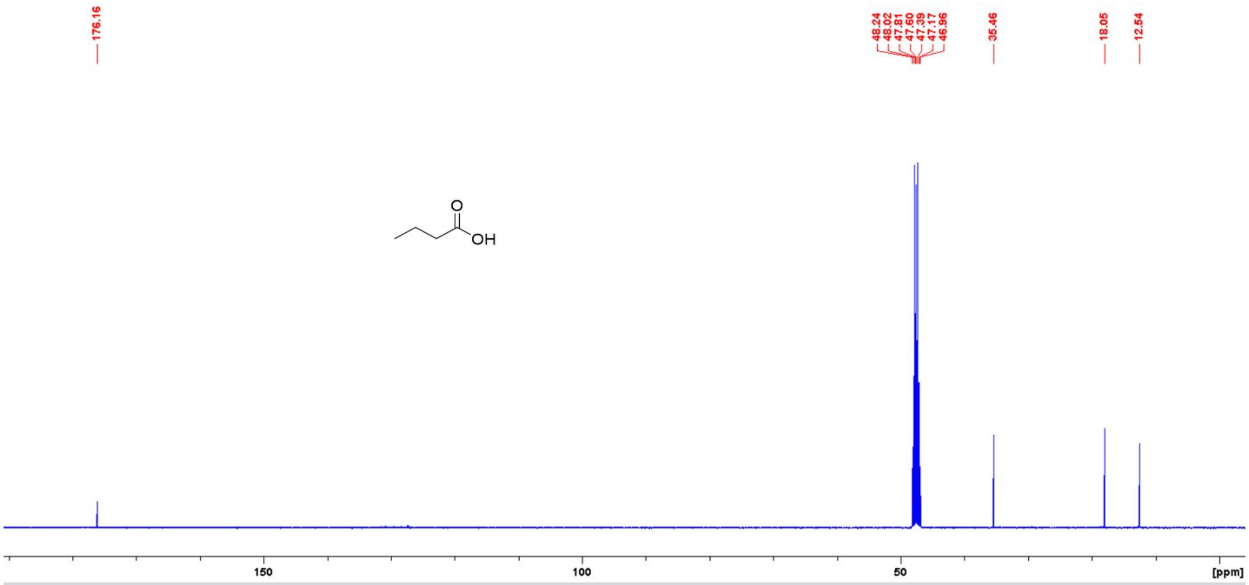

Figure S25. $^{13}C\{^{1}H\}$ NMR spectrum of butyric acid (101 MHz, CD_3OD) (Table 1, entry 8).

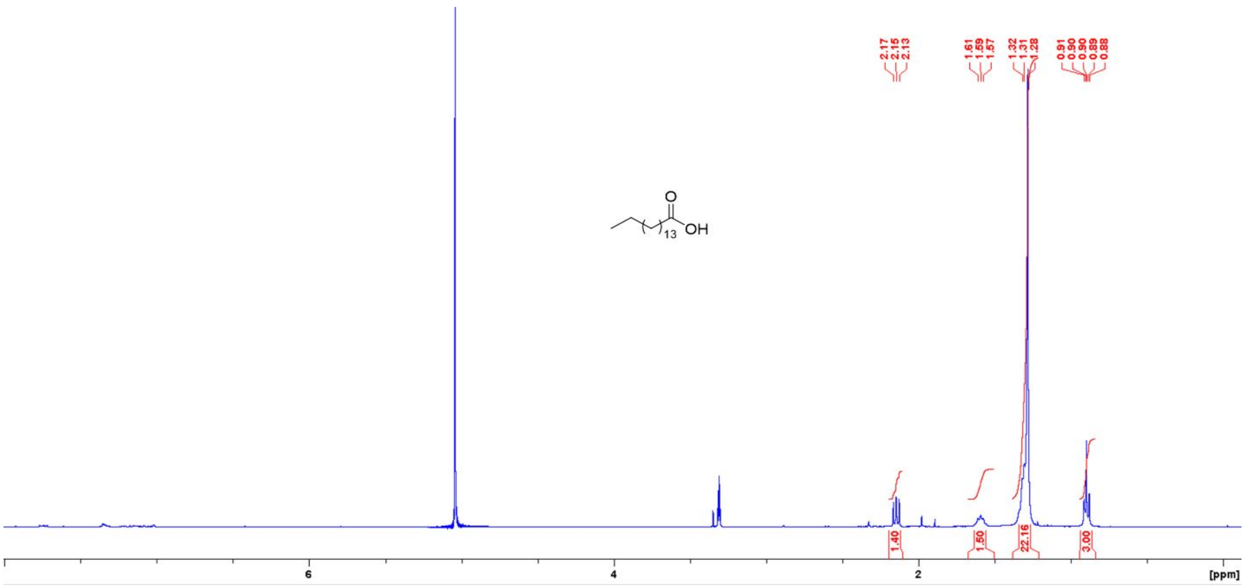

Figure S26. ^{1}H NMR spectrum of palmitic acid (400 MHz, CD_3OD) (Table 1, entry 9).

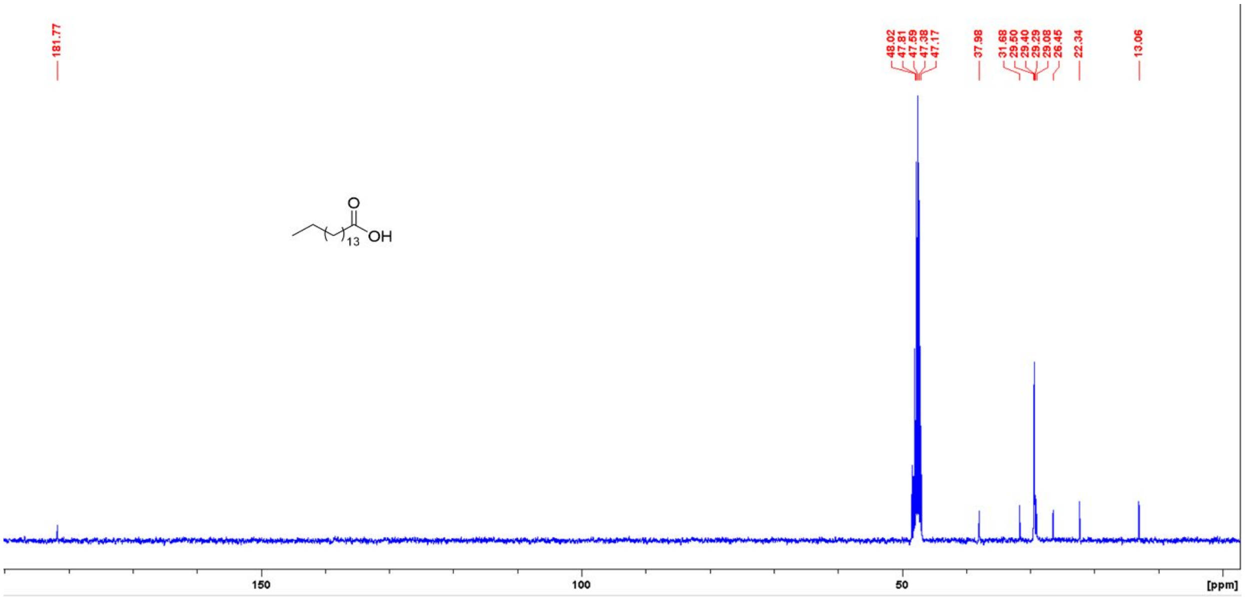

Figure S27. $^{13}C\{^1H\}$ NMR spectrum of palmitic acid (101 MHz, CD_3OD) (Table 1, entry 9).

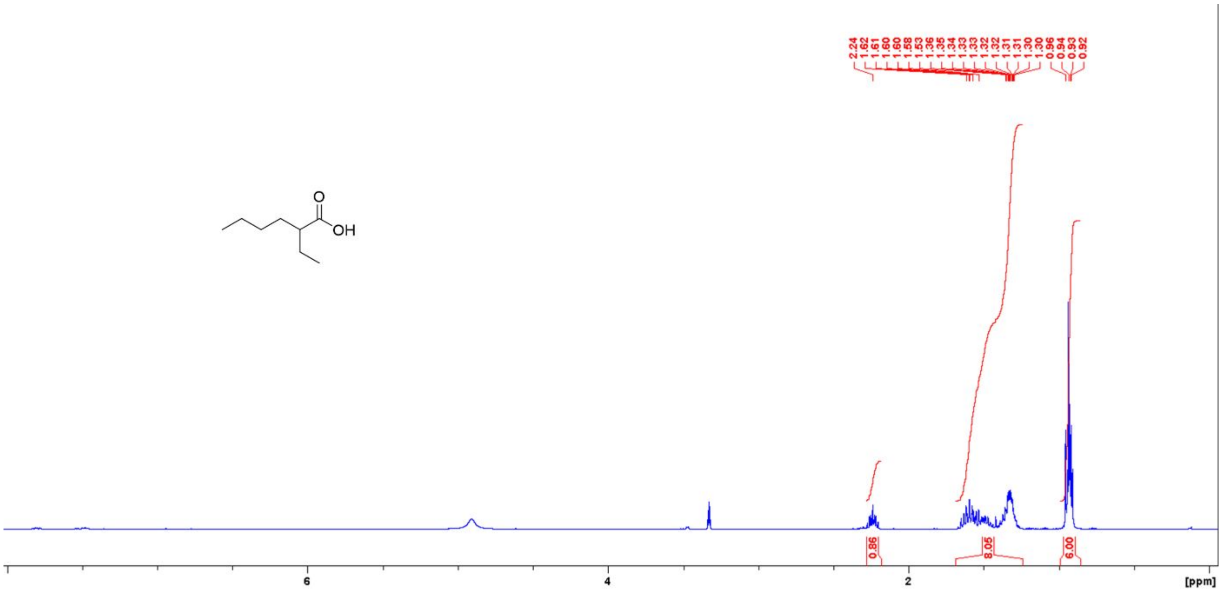

Figure S28. ^1H NMR spectrum of 2-ethylhexanoic acid (400 MHz, CD_3OD) (Table 1, entry 11).

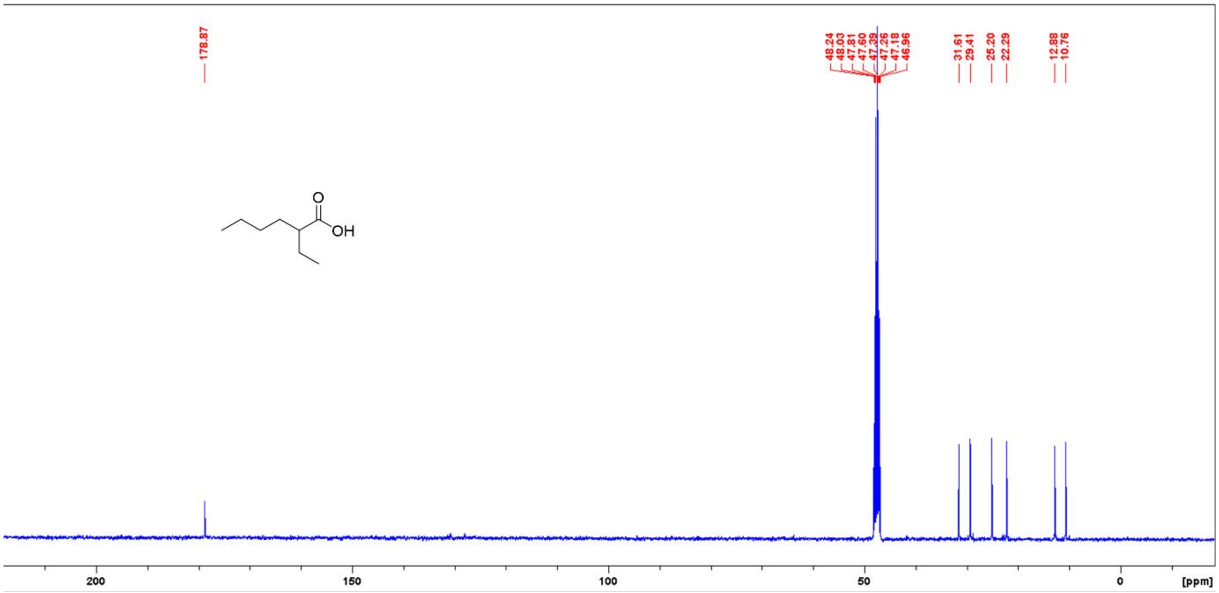

Figure S29. $^{13}C\{^{1}H\}$ NMR spectrum of 2-ethylhexanoic acid (101 MHz, CD_3OD) (Table 1, entry 11).

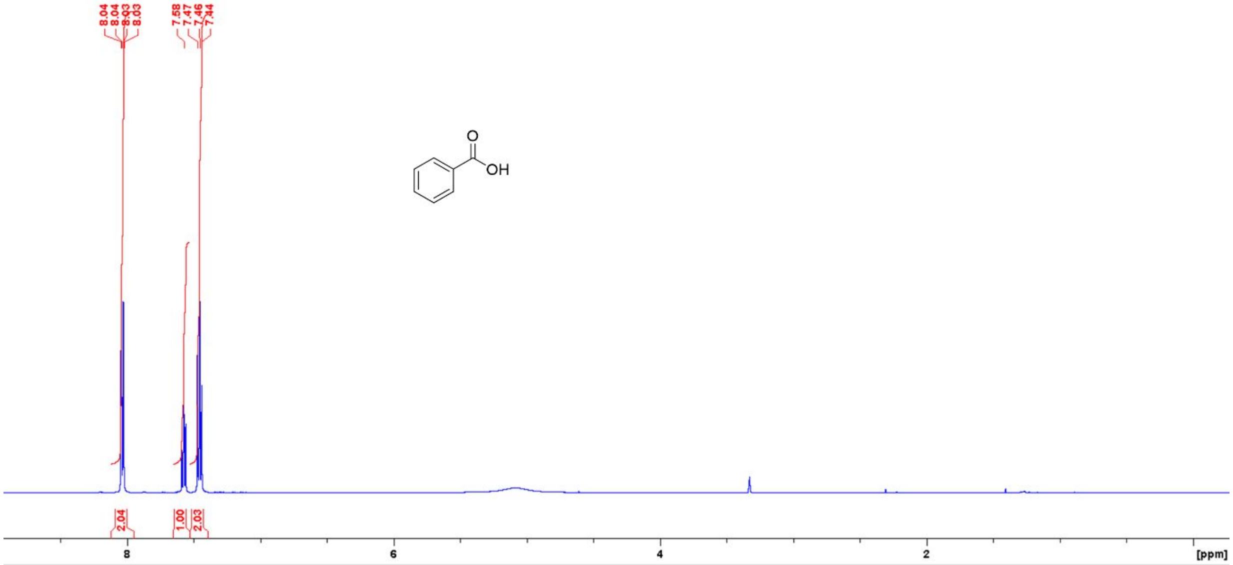

Figure S30. ^{1}H NMR spectrum of benzoic acid (500 MHz, CD_3OD) (Table 1, entry 12).

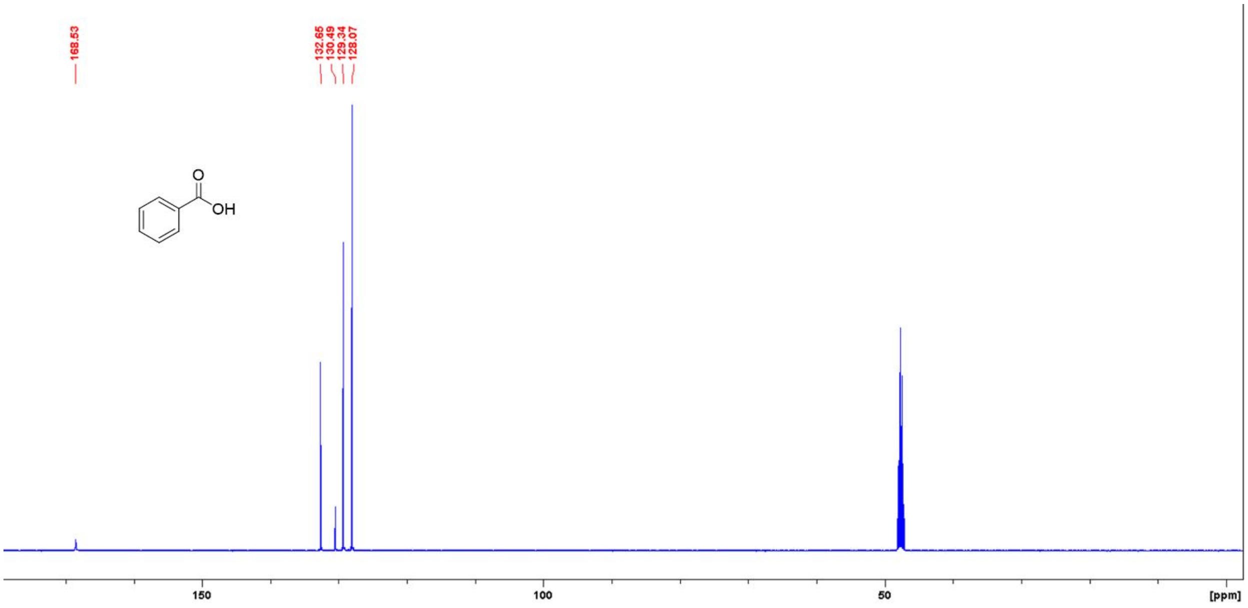

Figure S31. $^{13}C\{^1H\}$ NMR spectrum of benzoic acid (126 MHz, CD_3OD) (Table 1, entry 12).

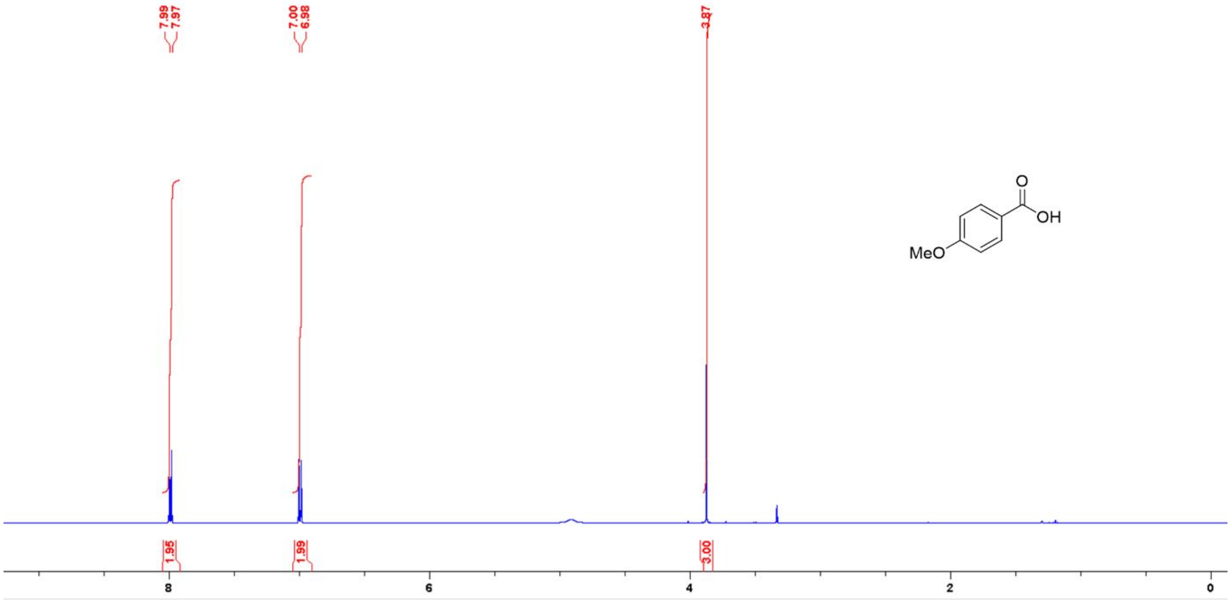

Figure S32. ^1H NMR spectrum of 4-methoxybenzoic acid (500 MHz, CD_3OD) (Table 1, entry 13).

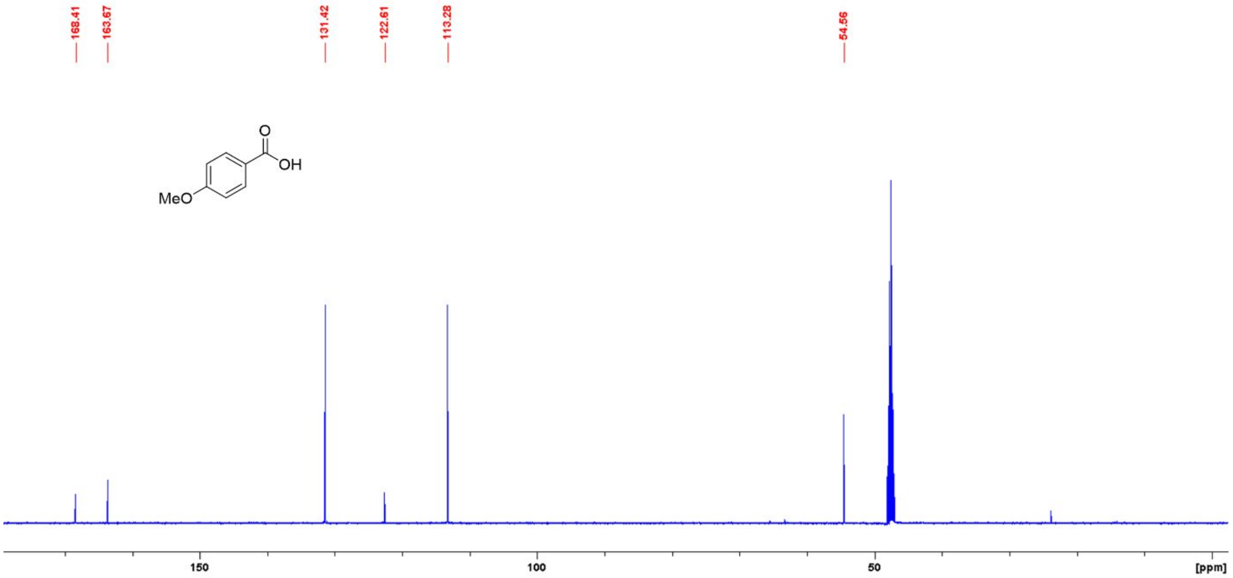

Figure S33. $^{13}C\{^{1}H\}$ NMR spectrum of 4-methoxybenzoic acid (126 MHz, CD_3OD) (Table 1, entry 13).

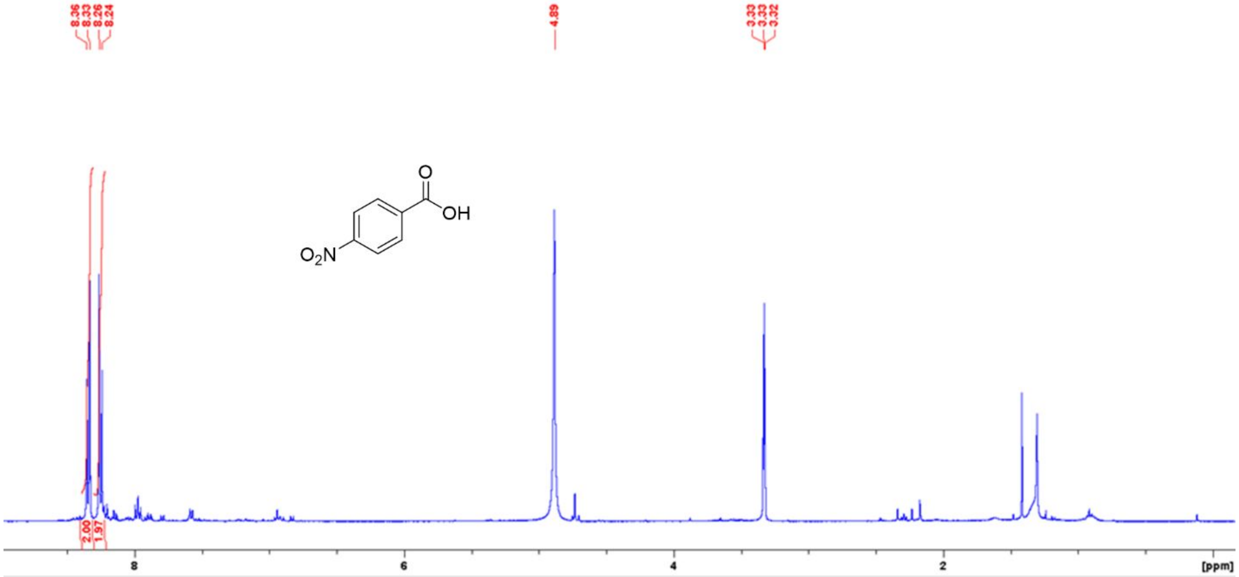

Figure S34. ^{1}H NMR spectrum of 4-nitrobenzoic acid (400 MHz, CD_3OD) (Table 1, entry 14).

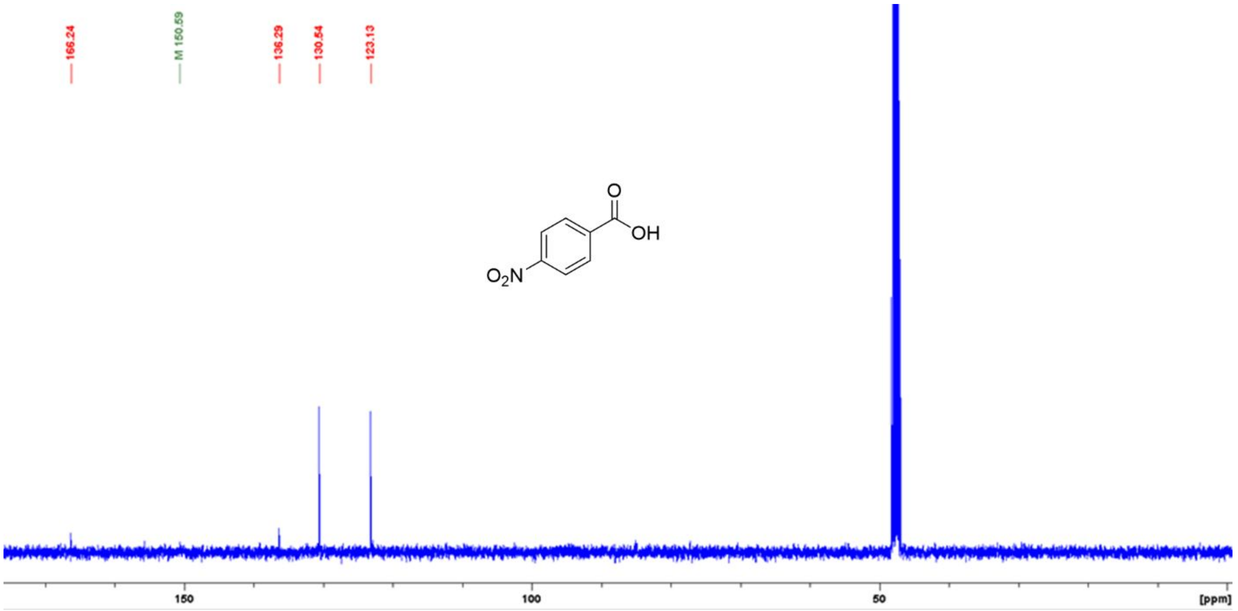

Figure S35. $^{13}C\{^{1}H\}$ NMR spectrum of 4-nitrobenzoic acid (101 MHz, CD_3OD) (Table 1, entry 14).

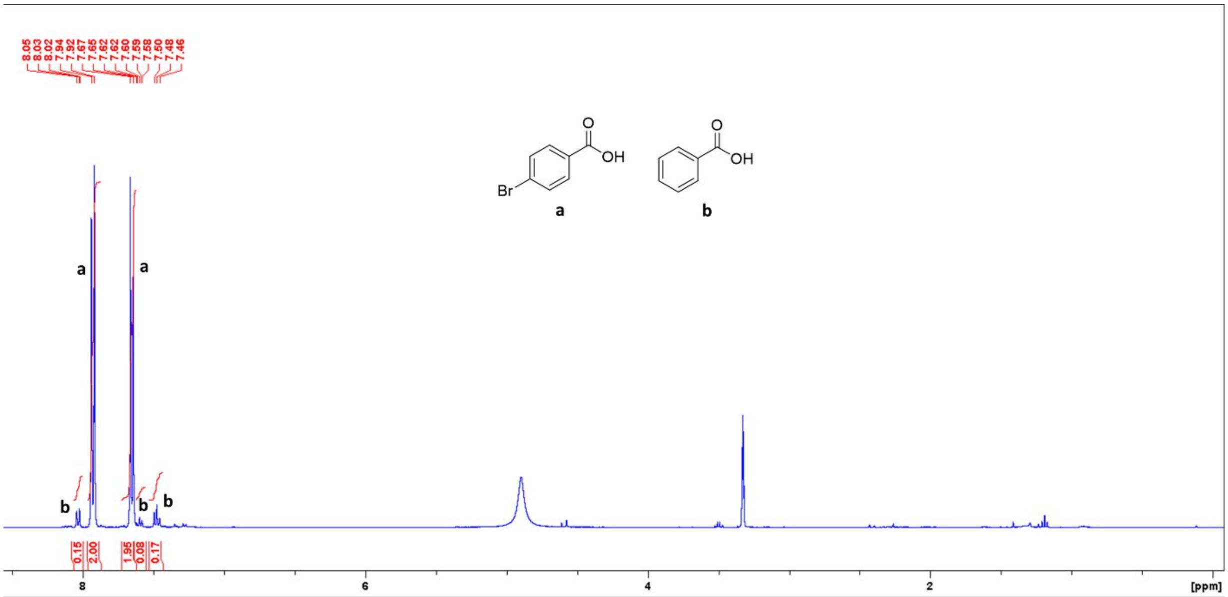

Figure S36. ^{1}H NMR spectrum of 4-bromobenzoic acid and benzoic acid (400 MHz, CD_3OD) (Table 1, entry 15).

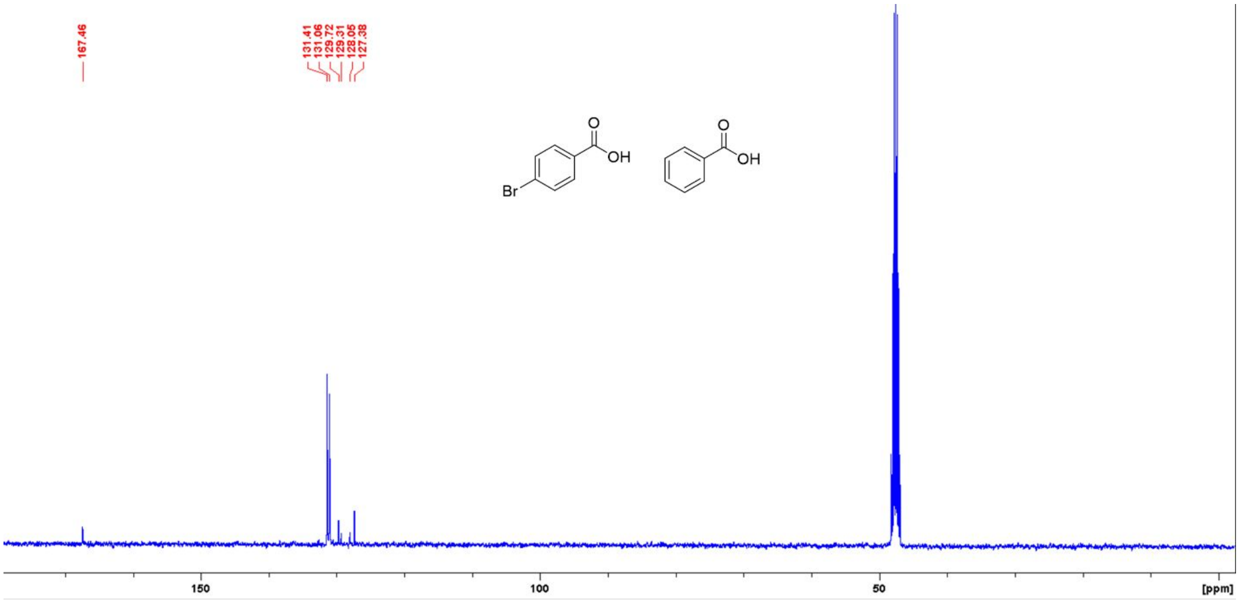

Figure S37. $^{13}C\{^{1}H\}$ NMR spectrum of 4-nitrobenzoic acid and benzoic acid (101 MHz, CD_3OD) (Table 1, entry 15).

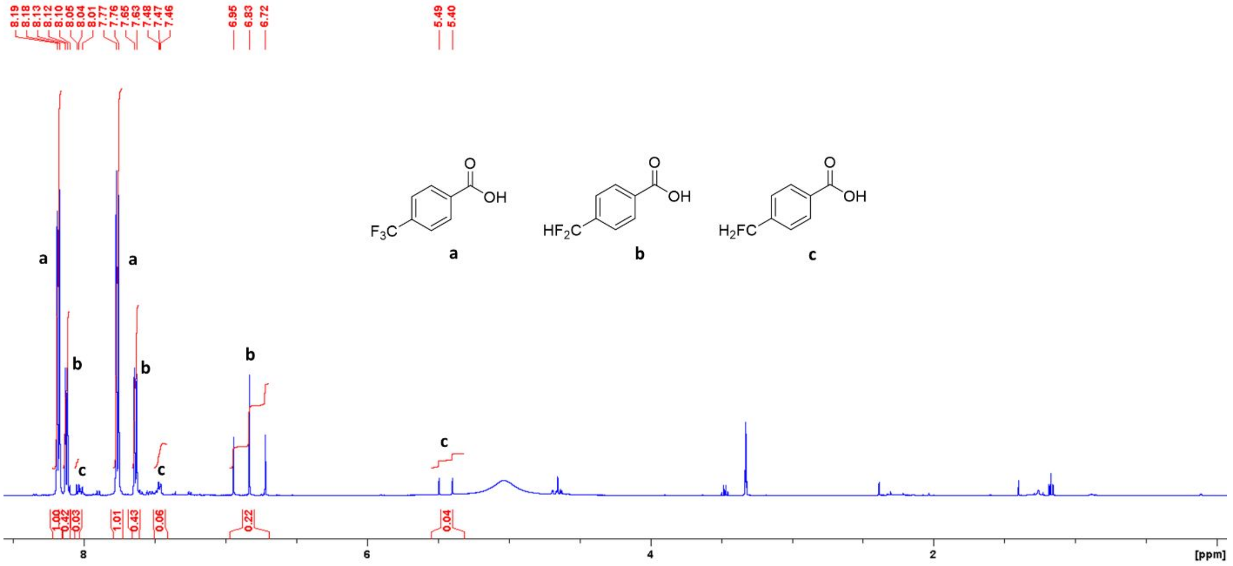

Figure S38. ^{1}H NMR spectrum of 4-(trifluoromethyl)benzoic acid, 4-(difluoromethyl)benzoic acid and 4-(fluoromethyl)benzoic acid (500 MHz, CD_3OD) (Table 1, entry 16).

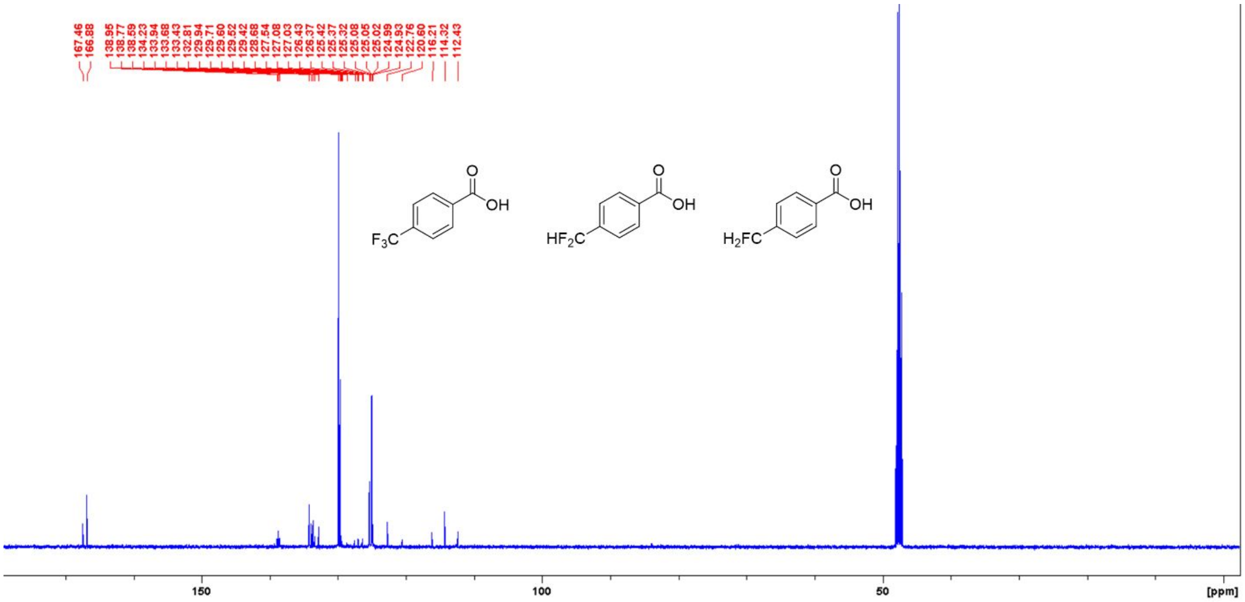

Figure S39. $^{13}C\{^1H\}$ NMR spectrum of 4-(trifluoromethyl)benzoic acid, 4-(difluoromethyl)benzoic acid and 4-(fluoromethyl)benzoic acid (126 MHz, CD_3OD) (Table 1, entry 16).

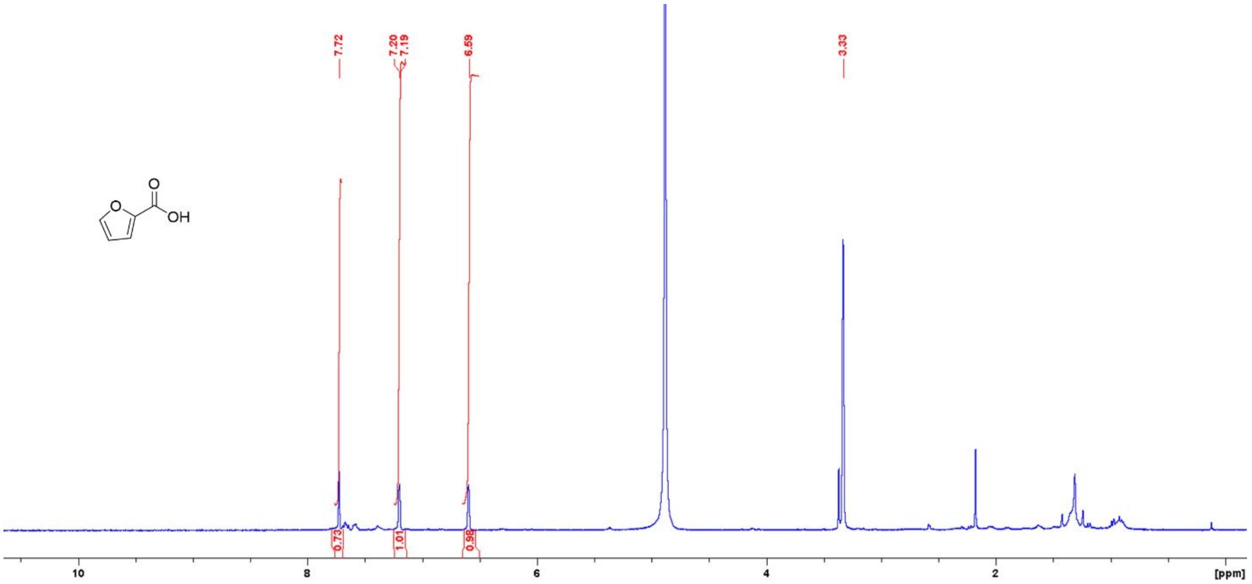

Figure S40. ^1H NMR spectrum of 2-furoic acid (400 MHz, CD_3OD) (Table 1, entry 17).

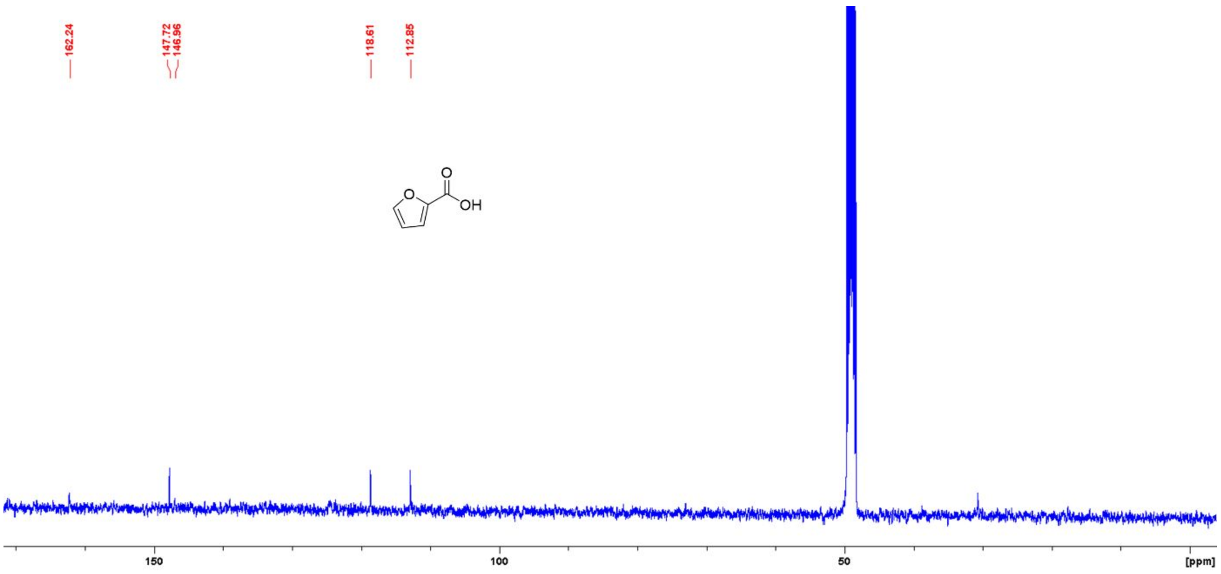

Figure S41. $^{13}C\{^1H\}$ NMR spectrum of 2-furoic acid (126 MHz, CD_3OD) (Table 1, entry 17).

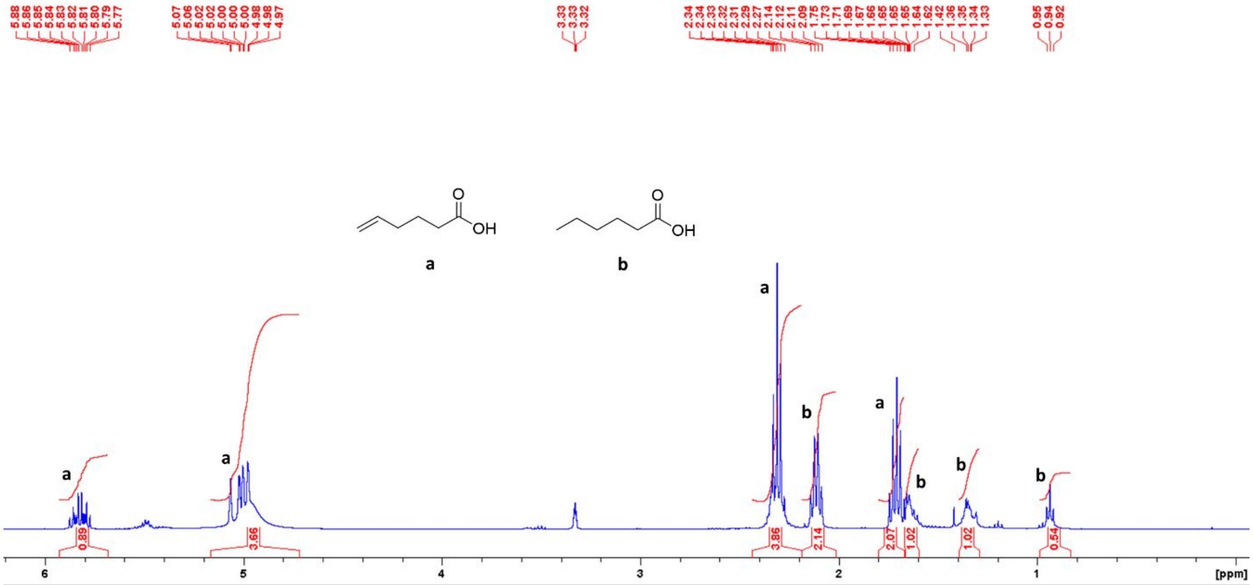

Figure S42. ^1H NMR spectrum of 5-hexenoic and hexanoic acids (400 MHz, CD_3OD) (Table 1, entry 18).

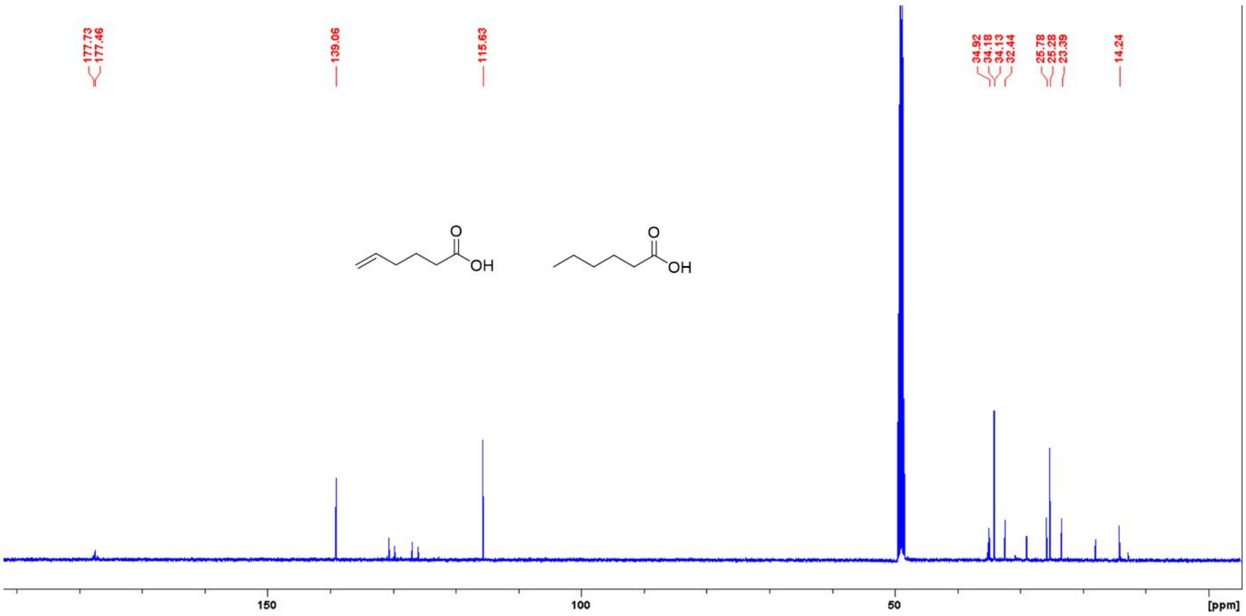

Figure S43. $^{13}C\{^{1}H\}$ NMR spectrum of 5-hexenoic and hexanoic acids (126 MHz, CD_3OD) (Table 1, entry 18).

4. X-Ray Structural Analysis of Complexes 4 and 6

Crystals suitable for X-ray diffraction analysis were coated with dry perfluoropolyether, mounted on glass fibres, and fixed in a cold nitrogen stream to the goniometer head. Data collections[2] were performed on a Bruker-AXS, D8 Quest ECO diffractometer equipped with a micro-focus ImS 3.0 source, using graphite monochromatized Mo radiation λ(Mo Kα) = 0.71073 Å and an area detector (Bruker Photon II 14 – CPAD). The data were reduced using *SAINT* and corrected for absorption effects via the multiscan method (*SADABS*).[3] The structures were solved by direct methods (*SIR2002*, *SHELXS*)[4] and refined against all F^2 data by full-matrix least-squares techniques (*SHELXL*-2018/3)[5] minimizing $w[F_o^2-F_c^2]^2$. All non-hydrogen atoms were refined with anisotropic displacement parameters. Hydrogen atoms were included in calculated positions and allowed to ride on their respective carrier atoms, with the isotropic temperature factors U_{iso} fixed at 1.2 times (1.5 times for methyl groups) of the U_{eq} values of the respective carrier atoms.

The crystal structure of complex **4** crystallizes in the centrosymmetric space group $P2_{1/c}$. The asymmetric unit contain a single salt of the Ru(II) complex, consisting of a Cl^- anion (which exhibits a refined positional disorder of 10% with a Br^- anion originating from the imidazolium salt) and the cationic Ru(II)-CNC hydride complex. The crystal structure of complex **6** crystallizes in the centrosymmetric space group $C_{2/c}$. In the asymmetric unit, a single Ru(II)-CNC complex is observed, revealing that the methylene bridge of the CNC pincer ligand is mono-deprotonated. Neither of the crystal structures **4** nor **6** exhibits any noteworthy disorder. A search for solvent accessible voids in the crystal structure **4** was conducted using the Mask option implemented in Olex2 as an alternative to SQUEEZE.[6] The search revealed one small volume of potential solvent

---

2 Bruker APEX3 software suite; Bruker AXS, Inc.; Madison, WI 53711, 2016.

3 Bruker Advanced X-ray solutions. *SAINT* and *SADABS* programs. Bruker AXS Inc. Madison, WI 53711, 2012.

4 M. C. Burla, M. Camalli, B. Carrozzini, G. L. Cascarano, C. Giacovazzo, G. Polidori, R. Spagna. *J. Appl. Crystallogr.* **2003**, *36*, 1103-1104.

5 G. M. Sheldrick. *Acta Crystallogr. Sect. A Found. Crystallogr.* **2008**, *64*, 112-122.

6 P. v.d. Sluis, A. L. Spek, *Acta Crystallogr., Sect. A* **1990**, *46*, 194−201.

(1984 Å^3, corresponding to 139 electrons), though the solvent content could not be identified or refined with the most stringent restraints. The corresponding CIF data represent structures processed using SQUEEZE, where solvent molecules are treated as a diffuse contribution to the overall scattering. These solvent molecules are not assigned specific atomic positions and are excluded from the structural model. The SQUEEZE results were included as an appendix to the CIF.

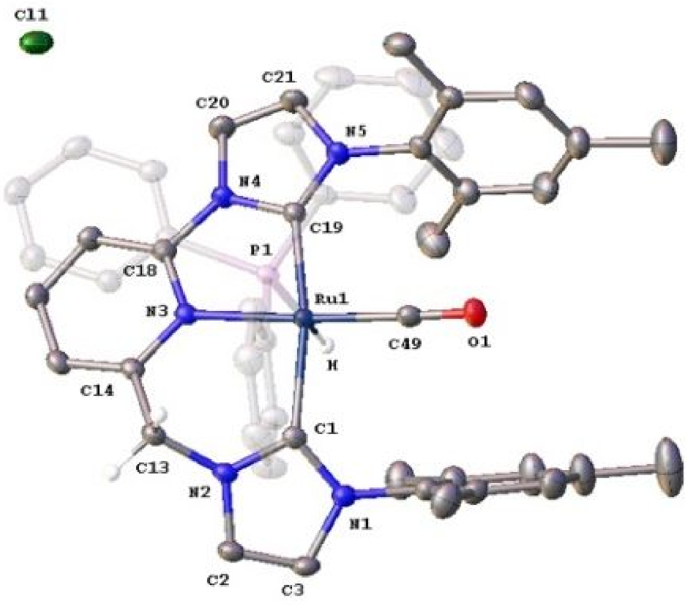

Figure S44. ORTEP view of molecular structure of salt complex **4,** with thermal ellipsoids shown at the 50% probability level. Hydrogen atoms, except for the Ru(II)-hydride and those on the C13 methylene bridge, have been omitted for clarity.

Table S1. Crystal data and structure refinement for **4**.

Empirical formula	$C_{49}H_{47}Br_{0.1}Cl_{0.9}N_5OPRu$
Formula weight	893.67
Temperature/K	193.00
Crystal system	monoclinic
Space group	$P2_1/c$
a/Å	15.8431(13)
b/Å	26.909(2)
c/Å	14.0672(12)
α/°	90
β/°	107.208(3)
γ/°	90
Volume/Å^3	5728.7(8)
Z	4
ρ_{calc}g/cm^3	1.036
μ/mm^{-1}	0.443
F(000)	1847.0
Crystal size/mm^3	0.45 × 0.38 × 0.1
Radiation	MoKα (λ = 0.71073)
2Θ range for data collection/°	3.728 to 50.5
Index ranges	-19 ≤ h ≤ 19, -32 ≤ k ≤ 32, -16 ≤ l ≤ 16
Reflections collected	78398
Independent reflections	10363 [R_{int} = 0.0427, R_{sigma} = 0.0250]
Data/restraints/parameters	10363/19/506
Goodness-of-fit on F^2	1.106

Final R indexes [I>=2σ (I)] $R_1 = 0.0363$, $wR_2 = 0.1152$

Final R indexes [all data] $R_1 = 0.0407$, $wR_2 = 0.1172$

Largest diff. peak/hole / e $Å^{-3}$ 0.65/-0.40

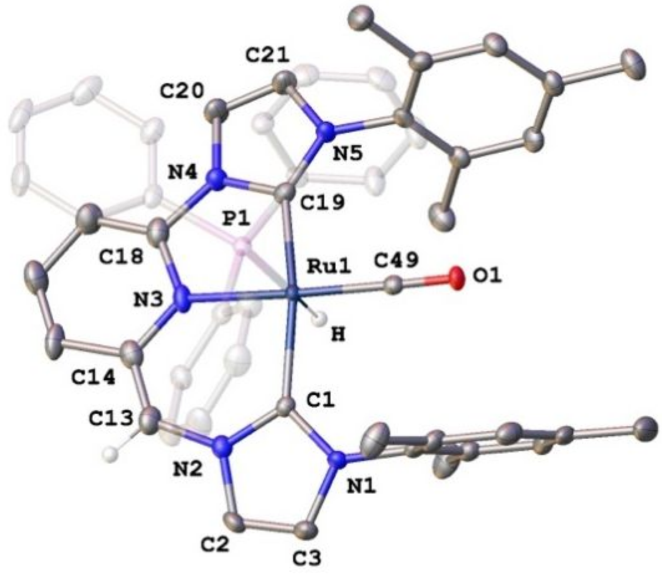

Figure S45. ORTEP view of molecular structure of complex **6,** with thermal ellipsoids shown at the 50% probability level. Hydrogen atoms, except for the Ru(II)-hydride and the hydrogen atom on the C13 methine bridge, have been omitted for clarity.

Table S2. Crystal data and structure refinement for **6**.

Empirical formula	$C_{49}H_{46}N_5OPRu$
Formula weight	852.95
Temperature/K	193.00
Crystal system	monoclinic
Space group	C2/c
a/Å	37.235(2)
b/Å	11.2929(7)
c/Å	21.2982(12)
α/°	90
β/°	114.665(2)
γ/°	90
Volume/Å^3	8138.5(8)
Z	8
ρ_{calc}g/cm^3	1.392
μ/mm^{-1}	0.469
F(000)	3536.0
Crystal size/mm^3	0.4 × 0.1 × 0.08
Radiation	MoKα (λ = 0.71073)
2Θ range for data collection/°	4.096 to 50.494
Index ranges	-44 ≤ h ≤ 44, -13 ≤ k ≤ 13, -25 ≤ l ≤ 24

Reflections collected	98841
Independent reflections	7359 [R_{int} = 0.0699, R_{sigma} = 0.0296]
Data/restraints/parameters	7359/1/524
Goodness-of-fit on F^2	1.122
Final R indexes [I>=2σ (I)]	R_1 = 0.0446, wR_2 = 0.0990
Final R indexes [all data]	R_1 = 0.0521, wR_2 = 0.1016
Largest diff. peak/hole / e Å^{-3}	1.10/-1.04

5. DFT Calculations.

DFT calculations were carried out using the Gaussian 09 program[7] with the B3LYP hybrid functional,[8] with dispersion effects taken into account by adding the D3 version of Grimme's empirical dispersion.[9] Geometry optimizations were performed without restrictions in bulk solvent (THF or toluene) with the SMD continuum model[10] and all atoms being represented with the Def2SVP basis set. Vibrational analysis was used at the same level of theory to characterize the

---

[7] Frisch, M. J.; Trucks, G. W.; Schlegel, H. B.; Scuseria, G. E.; Robb, M. A.; Cheeseman, J. R.; Scalmani, G.; Barone, V.; Petersson, G. A.; Nakatsuji, H.; Li, X.; Caricato, M.; Marenich, A.; Bloino, J.; Janesko, B. G.; Gomperts, R.; Mennucci, B.; Hratchian, H. P.; Ortiz, J. V.; Izmaylov, A. F.; Sonnenberg, J. L.; Williams-Young, D.; Ding, F.; Lipparini, F.; Egidi, F.; Goings, J.; Peng, B.; Petrone, A.; Henderson, T.; Ranasinghe, D.; Zakrzewski, V. G.; Gao, J.; Rega, N.; Zheng, G.; Liang, W.; Hada, M.; Ehara, M.; Toyota, K.; Fukuda, R.; Hasegawa, J.; Ishida, M.; Nakajima, T.; Honda, Y.; Kitao, O.; Nakai, H.; Vreven, T.; Throssell, K.; Montgomery, J. A., Jr.; Peralta, J. E.; Ogliaro, F.; Bearpark, M.; Heyd, J. J.; Brothers, E.; Kudin, K. N.; Staroverov, V. N.; Keith, T.; Kobayashi, R.; Normand, J.; Raghavachari, K.; Rendell, A.; Burant, J. C.; Iyengar, S. S.; Tomasi, J.; Cossi, M.; Millam, J. M.; Klene, M.; Adamo, C.; Cammi, R.; Ochterski, J. W.; Martin, R. L.; Morokuma, K.; Farkas, O.; Foresman, J. B.; Fox, D. J. *Gaussian 09*, revision E.01; Gaussian, Inc.: Wallingford, CT, 2016.

[8] (a) A. D. Becke. *J. Chem. Phys.* **1993**, *98*, 5648−5652. (b) C. Lee, W. Yang, R. G. Parr, *Phys. Rev. B: Condens. Matter Mater. Phys.* **1988**, *37*, 785−789. (c) B. Miehlich, A. Savin, H. Stoll, H. Preuss, *Chem. Phys. Lett.* **1989**, *157*, 200−206.

[9] S. Grimme, S. Ehrlich, L. Goerigk. *J. Comput. Chem.* **2011**, *32*, 1456−1465.

[10] A. V. Marenich, C. J. Cramer, D. G. Truhlar. *J. Phys. Chem. B* **2009**, *113*, 6378−6396.

stationary points in the potential energy surface, as well as for calculating the zero-point, enthalpy, and Gibbs energy corrections at 295 K and 1 atm. The nature of the intermediates connected by a given transition state along a reaction path was proven by intrinsic reaction coordinate (IRC) calculations or by perturbing the geometry of the TS along the reaction path eigenvector. The energies reported in the manuscript were obtained from single point calculations of the optimized geometries replacing the initial basis set with the triple-ζ quality basis Def2TZVP.[11]

[11] (a) F. Weigend, R. Ahlrichs. *Phys. Chem. Chem. Phys.* **2005**, *7*, 3297−3305. (b) F. Weigend, *Phys. Chem. Chem. Phys.* **2006**, *8*, 1057−1065.

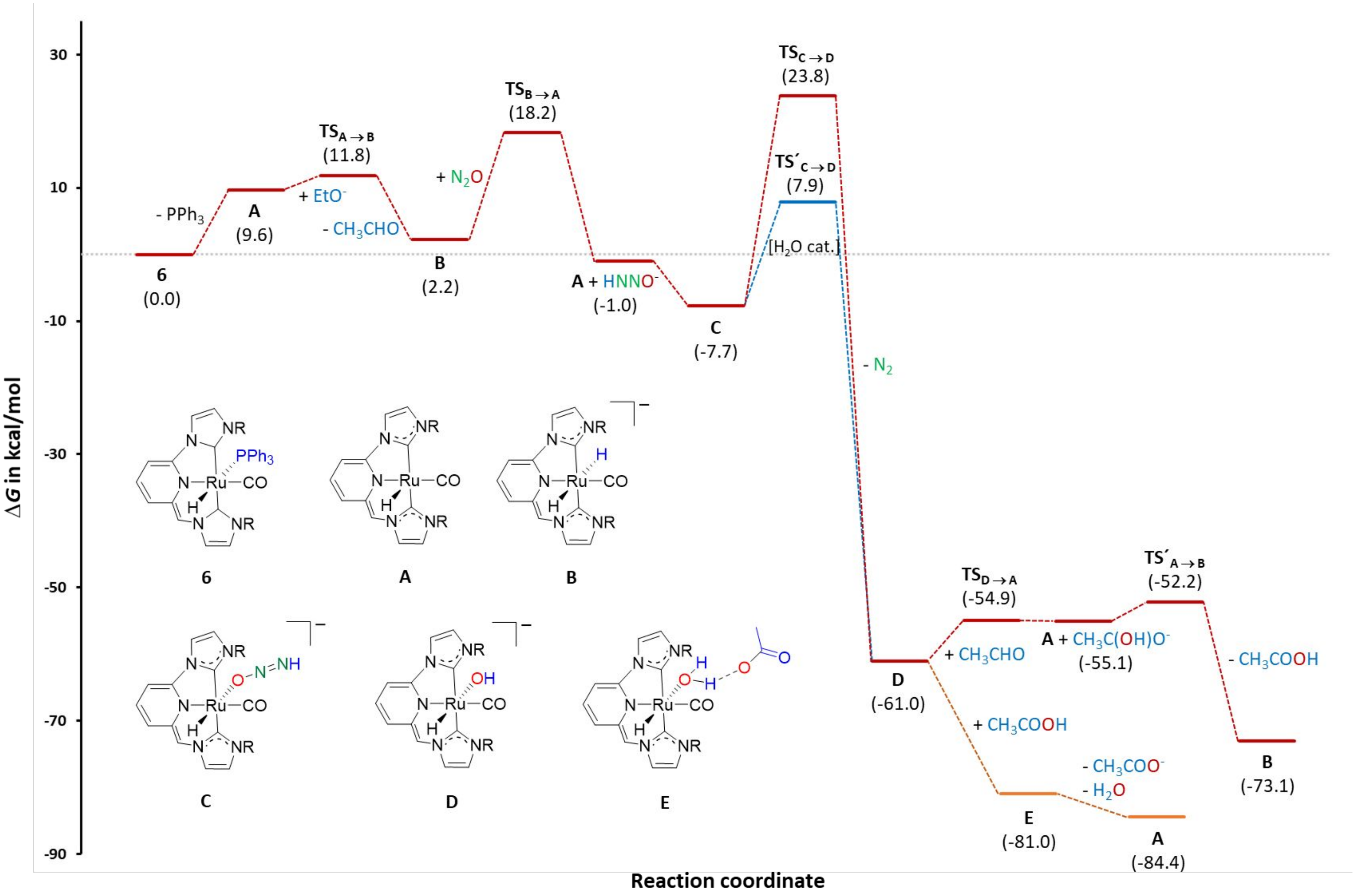

Figure S46. Overall DFT calculated free energy (ΔG in toluene, kcal/mol) profile of the oxidation of ethanol with N_2O.

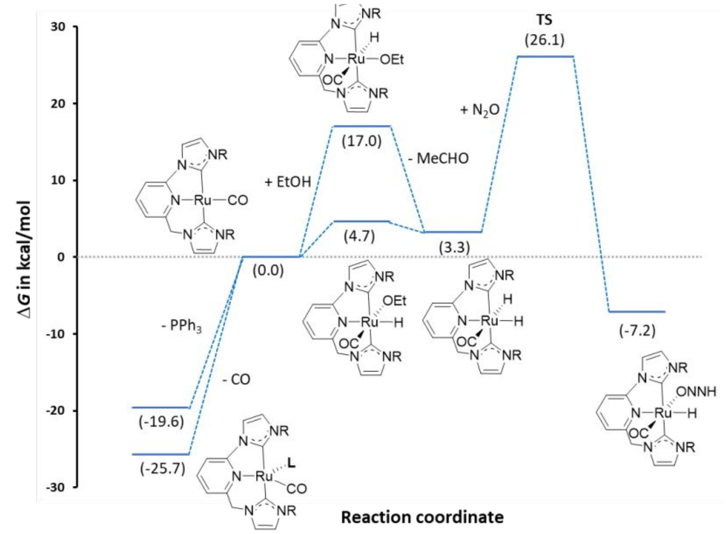

Figure S47. Partial DFT calculated free energy (ΔG in toluene, kcal/mol) profile of the oxidation of ethanol with N_2O assuming a Ru(0)/Ru(II) mechanism. [The energetic spans (δE) of the reaction profiles are 51.8 and 45.7 kcal/mol in the reactions catalyzed by **7** and **6-iso**, respectively].

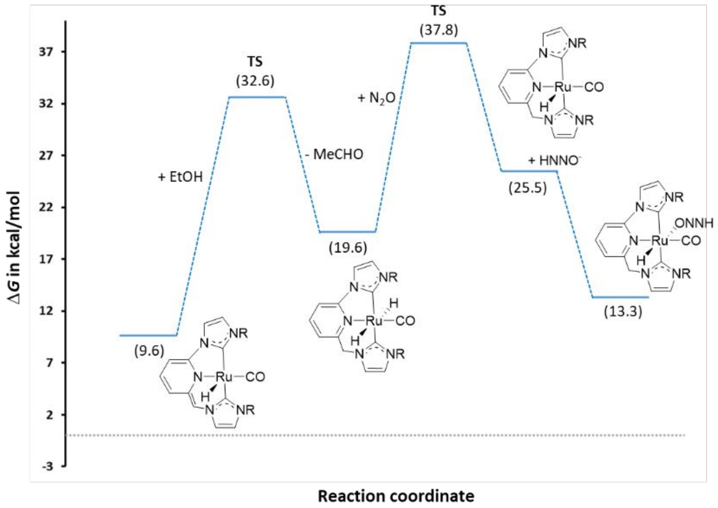

Figure S48. Partial DFT calculated free energy (ΔG in toluene, kcal/mol) profile of the oxidation of ethanol with N_2O assuming a ligand-assisted mechanism. [The energetic spans (δE) of the reaction profiles are 37.8 and 42.5 kcal/mol in the reactions catalyzed by **6** and **7-iso**, respectively].

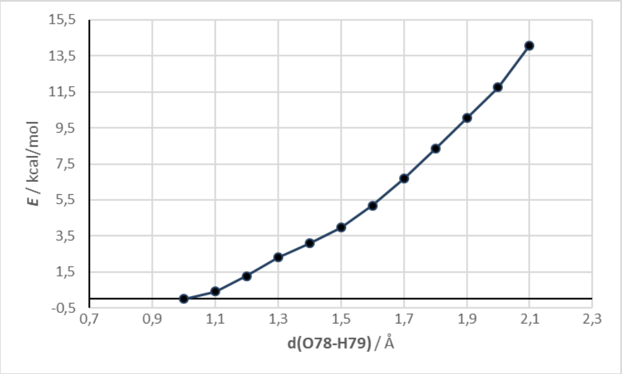

Figure S49. Potential energy scan (PES) of the protonation of the OH ligand of **D** by acetic acid to yield **E**. The horizontal axis represents the distance between O78-H79. [The scan suggests that this step takes place without an energy barrier associated to the proton transfer.]
